# A prospective case–cohort analysis of plasma metabolites and breast cancer risk

**DOI:** 10.1186/s13058-023-01602-x

**Published:** 2023-01-17

**Authors:** Victoria L. Stevens, Brian D. Carter, Eric J. Jacobs, Marjorie L. McCullough, Lauren R. Teras, Ying Wang

**Affiliations:** 1 Department of Population Sciences, American Cancer Society, 3380 Chastain Meadows Pkwy NW Suite 200, Kennesaw, GA 30144 USA; 2 Present Address: Social and Scientific Systems, DLH Holdings Corporation, Atlanta, GA USA

**Keywords:** Breast cancer, Metabolomics, Prospective study, Metabolites

## Abstract

**Background:**

Breast cancer incidence rates have not declined despite an improvement in risk prediction and the identification of modifiable risk factors, suggesting the need to identify novel risk factors and etiological pathways involved in this cancer. Metabolomics has emerged as a promising tool to find circulating metabolites associated with breast cancer risk.

**Methods:**

Untargeted metabolomic analysis was done on prediagnostic plasma samples from a case–cohort study of 1695 incident breast cancer cases and a 1983 women subcohort drawn from Cancer Prevention Study 3. The associations of 868 named metabolites (per one standard deviation increase) with breast cancer were determined using Prentice-weighted Cox proportional hazards regression modeling.

**Results:**

A total of 11 metabolites were associated with breast cancer at false discovery rate (FDR) < 0.05 with the majority having inverse association [ranging from RR = 0.85 (95% CI 0.80–0.92) to RR = 0.88 (95% CI 0.82–0.94)] and one having a positive association [RR = 1.14 (95% CI 1.06–1.23)]. An additional 50 metabolites were associated at FDR < 0.20 with inverse associations ranging from RR = 0.88 (95% CI 0.81–0.94) to RR = 0.91 (95% CI 0.85–0.98) and positive associations ranging from RR = 1.13 (95% CI 1.05–1.22) to RR = 1.11 (95% CI 1.02–1.20). Several of these associations validated the findings of previous metabolomic studies. These included findings that several progestogen and androgen steroids were associated with increased risk of breast cancer in postmenopausal women and four phospholipids, and the amino acids glutamine and asparagine were associated with decreased risk of this cancer in pre- and postmenopausal women. Several novel associations were also identified, including a positive association for syringol sulfate, a biomarker for smoked meat, and 3-methylcatechol sulfate and 3-hydroxypyridine glucuronide, which are metabolites of xenobiotics used for the production of pesticides and other products.

**Conclusions:**

Our study validated previous metabolite findings and identified novel metabolites associated with breast cancer risk, demonstrating the utility of large metabolomic studies to provide new leads for understanding breast cancer etiology. Our novel findings suggest that consumption of smoked meats and exposure to catechol and pyridine should be investigated as potential risk factors for breast cancer.

**Supplementary Information:**

The online version contains supplementary material available at 10.1186/s13058-023-01602-x.

## Background

Breast cancer is the most commonly diagnosed cancer and the second leading cause of cancer death among women in the USA [1]. Despite knowledge of several modifiable risk factors for this cancer [2], incidence rates for breast cancer have continued to rise over the past several years [3]. A better understanding of breast cancer etiology and the factors that affect this process could lead to the development of new prevention strategies and the identification of novel therapeutic targets for chemoprevention.

Metabolomics provides a comprehensive assessment of the small molecules in a blood sample that integrates the effects of endogenous metabolism, exogenous exposures, and genetic variation. Recently, this technology has been used in prospective cohort studies to identify metabolites associated with breast cancer risk. To date, there have been ten studies from seven prospective cohorts that have applied metabolomics to prediagnostic blood samples from breast cancer cases and controls [4–13]. These included both targeted metabolomics [4, 9, 12, 13] in which a defined set of metabolites were analyzed, and untargeted metabolomics [5–8, 10, 11], where all metabolites that can be measured were analyzed and used either nuclear magnetic resonance (NMR) [7, 11] or mass spectroscopy (MS) [4–6, 8–10, 12, 13] for the metabolite measurements. The number of breast cancer cases in these studies ranged from 100 [13] to 1997 [12], and the criteria used to define statistical significance for associations varied. While all the studies identified at least one metabolite associated with breast cancer, the only metabolites whose associations were directly replicated were the sulfated derivatives of the androgenic steroids, dehydroepiandrosterone (DHEA), and 3β, 17β-androstenediol [5, 6, 10]. This lack of replication could indicate that robust associations for metabolites with breast cancer do not exist, or it could be due to the small size of most of the studies and the limited overlap in the metabolites analyzed in each study [14]. Larger studies using untargeted platforms that maximize the coverage of metabolites are needed to resolve this issue.

In this study, we conducted a large prospective case–cohort analysis among 1695 breast cancer cases and a randomly selected subcohort of 1983 participants drawn from women enrolled in the Cancer Prevention Study-3 (CPS-3). Relative levels of 868 known metabolites were measured using an untargeted, MS-based metabolomics platform to maximize the chance of our findings overlapping with those of other studies and to discover novel metabolites associated with breast cancer risk.

## Methods

### Study population

The women in this study were from the CPS-3, a prospective study of cancer incidence and mortality among approximately 300,000 adults. CPS-3 participants were cancer-free, between the ages of 30 and 65, and from 35 states, Puerto Rico, and Washington DC at the time of enrollment between 2006 and 2013. Details about enrollment and cohort characteristics are available elsewhere [15]. All participants provided informed consent, a non-fasting blood sample, and completed a self-administered questionnaire requesting demographic, lifestyle, and medical information at enrollment. Blood was collected in an EDTA-containing vacutainer and was processed into plasma, red blood cells, and buffy coat within 24 h of collection. Blood fractions were frozen and stored in a biorepository in liquid nitrogen vapor phase tanks. All aspects of the CPS-3 study are approved by the Emory University Institutional Review Board.

Of the 303,682 participants enrolled in CPS-3, we excluded those missing a blood sample (*N* = 9534), who were not female (*N* = 70,596), had prevalent cancer other than nonmelanoma skin cancer (*N* = 2248), lived in a state not covered in our cancer registry linkage at the time of this analysis (*N* = 17,880), were missing birth date (*N* = 64), and whose enrollment was revoked or otherwise compromised (*N* = 166). From the 205,595 women who remained, 1695 were identified as having been diagnosed with invasive breast cancer between enrollment and December 31, 2015, through linkage to 36 state cancer registries. We also selected a random subcohort of 1983 women from the women eligible for the analysis, of whom 14 developed invasive breast cancer after enrollment. Comparison of the basic characteristics of subcohort with those of all the women in CPS-3 [15] indicates that it is representative of the women in the entire cohort.

### Metabolomic analyses

Metabolomic analyses of plasma samples were done by Metabolon, Inc. (Morrisville, NC) as previously described [16]. Metabolites were identified by comparison of ion features to a library of over 3300 chemical standards. Compounds with the same features for which the exact placement of side groups could not be assigned were given the same chemical name followed by a number in parentheses to distinguish them from one another. Metabolite peaks were quantified using the area under the curve. Metabolite levels below the limit of detection were assigned the minimum observed value measured. Day-to-day variation was corrected by dividing each metabolite by its median for each run-day. The reliability of the analyses was assessed using replicate quality control samples analyzed with the study samples. For the measured metabolites, the median technical intraclass correlations coefficient (ICC) was 0.79 with an interquartile range of 0.69 to 0.89.

The metabolomic analyses provided data on 1053 named metabolites. Of these, metabolites were excluded if they had an ICC < 0.50 (*N* = 70), if no results were obtained for them from any of the quality control samples (*N* = 52), or if they were missing in > 90% of the samples (*N* = 63). Thus, 868 metabolites were included in the analyses.

### Statistical analyses

Metabolite levels were log-transformed and auto-scaled (mean = 0, SD = 1) to approximate a normal distribution and be on the same scale [17]. With the case–cohort study design, multivariable-adjusted relative risks (RR) and 95% confidence intervals (CI) for the association of each metabolite (per one standard deviation diagnosis increase) with breast cancer was estimated using Prentice-weighted Cox proportional hazards regression models using time-in-study as the time axis. In these models, cases outside the subcohort contributed person-time only on their diagnosis date [18]. The women in the subcohort contributed to person time from the date of blood draw or collection of the baseline questionnaire, whichever came last, to date of breast cancer, death date, or December 31, 2015, whichever came first. Multivariable models were stratified on single year of age and adjusted for race, education, family history of breast cancer, age at menarche, oral contraceptive use, postmenopausal hormone use, and parity and age at first birth, all modeled as presented in Table 1. BMI was modeled as a continuous variable and, when missing, was imputed as the median of the entire study population. To account for multiple comparisons, a false discovery rate (FDR) < 0.05 was used to define statistical significance [19]. However, metabolites associated with breast cancer at FDR < 0.20 were also included in all analyses and tables to facilitate comparisons with results of previous studies that focused on metabolites in this range [5, 6, 8, 10] and because the expanded group of metabolites may provide more insight into the associations of the various metabolites.

**Table 1:** Selected characteristics of the women in the study

Characteristic	Breast cancer cases *N* = 1687	Subcohort *N* = 1983
	*N* (%)	*N* (%)
Age (year)		
< 40	148 (8.8)	496 (25.0)
40–49	498 (29.5)	527 (26.6)
50–59	716 (42.4)	693 (34.9)
≥ 60	327 (19.4)	267 (13.5)
Race		
White	1466 (86.8)	1653 (83.4)
Black	62 (3.7)	85 (4.3)
Hispanic	101 (6.0)	150 (7.6)
Other/Missing	60 (3.6)	95 (4.8)
Education		
High school or less	229 (13.6)	177 (8.9)
Some college/2-year degree	522 (30.9)	589 (29.7)
College graduate	461 (27.3)	631 (31.8)
Graduate degree	395 (23.4)	511 (25.8)
Missing/Unclear	82 (4.9)	75 (3.8)
Family history of breast cancer		
No	1099 (65.1)	1466 (73.9)
Yes	322 (19.1)	221 (11.1)
Missing	268 (15.9)	296 (14.9)
BMI category (kg/m^2^)		
< 18.5	9 (0.5)	26 (1.3)
18.5–22.4	296 (17.5)	419 (21.1)
22.5–24.9	297 (17.6)	415 (20.9)
25.0–29.9	529 (31.3)	512 (25.8)
≥ 30	542 (32.1)	593 (29.9)
Missing	16 (0.9)	18 (0.9)
Smoking status		
Never smoker	1085 (64.2)	1335 (67.3)
Current smoker	83 (4.9)	102 (5.1)
Former smoker	507 (30.0)	542 (27.3)
Missing	14 (0.8)	4 (0.2)
Time since last meal (h)	1.99	2.01
Time from blood draw to processing (h)	14.53	14.78
Age at menarche (year)		
≤ 11	389 (23.0)	423 (21.3)
12	461 (27.3)	545 (27.5)
13	476 (28.2)	578 (29.1)
≥ 14	328 (19.4)	402 (20.3)
Missing	35 (2.1)	35 (1.8)
Age at first birth and parity		
Nulliparous	308 (18.2)	437 (22.0)
Age < 25, 1–2 births	293 (17.3)	310 (15.6)
Age < 25, 3+ births	176 (10.4)	204 (10.3)
Age 25+ , 1–2 births	576 (34.1)	633 (31.9)
Age 25+ , 3+ births	138 (8.2)	165 (8.3)
Unknown	198 (11.7)	234 (11.8)
Age at menopause [mean, yr(SD)]	48.3 (6.5)	47.3 {6.9)
Menopausal status		
Premenopausal	731 (43.3)	1038 (52.3)
Postmenopausal	874 (51.7)	842 (42.5)
Unknown/Incomplete	84 (5.0)	103 (5.2)
Oral contraceptive use		
Never	182 (10.8)	206 (10.4)
Current	142 (8.4)	198 (10.0)
Former	1327 (78.6)	1542 (77.8)
Missing	38 (2.2)	37 (1.9)
Postmenopausal hormone use		
Never	1177 (69.7)	1486 (74.9)
Current	165 (9.8)	159 (8.0)
Former	230 (13.6)	241 (12.2)
Missing	117 (6.9)	97 (4.9)
Estrogen receptor status		
ER+	1410 (83.5)	14 (0.7)
ER−	252 (14.9)	0 (0)
Missing	27 (1.6)	0 (0)
Breast cancer stage		
Localized	1154 (68.3)	11 (0.6)
Regional	486 (28.8)	3 (0.2)
Distant	49 (2.9)	0 (0)
Unknown	0 (0)	0 (0)

*BMI* Body mass index; *ER* estrogen receptor

Stratified analyses were run to determine if metabolite associations varied by several parameters. For estrogen receptor (ER) status, independent models were run for ER+ and ER− breast cancer. *p* values for heterogeneity were calculated based on a meta-analysis of the results of the two models done using Cochran’s Q test [20]. For menopausal status and time since blood draw, an interaction term between the metabolite and the stratification variable was included in the model. A *p* value was calculated using the Likelihood Ratio test between the full model and a reduced model without the interaction term.

The clustered block analyses defined groups of metabolites mutually associated with breast cancer risk at FDR < 0.20 that could be represented by a single lead metabolite were done as described previously [21]. Briefly, hierarchical heat maps based on Pearson correlation coefficients, shown in Additional file 1: Fig. 1, were used to identify groups of metabolites with correlation coefficients ≥ 0.40. The metabolite most strongly associated with breast cancer in each group was defined as the lead metabolite for the group; whether it could represent the associations of all metabolites in the group was determined by rerunning the analyses controlling for that metabolite. If none of the associations were statistically significant (uncorrected *p* < 0.05), the group of metabolites was defined as a clustered block. Otherwise, the group of metabolites was split as suggested by the heatmap and the procedure was repeated until no significant associations remained.

**Fig. 1:**
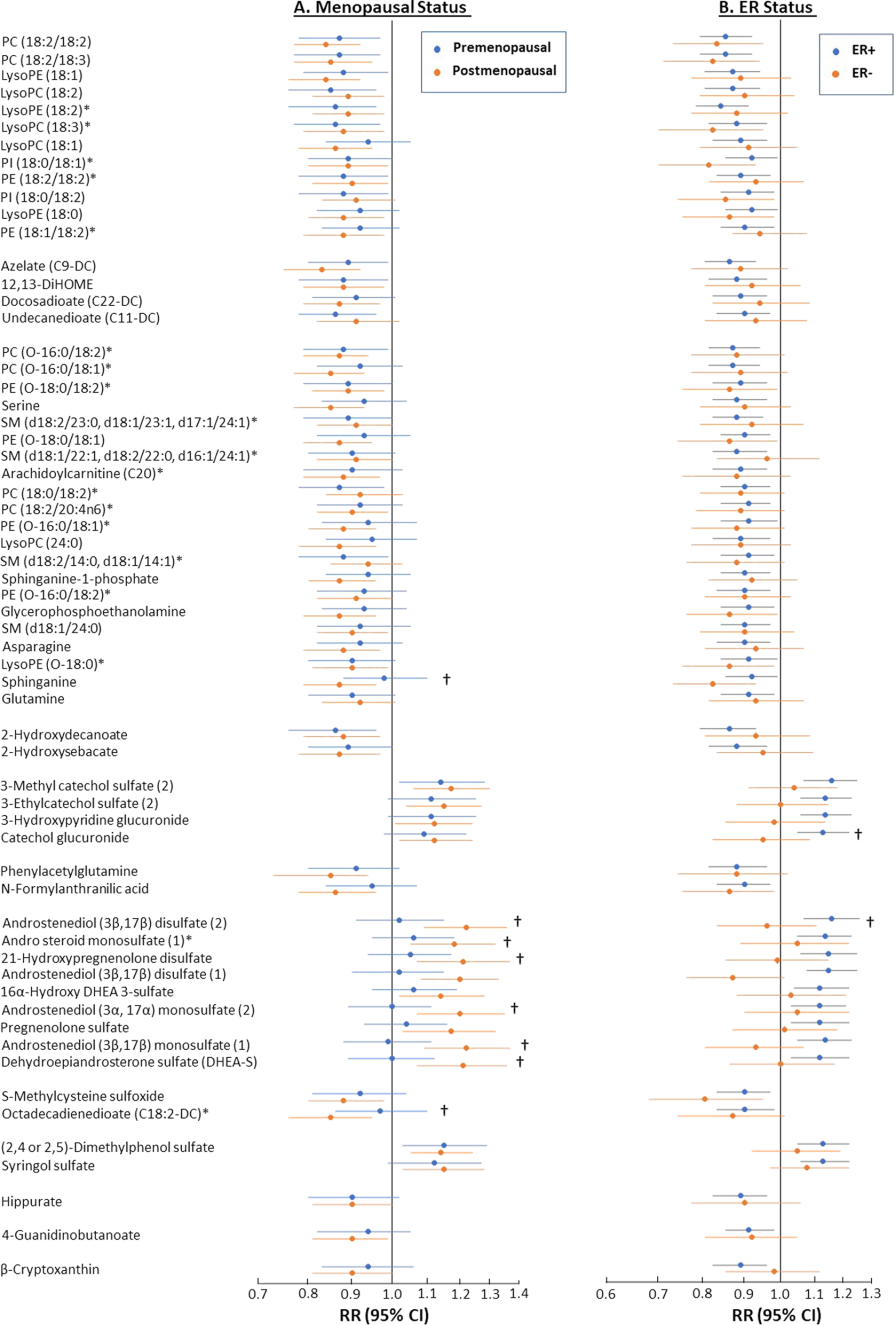
Stratified analyses of breast cancer associations. Associations of the metabolites associated with breast cancer at FDR < 0.20 grouped in correlated blocks (**A**) in pre- and postmenopausal women, and (**B**) in women with ER+ or ER− breast cancer. Associations marked with † differed significantly (*p* < 0.05) between the two groups. An * next to a metabolite name indicates a level two (putative annotation) compound identification, whereas level one (definitive) identification requires comparing two or more properties of the metabolite, such as retention time, m/z, or fragmentation mass spectrum, to those for an authentic chemical standard, level two (putative) identification requires comparison of only one of these properties

## Results

The characteristics of the women in the study are given in Table 1. The breast cancer cases were somewhat older than the subcohort, with an average age of 52.1 versus 48.3 years. The cases were slightly heavier, with an average BMI of 28.2 versus 27.7 kg/m^2^, and were more likely to be white or have a family history of breast cancer. The cases were also more likely to be parous, be ever users of postmenopausal hormones, and be less educated than the women in the subcohort.

Of the 868 metabolites in the analyses, 11 were associated with breast cancer with FDR < 0.05. These, along with 50 additional metabolites associated with FDR < 0.20, are listed in Table 2. Ten of the 11 metabolites with FDR < 0.05 were lipids and were inversely associated with breast cancer risk. The other significant metabolite was the xenobiotic 3-methyl catechol sulfate [2], which was associated with an increased risk of breast cancer. The associations for all 868 metabolites included in the analysis are shown in Additional file 1: Table 1.

**Table 2:** Metabolites associated with breast cancer at FDR < 0.20

Metabolite	Metabolite class	RR (95% CI)^a^	*p*	FDR
PC (18:2/18:2)	Lipid	0.85 (0.80, 0.92)	1.19 × 10^−5^	6.87 × 10^−3^
PC (18:2/18:3)*	Lipid	0.85 (0.79, 0.91)	1.58 × 10^−5^	6.87 × 10^−3^
Azelate (C9-DC)	Lipid	0.87 (0.81, 0.93)	1.17 × 10^−4^	3.38 × 10^−2^
PC (O-16:0/18:2)*	Lipid	0.87 (0.82, 0.94)	1.93 × 10^−4^	3.42 × 10^−2^
LysoPE (18:1)	Lipid	0.87 (0.81, 0.94)	2.23 × 10^−4^	3.42 × 10^−2^
2-Hydroxydecanoate	Lipid	0.87 (0.81, 0.94)	2.37 × 10^−4^	3.42 × 10^−2^
LysoPC (18:2)	Lipid	0.87 (0.81, 0.94)	2.81 × 10^−4^	3.49 × 10^−2^
PC (O-16:0/18:1)*	Lipid	0.88 (0.82, 0.94)	3.32 × 10^−4^	3.60 × 10^−2^
LysoPE (18:2)*	Lipid	0.88 (0.82, 0.94)	4.60 × 10^−4^	3.81 × 10^−2^
3-Methyl catechol sulfate (2)	Xenobiotic	1.14 (1.06, 1.23)	4.76 × 10^−4^	3.81 × 10^−2^
LysoPC (18:3)*	Lipid	0.87 (0.81, 0.94)	4.83 × 10^−4^	3.81 × 10^−2^
Phenylacetylglutamine	Peptide	0.88 (0.81, 0.95)	1.11 × 10^−3^	8.04 × 10^−2^
PE (O-18:0/18:2)*	Lipid	0.89 (0.83, 0.96)	1.41 × 10^−3^	9.40 × 10^−2^
Serine	Amino acid	0.89 (0.83, 0.96)	1.74 × 10^−3^	1.03 × 10^−1^
Androstenediol (3β,17β) disulfate (2)	Lipid	1.13 (1.05, 1.22)	1.86 × 10^−3^	1.03 × 10^−1^
2-Hydroxysebacate	Lipid	0.89 (0.83, 0.96)	1.89 × 10^−3^	1.03 × 10^−1^
SM (d18:2/23:0, d18:1/23:1, d17:1/24:1)*	Lipid	0.89 (0.83, 0.96)	2.31 × 10^−3^	1.13 × 10^−1^
PE (O-18:0/18:1)	Lipid	0.90 (0.83, 0.96)	2.41 × 10^−3^	1.13 × 10^−1^
Androsteroid monosulfate (1)*	Lipid	1.13 (1.04, 1.22)	2.58 × 10^−3^	1.13 × 10^−1^
12,13-DiHOME	Lipid	0.89 (0.83, 0.96)	2.61 × 10^−3^	1.13 × 10^−1^
21-Hydroxypregnenolone disulfate	Lipid	1.13 (1.04, 1.22)	2.90 × 10^−3^	1.14 × 10^−1^
LysoPC (18:1)	Lipid	0.90 (0.83, 0.96)	3.01 × 10^−3^	1.14 × 10^−1^
SM (d18:1/22:1, d18:2/22:0, d16:1/24:1)*	Lipid	0.90 (0.84, 0.96)	3.03 × 10^−3^	1.14 × 10^−1^
3-Ethylcatechol sulfate (2)	Xenobiotic	1.11 (1.04, 1.20)	3.34 × 10^−3^	1.14 × 10^−1^
3-Hydroxypyridine glucuronide	Xenobiotic	1.12 (1.04, 1.20)	3.47 × 10^−3^	1.14 × 10^−1^
Arachidyl carnitine (C20)*	Lipid	0.89 (0.82, 0.96)	3.55 × 10^−3^	1.14 × 10^−1^
S-Methylcysteine sulfoxide	Amino acid	0.89 (0.83, 0.96)	3.56 × 10^−3^	1.14 × 10^−1^
PC (18:0/18:2)*	Lipid	0.90 (0.84, 0.97)	3.75 × 10^−3^	1.15 × 10^−1^
(2,4 or 2,5)-Dimethylphenol sulfate	Xenobiotic	1.12 (1.04, 1.20)	4.09 × 10^−3^	1.15 × 10^−1^
*N*-Formylanthranilic acid	Amino acid	0.89 (0.83, 0.96)	4.09 × 10^−3^	1.15 × 10^−1^
Octadecadienoate (C18:2-DC)*	Lipid	0.89 (0.83, 0.96)	4.13 × 10^−3^	1.15 × 10^−1^
Syringol sulfate	Xenobiotic	1.12 (1.04, 1.21)	4.23 × 10^−3^	1.15 × 10^−1^
Docosadioate (C22-DC)	Lipid	0.90 (0.83, 0.97)	4.77 × 10^−3^	1.24 × 10^−1^
PC (18:2/20:4n6)*	Lipid	0.90 (0.84, 0.97)	5.02 × 10^−3^	1.24 × 10^−1^
PE (O-16:0/18:1)*	Lipid	0.90 (0.84, 0.97)	5.10 × 10^−3^	1.24 × 10^−1^
PI (18:0/18:1)*	Lipid	0.90 (0.83, 0.97)	5.25 × 10^−3^	1.24 × 10^−1^
LysoPC (24:0)	Lipid	0.90 (0.83, 0.97)	5.48 × 10^−3^	1.24 × 10^−1^
Hippurate	Xenobiotic	0.90 (0.83, 0.97)	5.86 × 10^−3^	1.24 × 10^−1^
SM (d18:2/14:0, d18:1/14:1)*	Lipid	0.90 (0.84, 0.97)	5.89 × 10^−3^	1.24 × 10^−1^
Androstenediol (3β,17β) disulfate (1)	Lipid	1.11 (1.03, 1.20)	5.99 × 10^−3^	1.24 × 10^−1^
PE (18:2/18:2)*	Lipid	0.90 (0.84, 0.97)	5.99 × 10^−3^	1.24 × 10^−1^
Sphinganine-1-phosphate	Lipid	0.91 (0.85, 0.97)	6.07 × 10^−3^	1.24 × 10^−1^
PI (18:0/18:2)	Lipid	0.90 (0.84, 0.97)	6.18 × 10^−3^	1.24 × 10^−1^
PE (O-16:0/18:2)*	Lipid	0.90 (0.84, 0.97)	6.30 × 10^−3^	1.24 × 10^−1^
Glycerophosphoethanolamine	Lipid	0.91 (0.84, 0.97)	7.09 × 10^−3^	1.34 × 10^−1^
16α-Hydroxy DHEA 3-sulfate	Lipid	1.11 (1.03, 1.20)	7.15 × 10^−3^	1.34 × 10^−1^
SM (d18:1/24:0)	Lipid	0.91 (0.84, 0.97)	7.25 × 10^−3^	1.34 × 10^−1^
Undecanedioate (C11-DC)	Lipid	0.91 (0.84, 0.97)	8.01 × 10^−3^	1.41 × 10^−1^
Asparagine	Amino acid	0.91 (0.84, 0.97)	8.05 × 10^−3^	1.41 × 10^−1^
Androstenediol (3α, 17α) monosulfate (2)	Lipid	1.11 (1.03, 1.20)	8.15 × 10^−3^	1.41 × 10^−1^
Catechol glucuronide	Amino acid	1.10 (1.02, 1.19)	8.85 × 10^−3^	1.51 × 10^−1^
Pregnenolone sulfate	Lipid	1.11 (1.03, 1.20)	9.72 × 10^−3^	1.60 × 10^−1^
4-Guanidinobutanoate	Amino acid	0.91 (0.85, 0.98)	9.82 × 10^−3^	1.60 × 10^−1^
LysoPE (O-18:0)*	Lipid	0.91 (0.84, 0.98)	9.94 × 10^−3^	1.60 × 10^−1^
LysoPE (18:0)	Lipid	0.91 (0.84, 0.98)	1.05 × 10^−2^	1.66 × 10^−1^
Androstenediol (3β,17β) monosulfate (1)	Lipid	1.11 (1.02, 1.19)	1.08 × 10^−2^	1.67 × 10^−1^
Sphinganine	Lipid	0.91 (0.85, 0.98)	1.10 × 10^−2^	1.67 × 10^−1^
β-Cryptoxanthin	Cofactors/vitamins	0.91 (0.84, 0.98)	1.33 × 10^−2^	1.94 × 10^−1^
PE (18:1/18:2)*	Lipid	0.91 (0.85, 0.98)	1.34 × 10^−2^	1.94 × 10^−1^
Dehydroepiandrosterone sulfate (DHEA-S)	Lipid	1.11 (1.02, 1.20)	1.35 × 10^−2^	1.94 × 10^−1^
Glutamine	Amino acid	0.91 (0.85, 0.98)	1.37 × 10^−2^	1.94 × 10^−1^

*FDR* false discovery rate; *RR* relative risk; *CI* confidence interval

^a^Models were adjusted for age, race, education, family history of breast cancer, age at menarche, oral contraceptive use, postmenopausal hormone use, and parity and age at first birth. Associations are per one standard deviation increase in metabolite level

As shown in Additional file 1: Table 2, 58 of the 61 metabolites associated with breast cancer at FDR < 0.20 clustered into 10 blocks of mutually associated metabolites. The largest block included 21 phospholipids, lysophospholipids, sphingomyelins, plasmalogens, and amino acids. The other clustered blocks ranged in size from 2 to 12 metabolites with members of each cluster mostly either structurally or functionally similar. Three metabolites were not clustered with any other metabolites.

Adjusting for BMI had no meaningful effect on the point estimates for the associations of the top metabolites with breast cancer (shown in Additional file 1: Table 3), although statistical significance was attenuated.

**Table 3:** Summary of metabolites previously replicated, newly replicated, or newly associated with breast cancer risk

Metabolite	Class	Results from prospective studies^a^					
		PLCO [5, 6]	SU.VI.MAX [7, 8]	EPIC^b^ [9]	CPS-II [10]	E3N [11]	CPS-3
**Previously replicated**^c^							
Androstenediol (3β,17β) disulfate (1)	Lipid	↑			↑		↑
16α-Hydroxy DHEA 3-sulfate	Lipid	**↑**			↑		↑
Androstenediol (3β,17β) monosulfate (2)	Lipid	↑			↑		–
PC (O-16:0/18:2)	Lipid			↓(C34:2)	↓		**↓**
Glutamine	Amino Acid	–	↑	↓	–	**↑**	↓
**New replications**^d^							
Dehydroepiandrosterone sulfate (DHEA-S)	Lipid	↑			–		↑
Androsteroid monosulfate (1)	Lipid	–			↑		↑
Androstenediol (3β,17β) disulfate (2)	Lipid	↑			–		↑
Androstenediol (3β,17β) monosulfate (1)	Lipid	↑			–		↑
Asparagine	Amino Acid	–		↓	–		↓
PC (18:0/18:2)	Lipid	–		↓(C36:2)	–		↓
LysoPE (O-18:0)	Lipid	–			↓		↓
LysoPC (18:2)	Lipid	–		↓	–		**↓**
**Novel Associations**^e^							
Octadecadienoate (C18:2-DC)	Lipid						↓
2-Hydroxysebacate	Lipid						↓
Syringol sulfate	Xenobiotic						↑
3-Methylcatechol sulfate (2)	Xenobiotic						**↑**
3-Hydroxypyridine glucuronide	Xenobiotic						↑

*PLCO* Prostate, Lung, Colorectal, and Ovarian Cancer screening trial; *SU.VI.MAX* Supplémentation en Vitamines et Minéraux Antioxydants cohort: *EPIC* European Prospective Investigation into Cancer study; *CPS-II* Cancer Prevention Study II; *E3N* Etude Epidémiologique auprès de femmes de la MGEN (Mutuelle Générale de l’Education Nationale) cohort; *CPS-3* Cancer Prevention Study 3; *ref* reference

^a^The reference for each cohort from which the results shown were taken are shown below the cohort’s name. The direction of the association reported is shown by the arrow with ↑ indicating a direct association and ↓ indicating an inverse association. The statistical significance of the association is indicated by whether the arrow is or is not in bold print with unbolded arrows corresponding to FDR < 0.20 and bold arrows corresponding to FDR < 0.05. A — indicates that the metabolite was measured in the study but not associated with breast cancer risk. A blank space indicates that the metabolite was not measured in that study

^**b**^The metabolomics platform used in the EPIC studies provided information on the chain lengths and saturation of the fatty acids in the sn1 and sn2 positions of phospholipids cumulatively rather than individually. Therefore, the lipids found associated in the EPIC studies may include other species in addition to those measured in our study

^c^Metabolites were associated with breast cancer risk in two or more previous studies

^d^Metabolites associated with breast cancer risk at FDR < 0.20 in this study that replicated previous findings

^e^Metabolites associated with breast cancer risk at FDR < 0.20 in this study not measured in any previous study

Results stratified by menopausal or ER status for the 61 metabolites are presented in Additional file 1: Tables S4 and S5, respectively, and grouped into clustered blocks in Fig. 1. The associations were significantly different (*p* < 0.05) by menopausal status for 8 of the 9 steroids and the lipids octadecadienoate (C18:2-DC) and sphinganine (Fig. 1A). The associations were stronger in postmenopausal women for all the metabolites. The associations of two metabolites, androstenediol (3β, 17β) disulfate [2] and catechol glucuronide, were significantly higher among ER+ than ER− breast cancer cases (Fig. 1B).

**Fig. 2:**
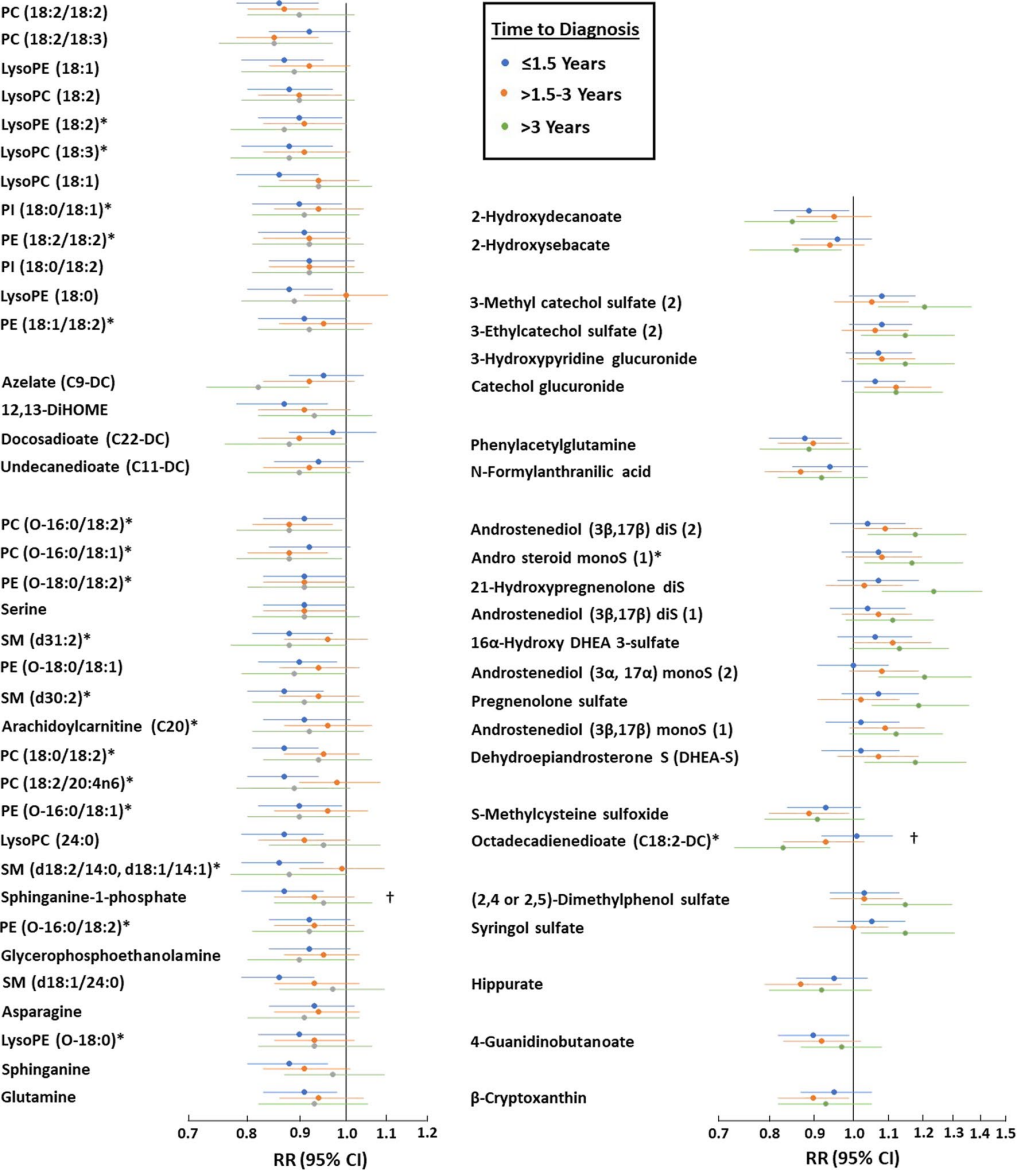
Influence of time from blood draw to diagnosis on breast cancer associations. Association for the metabolites associated with breast cancer at FDR < 0.20 grouped in correlated blocks stratified by time between blood collection and breast cancer diagnosis. Associations marked with † differed significantly (*p* < 0.05) between the three strata

To investigate whether the associations of the metabolites with breast cancer varied by the time between blood collection and diagnosis, estimates were calculated for three-time strata (complete results are in Additional file 1: Table S6). As shown in Fig. 2, the association of several metabolites varied by time between blood collection and breast cancer diagnosis. However, the difference was only significant for sphinganine-1-phosphate, for which the association was strongest in cases diagnosed within 1.5 years of blood collection and was attenuated in the later follow-up intervals, and octadecadienoate (C18:2-DC), for which the opposite trend was seen.

Finally, the use of exogenous hormones alters the association of some known risk factors with breast cancer [22]. Sensitivity analyses excluding current users of exogenous hormones resulted in only very small changes in the metabolite breast cancer associations (data not shown).

## Discussion

This prospective metabolomic analysis is among the largest done to date both in terms of the study population and the number of metabolites queried. Eleven metabolites were associated with breast cancer risk at FDR < 0.05 and an additional 50 metabolites were associated at a relaxed threshold of FDR < 0.20. These results replicated some previous studies and identified some novel associations.

The metabolites associated with breast cancer risk and that either replicate previous results or are novel findings are summarized in Table 3. The previously replicated metabolites which were associated with an increased risk of breast cancer were three androgenic steroids derived from DHEA [6, 10]. Two of these three steroids, androstenediol (3β,17β) disulfate [1] and 16α-hydroxy DHEA 3-sulfate, were associated with an increased risk of breast cancer in CPS-3. Four additional steroids, DHEA-S, androsteroid monosulfate [1], androstenediol (3β,17β) disulfate [2], and androstenediol (3β,17β) monosulfate [1], were also associated with an increased risk of breast cancer in CPS-3. These results, as well as the finding that the associations were only with postmenopausal breast cancer, are consistent with findings from other studies of circulating steroids [23–25]. Most studies of steroid metabolites in breast cancer have focused on androgens such as DHEA as the key metabolites influencing estrogen metabolism [26]. However, the correlated group of steroid metabolites we identified included two metabolites of pregnenolone (21-hydroxypregnenolone and pregnenolone sulfate), which is a precursor to the androgenic steroids. This suggests that the alteration in the rate of formation of pregnenolone from cholesterol, which is a highly regulated reaction and the rate-limiting step in steroid hormone biosynthesis [27], may play a role in breast cancer etiology.

One other metabolite that has potentially been replicated by previous studies [9, 10] is the plasmalogen phosphatidylcholine (PC) (O-16:0/18:2). Our findings for this metabolite directly replicate the finding from the CPS-II study [10]. In the European Prospective Investigation into Cancer (EPIC) study [9], which used the targeted Biocrates metabolomics platform, PC (O-16:0/18:2) was not specifically measured. However, all PC plasmalogens with 34 carbons and two double bonds, which include PC (O-16:0/18:2), were associated with breast cancer risk. Overall, the glycerophospholipids and sphingolipids we found to be associated with breast cancer clustered into two correlated blocks and included three lipids [PC (18:0/18:2), lyso-phosphatidylethanolamine (PE) (O-18:0) and lysoPC (18:2)] that replicated findings from previous studies [9, 10] for the first time. Why elevated levels of the lipids would be associated with reduced breast cancer risk is not clear. However, they are all common components of cellular membranes, and their altered levels could reflect the perturbation of pathways for membrane synthesis.

We found that glutamine was associated with a reduced risk of breast cancer, but previous studies have found conflicting results. Glutamine was associated with increased risk in the Supplémentation en Vitamines et Minéraux Antioxydants (SU.VI.MAX) cohort [8] where it was reported as glutamine/isoglutamine, and in the Etude Epidémiologique auprès de femmes de la MGEN (Mutuelle Générale de l’Education Nationale) (E3N) cohort [11], where the association was limited to premenopausal women. Glutamine was associated with a reduced risk of breast cancer in studies with both pre- and postmenopausal women in EPIC [9] and our study. Additional studies are needed to confirm the association of glutamine with breast cancer risk. However, the finding of an inverse association for asparagine, which is synthesized from glutamine, here and in the EPIC study [9] supports an inverse association for glutamine as higher levels of one of these amino acids should result in higher levels of the other. Neither of the studies that found a direct association for glutamine included asparagine among the metabolites analyzed.

We found associations between breast cancer risk and several metabolites that had not been included in previous studies. These metabolites are listed as novel associations in Table 3. Two metabolites, both decarboxylated fatty acids (octadecadienoate and 2-hydroxysebacate), were associated with decreased risk while the other three were associated with increased risk of breast cancer. One of these three, syringol sulfate, is a metabolite of syringol, which is a biomarker of smoked meat consumption [28]. A recent meta-analysis found that higher consumption of either red or processed meat was associated with a greater risk of breast cancer [29] but did not study smoked meat consumption specifically. Our findings for syringol sulfate argue that this issue should be investigated further.

The other two novel associations we observed were for the xenobiotics catechol glucuronide and 3-hydrixypyridine glucuronide, which were highly correlated (*r* = 0.76) and are metabolites of catechol and pyridine, respectively. While both compounds occur naturally at low levels, they are produced synthetically in large amounts. About half of the catechol and pyridine and catechol produced is used to make pesticides, while smaller amounts are used for pharmaceuticals and flavoring agents [30, 31]. Pyridine is also used in organic chemistry and in dyes [31], and both compounds have been found in cigarette smoke. The International Agency for Research on Cancer (IARC) evaluated the carcinogenicity of catechol, in 1999 [32], and pyridine, in 2019 [31], using primarily animal data and classified both as 2B, possibly carcinogenic to humans. Our findings suggest that further investigation into the carcinogenicity of these compounds is warranted.

In addition to several steroid metabolites, the associations of two additional metabolites [sphinganine and octadecadienoate (C18:2-DC)*] differed significantly (*p* < 0.05) in pre- and postmenopausal women. Two metabolites (androstenediol (3β,17β) disulfate [2] and catechol glucuronide) differed significantly between women with ER+ and ER− breast cancer. It is unclear why these associations differ by menopausal or ER status. These findings may be due to chance and require replication in future analyses.

A significant portion of the cases in this study were diagnosed with breast cancer within a few years after the blood collection, while others occurred later in follow-up, allowing us to explore if associations varied by time between blood collection and diagnosis. Only two metabolites had associations that varied significantly (*p* < 0.05) by time between blood draw and diagnosis, thus limiting any conclusions as to whether any of metabolite levels might be affected by reverse causation.

Although all the risk estimates remained similar, adjustment for BMI attenuated the associations of all the metabolites with breast cancer. This could indicate that BMI is a mediator of the associations. If so, then adjustment for BMI may be inappropriate. This possibility should be investigated further in future analyses.

A strength of this study is the large study population and the large number of identified metabolites measured. The factors likely contributed to our finding of 11 metabolites associated with breast cancer risk at FDR < 0.05, which is more than previous studies which identified one or two metabolites at most at this significance level [9, 10]. Limitations of our study include the fact the results were based on a single blood sample for each study participant. However, evidence suggests that levels of most circulating metabolites are relatively stable for up to 2 years [33, 34], suggesting that a single sample may be sufficient. Other limitations include smaller numbers in the subgroups used in the stratified analyses. Finally, although Black and Hispanic women were included in our study, there were too few to determine if associations differed by race and/or ethnicity. Thus, our findings may not be generalizable to all groups.

## Conclusions

This metabolomic study of breast cancer further replicated positive associations for several steroid metabolites that had been previously replicated and provided new replications for inverse associations for some lipids and amino acids. We also found novel associations for some metabolites which suggest new avenues for investigation into potentially modifiable risk factors for breast cancer. The associations of metabolites of syringol, catechol, and pyridine with increased breast cancer risk suggest future etiologic research should focus on smoked meat consumption and exposure to some chemicals found in our environment. Finally, the growing evidence that larger metabolomic studies are needed to identify robust associations suggests that additional studies and pooled analyses of existing results are needed.

## Supplementary Information

**Additional file 1: Figure 1.** Hierarchical heat map based on Pearson correlation coefficients of metabolites associated with breast cancer at FDR < 0.20 in analyses adjusted for age, race, education, family history of breast cancer, age at menarche, OC use, and parity.

## Data Availability

Data and supporting materials are not publicly available but may be available from the corresponding author upon reasonable request and approval of the Cancer Prevention Study 3 investigators.

## Abbreviations

RR: Relative risk
CI: Confidence interval
FDR: False discovery rate
NMR: Nuclear magnetic resonance
MS: Mass spectroscopy
ICC: Intraclass correlation coefficient
DHEA: Dehydroepiandrosterone
CPS: Cancer Prevention Study
ER: Estrogen receptor
EPIC: European Prospective Investigation into Cancer
PC: Phosphatidylcholine
PE: Phosphatidylethanolamine
SU.VI.MAX: Supplémentation en Vitamines et Minéraux Antioxydants
EPIC: European Prospective Investigation into Cancer
E3N: Etude Epidémiologique auprès de femmes de la MGEN (Mutuelle Générale de l’Education Nationale)
IARC: International Agency for Research on Cancer
PLCO: Prostate, Lung, Colorectal, and Ovarian Cancer screening trial
