# Correction: A prospective case–cohort analysis of plasma metabolites and breast cancer risk

**DOI:** 10.1186/s13058-023-01613-8

**Published:** 2023-02-01

**Authors:** Victoria L. Stevens, Brian D. Carter, Eric J. Jacobs, Marjorie L. McCullough, Lauren R. Teras, Ying Wang

**Affiliations:** 1 Department of Population Sciences, American Cancer Society, 3380 Chastain Meadows Pkwy NW Suite 200, Kennesaw, GA 30144 USA; 2 Social and Scientific Systems, DLH Holdings Corporation, Atlanta, GA USA

**Correction: Breast Cancer Res 25, 5 (2023)** **https://doi.org/10.1186/s13058-023-01602-x**

Following publication of the original article [1], the authors would like to correct the Acknowledgements section.

The sentence currently reads:

Not applicable.

The sentence should read:

The authors express sincere appreciation to all Cancer Prevention Study-3 participants, and to each member of the study and biospecimen management group. The authors would like to acknowledge the contribution to this study from central cancer registries supported through the Centers for Disease Control and Prevention's National Program of Cancer Registries and cancer registries supported by the National Cancer Institute's Surveillance Epidemiology and End Results Program.

The original article [1] has been corrected.
