# Supplementary material for: A prospective case–cohort analysis of plasma metabolites and breast cancer risk
Source: Breast Cancer Res. 2023 Jan 17;25:5. doi: 10.1186/s13058-023-01602-x (PMC9847033; doi:10.1186/s13058-023-01602-x)
Supplement: Supplementary file 1 — Additional file 1: Figure 1. Hierarchical heat map based on Pearson correlation coefficients of metabolites associated with breast cancer at FDR < 0.20 in analyses adjusted for age, race, education, family history of breast cancer, age at menarche, OC use, and parity. [file 13058_2023_1602_MOESM1_ESM.pdf]

Supplemental Table S1. Associations of all metabolites with breast cancer

Metabolite	SUPER PATHWAY	SUBPATHWAY	RR (95% CI)†	P	FDR	PLATFORM	HMDB	PUBCHEM	CHEM_ID
PC (18:2/18:2)	Lipid	Phosphatidylcholine (PC)	0.85 (0.80, 0.92)	1.19E-05	6.87E-03	LC/MS Pos Late	HMDB08138	5288075	100008903
PC (18:2/18:3)*	Lipid	Phosphatidylcholine (PC)	0.85 (0.79, 0.91)	1.58E-05	6.87E-03	LC/MS Pos Late	HMDB08141		100009343
Azelate (C9-DC)	Lipid	Fatty Acid, Dicarboxylate	0.87 (0.81, 0.93)	1.17E-04	3.38E-02	LC/MS Polar	HMDB00784	2266	2029
PC (O-16:0/18:2)*	Lipid	Plasmalogen	0.87 (0.82, 0.94)	1.93E-04	3.42E-02	LC/MS Pos Late	HMDB11211		100009009
LysoPE (18:1)	Lipid	Lysophospholipid	0.87 (0.81, 0.94)	2.23E-04	3.42E-02	LC/MS Pos Late	HMDB11506	9547071	100001569
2-Hydroxydecanoate	Lipid	Fatty Acid, Monohydroxy	0.87 (0.81, 0.94)	2.37E-04	3.42E-02	LC/MS Neg		21488	100004089
LysoPC (18:2)	Lipid	Lysophospholipid	0.87 (0.81, 0.94)	2.81E-04	3.49E-02	LC/MS Pos Late	HMDB10386	11988421	100001395
PC (O-16:0/18:1)*	Lipid	Plasmalogen	0.88 (0.82, 0.94)	3.32E-04	3.60E-02	LC/MS Pos Late			100009007
LysoPE (18:2)*	Lipid	Lysophospholipid	0.88 (0.82, 0.94)	4.60E-04	3.81E-02	LC/MS Pos Late	HMDB11507	52925130	100001570
3-Methyl catechol sulfate (2)	Xenobiotics	Benzoate Metabolism	1.14 (1.06, 1.23)	4.76E-04	3.81E-02	LC/MS Neg			100004110
LysoPC (18:3)*	Lipid	Lysophospholipid	0.87 (0.81, 0.94)	4.83E-04	3.81E-02	LC/MS Pos Late	HMDB10388		100005350
Phenylacetylglutamine	Peptide	Acetylated Peptides	0.88 (0.81, 0.95)	1.11E-03	8.04E-02	LC/MS Neg	HMDB06344	92258	100001417
PE (O-18:0/18:2)*	Lipid	Plasmalogen	0.89 (0.83, 0.96)	1.41E-03	9.40E-02	LC/MS Pos Late	HMDB11376		100009225
Serine	Amino Acid	Glycine, Serine and Threonine Metabolism	0.89 (0.83, 0.96)	1.74E-03	1.03E-01	LC/MS Pos Early	HMDB00187	5951	503
Androstenediol (3β,17β) disulfate (2)	Lipid	Androgenic Steroids	1.13 (1.05, 1.22)	1.86E-03	1.03E-01	LC/MS Neg			100001994
2-Hydroxysebacate	Lipid	Fatty Acid, Dicarboxylate	0.89 (0.83, 0.96)	1.89E-03	1.03E-01	LC/MS Neg	HMDB00424	128458	100020378
SM (d18:2/23:0, d18:1/23:1, d17:1/24:1)*	Lipid	Sphingomyelins	0.89 (0.83, 0.96)	2.31E-03	1.13E-01	LC/MS Pos Late			100008956
PE (O-18:0/18:1)	Lipid	Plasmalogen	0.90 (0.83, 0.96)	2.41E-03	1.13E-01	LC/MS Pos Late	HMDB11375		100008919
Andro steroid monosulfate C19H28O6S (1)*	Lipid	Androgenic Steroids	1.13 (1.04, 1.22)	2.58E-03	1.13E-01	LC/MS Neg			100002152
12,13-DiHOME	Lipid	Fatty Acid, Dihydroxy	0.89 (0.83, 0.96)	2.61E-03	1.13E-01	LC/MS Neg	HMDB04705	10236635	62
21-Hydroxypregnenolone disulfate	Lipid	Pregnenolone Steroids	1.13 (1.04, 1.22)	2.90E-03	1.14E-01	LC/MS Neg		134595	100001999
LysoPC (18:1)	Lipid	Lysophospholipid	0.90 (0.83, 0.96)	3.01E-03	1.14E-01	LC/MS Pos Late	HMDB02815	16081932	100001272
SM (d18:1/22:1, d18:2/22:0, d16:1/24:1)*	Lipid	Sphingomyelins	0.90 (0.84, 0.96)	3.03E-03	1.14E-01	LC/MS Pos Late	HMDB12104		100006295
3-Ethylcatechol sulfate (2)	Xenobiotics	Food Component/Plant	1.11 (1.04, 1.20)	3.34E-03	1.14E-01	LC/MS Neg			100020852
3-Hydroxypyridine glucuronide	Xenobiotics	Chemical	1.12 (1.04, 1.20)	3.47E-03	1.14E-01	LC/MS Pos Early			100020591
Arachidoylcarnitine (C20)*	Lipid	Fatty Acid Metabolism (Acyl Carnitine, Long Chain Saturated)	0.89 (0.82, 0.96)	3.55E-03	1.14E-01	LC/MS Pos Late	HMDB06460		100015833
S-Methylcysteine sulfoxide	Amino Acid	Methionine, Cysteine, SAM and Taurine Metabolism	0.89 (0.83, 0.96)	3.56E-03	1.14E-01	LC/MS Pos Early	HMDB29432	82142	100002927
PC (18:0/18:2)*	Lipid	Phosphatidylcholine (PC)	0.90 (0.84, 0.97)	3.75E-03	1.15E-01	LC/MS Pos Late			100008980
(2,4 or 2,5)-Dimethylphenol sulfate	Xenobiotics	Food Component/Plant	1.12 (1.04, 1.20)	4.09E-03	1.15E-01	LC/MS Neg			100020519
N-Formylanthranilic acid	Amino Acid	Tryptophan Metabolism	0.89 (0.83, 0.96)	4.09E-03	1.15E-01	LC/MS Neg	HMDB04089	101399	100004561
Octadecadienedioate (C18:2-DC)*	Lipid	Fatty Acid, Dicarboxylate	0.89 (0.83, 0.96)	4.13E-03	1.15E-01	LC/MS Neg			100019982

Syringol sulfate	Xenobiotics	Food Component/Plant	1.12 (1.04, 1.21)	4.23E-03	1.15E-01	LC/MS Neg			100006256
Docosadioate (C22-DC)	Lipid	Fatty Acid, Dicarboxylate	0.90 (0.83, 0.97)	4.77E-03	1.24E-01	LC/MS Neg		244872	100002952
PC (18:2/20:4n6)*	Lipid	Phosphatidylcholine (PC)	0.90 (0.84, 0.97)	5.02E-03	1.24E-01	LC/MS Pos Late	HMDB08147		100009131
PE (O-16:0/18:1)*	Lipid	Plasmalogen	0.90 (0.84, 0.97)	5.10E-03	1.24E-01	LC/MS Pos Late	HMDB11342		100009005
PI (18:0/18:1)*	Lipid	Phosphatidylinositol (PI)	0.90 (0.83, 0.97)	5.25E-03	1.24E-01	LC/MS Pos Late			100009181
LysoPC (24:0)	Lipid	Lysophospholipid	0.90 (0.83, 0.97)	5.48E-03	1.24E-01	LC/MS Pos Late	HMDB10405		100002873
Hippurate	Xenobiotics	Benzoate Metabolism	0.90 (0.83, 0.97)	5.86E-03	1.24E-01	LC/MS Neg	HMDB00714	464	100000014
SM (d18:2/14:0, d18:1/14:1)*	Lipid	Sphingomyelins	0.90 (0.84, 0.97)	5.89E-03	1.24E-01	LC/MS Pos Late			100005985
Androstenediol (3β,17β) disulfate (1)	Lipid	Androgenic Steroids	1.11 (1.03, 1.20)	5.99E-03	1.24E-01	LC/MS Neg	HMDB03818	87120982	100001992
PE (18:2/18:2)*	Lipid	Phosphatidylethanolamine (PE)	0.90 (0.84, 0.97)	5.99E-03	1.24E-01	LC/MS Pos Late	HMDB09093	9546812	100009217
Sphinganine-1-phosphate	Lipid	Sphingolipid Synthesis	0.91 (0.85, 0.97)	6.07E-03	1.24E-01	LC/MS Pos Late	HMDB01383	520	100001876
PI (18:0/18:2)	Lipid	Phosphatidylinositol (PI)	0.90 (0.84, 0.97)	6.18E-03	1.24E-01	LC/MS Pos Late	HMDB09809		100008994
PE (O-16:0/18:2)*	Lipid	Plasmalogen	0.90 (0.84, 0.97)	6.30E-03	1.24E-01	LC/MS Pos Late	HMDB11343		100009069
Glycerophosphoethanolamine	Lipid	Phospholipid Metabolism	0.91 (0.84, 0.97)	7.09E-03	1.34E-01	LC/MS Pos Early	HMDB00114	123874	100001620
16α-Hydroxy DHEA 3-sulfate	Lipid	Androgenic Steroids	1.11 (1.03, 1.20)	7.15E-03	1.34E-01	LC/MS Neg			100002126
SM (d18:1/24:0)	Lipid	Sphingomyelins	0.91 (0.84, 0.97)	7.25E-03	1.34E-01	LC/MS Pos Late			100006298
Undecanedioate (C11-DC)	Lipid	Fatty Acid, Dicarboxylate	0.91 (0.84, 0.97)	8.01E-03	1.41E-01	LC/MS Neg	HMDB00888	15816	100001617
Asparagine	Amino Acid	Alanine and Aspartate Metabolism	0.91 (0.84, 0.97)	8.05E-03	1.41E-01	LC/MS Pos Early	HMDB00168	6267	917
Androstenediol (3α, 17α) monosulfate (2)	Lipid	Androgenic Steroids	1.11 (1.03, 1.20)	8.15E-03	1.41E-01	LC/MS Neg			100002026
Catechol glucuronide	Amino Acid	Tyrosine Metabolism	1.10 (1.02, 1.19)	8.85E-03	1.51E-01	LC/MS Neg		75124209	100009326
Pregnenolone sulfate	Lipid	Pregnenolone Steroids	1.11 (1.03, 1.20)	9.72E-03	1.60E-01	LC/MS Neg	HMDB00774	105074	100002129
4-Guanidinobutanoate	Amino Acid	Guanidino and Acetamido Metabolism	0.91 (0.85, 0.98)	9.82E-03	1.60E-01	LC/MS Pos Early	HMDB03464	500	100000096
LysoPE (O-18:0)*	Lipid	Lysoplasmalogen	0.91 (0.84, 0.98)	9.94E-03	1.60E-01	LC/MS Pos Late			100003001
LysoPE (18:0)	Lipid	Lysophospholipid	0.91 (0.84, 0.98)	1.05E-02	1.66E-01	LC/MS Pos Late	HMDB11130	9547068	100001461
Androstenediol (3β,17β) monosulfate (1)	Lipid	Androgenic Steroids	1.11 (1.02, 1.19)	1.08E-02	1.67E-01	LC/MS Neg	HMDB03818	13847309	100002028
Sphinganine	Lipid	Sphingolipid Synthesis	0.91 (0.85, 0.98)	1.10E-02	1.67E-01	LC/MS Pos Late	HMDB00269	3126	313
β-Cryptoxanthin	Cofactors and Vitamins	Vitamin A Metabolism	0.91 (0.84, 0.98)	1.33E-02	1.94E-01	LC/MS Pos Late	HMDB33844	6384256	100001977
PE (18:1/18:2)*	Lipid	Phosphatidylethanolamine (PE)	0.91 (0.85, 0.98)	1.34E-02	1.94E-01	LC/MS Pos Late	HMDB05349	9546753	100009078
Dehydroepiandrosterone sulfate (DHEA-S)	Lipid	Androgenic Steroids	1.11 (1.02, 1.20)	1.35E-02	1.94E-01	LC/MS Neg	HMDB01032	12594	100000792
Glutamine	Amino Acid	Glutamate Metabolism	0.91 (0.85, 0.98)	1.37E-02	1.94E-01	LC/MS Pos Early	HMDB00641	5961	563
Cytidine	Nucleotide	Pyrimidine Metabolism, Cytidine containing	1.10 (1.02, 1.19)	1.44E-02	2.01E-01	LC/MS Pos Early	HMDB00089	6175	827
Linolenoylcarnitine (C18:3)*	Lipid	Fatty Acid Metabolism (Acyl Carnitine, Polyunsaturated)	0.91 (0.84, 0.98)	1.49E-02	2.06E-01	LC/MS Pos Late			100015831
Glycine	Amino Acid	Glycine, Serine and Threonine Metabolism	0.91 (0.84, 0.98)	1.59E-02	2.09E-01	LC/MS Pos Early	HMDB00123	750	340

Androstenediol (3β,17β) monosulfate (2)	Lipid	Androgenic Steroids	1.10 (1.02, 1.18)	1.61E-02	2.09E-01	LC/MS Neg			100002029
Stearoylcarnitine (C18)	Lipid	Fatty Acid Metabolism (Acyl Carnitine, Long Chain Saturated)	0.91 (0.85, 0.98)	1.62E-02	2.09E-01	LC/MS Pos Late	HMDB00848	6426855	100001391
3-Methyladipate	Lipid	Fatty Acid, Dicarboxylate	0.91 (0.84, 0.98)	1.65E-02	2.09E-01	LC/MS Polar	HMDB00555	12292	100001765
Pimelate (C7-DC)	Lipid	Fatty Acid, Dicarboxylate	0.91 (0.85, 0.98)	1.65E-02	2.09E-01	LC/MS Polar	HMDB00857	385	100000101
p-Cresol sulfate	Xenobiotics	Benzoate Metabolism	0.91 (0.85, 0.98)	1.66E-02	2.09E-01	LC/MS Neg	HMDB11635	4615423	100001315
N-Acetylhistidine	Amino Acid	Histidine Metabolism	0.91 (0.85, 0.98)	1.70E-02	2.10E-01	LC/MS Neg	HMDB32055	75619	100001293
Phenylacetylglutamate	Peptide	Acetylated Peptides	0.91 (0.84, 0.98)	1.72E-02	2.10E-01	LC/MS Neg	HMDB59772	11579826	100009045
Levulinoylcarnitine	Xenobiotics	Food Component/Plant	0.91 (0.85, 0.98)	1.74E-02	2.10E-01	LC/MS Pos Early			100021140
γ-Glutamylvaline	Peptide	Gamma-glutamyl Amino Acid	1.10 (1.02, 1.18)	1.82E-02	2.17E-01	LC/MS Pos Early	HMDB11172	7015683	100001126
SM (d18:1/16:0)	Lipid	Sphingomyelins	0.92 (0.86, 0.99)	1.91E-02	2.22E-01	LC/MS Pos Late		9939941	100002107
LysoPI (18:1)	Lipid	Lysophospholipid	0.92 (0.85, 0.99)	1.92E-02	2.22E-01	LC/MS Neg			100001777
SM (d18:2/16:0, d18:1/16:1)*	Lipid	Sphingomyelins	0.92 (0.85, 0.99)	2.00E-02	2.23E-01	LC/MS Pos Late			100004329
Campesterol	Lipid	Sterol	0.92 (0.85, 0.99)	2.02E-02	2.23E-01	LC/MS Pos Late	HMDB02869	173183	100001269
Cortolone glucuronide (1)	Lipid	Corticosteroids	1.10 (1.01, 1.18)	2.08E-02	2.23E-01	LC/MS Neg			100015971
PC (16:0/18:2)	Lipid	Phosphatidylcholine (PC)	0.92 (0.86, 0.99)	2.11E-02	2.23E-01	LC/MS Pos Late	HMDB07973	5287971	1537
LysoPC (18:0)	Lipid	Lysophospholipid	0.92 (0.85, 0.99)	2.11E-02	2.23E-01	LC/MS Pos Late	HMDB10384	497299	100001271
2-Ketocaprylate	Amino Acid	Leucine, Isoleucine and Valine Metabolism	0.91 (0.85, 0.99)	2.13E-02	2.23E-01	LC/MS Polar	HMDB13211	67600	100020833
γ-Glutamylglutamine	Peptide	Gamma-glutamyl Amino Acid	0.92 (0.85, 0.99)	2.18E-02	2.23E-01	LC/MS Pos Early	HMDB11738	150914	1140
2-LysoPE (18:0)*	Lipid	Lysophospholipid	0.92 (0.85, 0.99)	2.19E-02	2.23E-01	LC/MS Neg	HMDB11129		100003901
o-Cresol sulfate	Xenobiotics	Benzoate Metabolism	1.09 (1.01, 1.18)	2.19E-02	2.23E-01	LC/MS Neg		11615528	100001806
LysoPE (20:4n6)*	Lipid	Lysophospholipid	0.92 (0.85, 0.99)	2.20E-02	2.23E-01	LC/MS Pos Late	HMDB11517	42607465	100001571
p-Cresol glucuronide*	Amino Acid	Tyrosine Metabolism	0.92 (0.85, 0.99)	2.21E-02	2.23E-01	LC/MS Neg	HMDB11686	154035	100006191
4-Hydroxychlorothalonil	Xenobiotics	Chemical	0.91 (0.84, 0.99)	2.29E-02	2.28E-01	LC/MS Neg		34217	100006082
Glycerophosphorylcholine (GPC)	Lipid	Phospholipid Metabolism	0.92 (0.85, 0.99)	2.31E-02	2.28E-01	LC/MS Pos Early	HMDB00086	71920	100000269
Ethyl α-glucopyranoside	Xenobiotics	Food Component/Plant	0.92 (0.85, 0.99)	2.50E-02	2.44E-01	LC/MS Pos Early		428040	100015965
SM (d18:1/24:1, d18:2/24:0)*	Lipid	Sphingomyelins	0.92 (0.86, 0.99)	2.53E-02	2.44E-01	LC/MS Pos Late	HMDB12107		100005986
SM (d17:1/14:0, d16:1/15:0)*	Lipid	Sphingomyelins	0.92 (0.85, 0.99)	2.65E-02	2.51E-01	LC/MS Pos Late			100020276
SM (d18:2/24:1, d18:1/24:2)*	Lipid	Sphingomyelins	0.92 (0.86, 0.99)	2.66E-02	2.51E-01	LC/MS Pos Late			100008957
4-Acetylcatechol sulfate (2)	Xenobiotics	Food Component/Plant	1.08 (1.01, 1.16)	2.82E-02	2.63E-01	LC/MS Neg			100020854
Tryptophan betaine	Amino Acid	Tryptophan Metabolism	0.92 (0.86, 0.99)	2.95E-02	2.67E-01	LC/MS Pos Early	HMDB61115	442106	100001743
γ-Glutamylglycine	Peptide	Gamma-glutamyl Amino Acid	0.92 (0.85, 0.99)	2.96E-02	2.67E-01	LC/MS Pos Early	HMDB11667	165527	100001294
Valylglycine	Peptide	Dipeptide	0.92 (0.85, 0.99)	3.00E-02	2.67E-01	LC/MS Neg	HMDB29127	136487	100003641
Branched chain 14:0 dicarboxylic acid**	Lipid	Fatty Acid, Dicarboxylate	0.92 (0.85, 0.99)	3.02E-02	2.67E-01	LC/MS Neg			100021374
11β-Hydroxyandrosterone glucuronide	Lipid	Androgenic Steroids	1.09 (1.01, 1.18)	3.03E-02	2.67E-01	LC/MS Neg			100020541

SM (d17:2/16:0, d18:2/15:0)*	Lipid	Sphingomyelins	0.92 (0.85, 0.99)	3.07E-02	2.67E-01	LC/MS Pos Late			100015793
SM (d18:1/23:0)*	Lipid	Sphingomyelins	0.92 (0.85, 0.99)	3.10E-02	2.67E-01	LC/MS Pos Late	HMDB12105		100008955
SM (d18:1/21:0, d17:1/22:0, d16:1/23:0)*	Lipid	Sphingomyelins	0.92 (0.85, 0.99)	3.14E-02	2.67E-01	LC/MS Pos Late			100009025
Pregnenediol disulfate (C21H34O8S2)*	Lipid	Pregnenolone Steroids	1.09 (1.01, 1.18)	3.15E-02	2.67E-01	LC/MS Neg			100001993
Dodecenedioate (C12:1-DC)*	Lipid	Fatty Acid, Dicarboxylate	0.92 (0.86, 0.99)	3.19E-02	2.67E-01	LC/MS Neg			100019972
N-Acetylaspartate (NAA)	Amino Acid	Alanine and Aspartate Metabolism	0.93 (0.86, 0.99)	3.19E-02	2.67E-01	LC/MS Neg	HMDB00812	65065	100000787
Adipoylcarnitine (C6-DC)	Lipid	Fatty Acid Metabolism (Acyl Carnitine, Dicarboxylate)	1.09 (1.01, 1.17)	3.26E-02	2.69E-01	LC/MS Pos Early	HMDB61677	71296139	100006614
13-HODE + 9-HODE	Lipid	Fatty Acid, Monohydroxy	0.93 (0.86, 0.99)	3.34E-02	2.74E-01	LC/MS Neg			100002196
2-Butenoylglycine	Lipid	Fatty Acid Metabolism (Acyl Glycine)	0.92 (0.86, 0.99)	3.44E-02	2.77E-01	LC/MS Neg		6303498	100016110
Ribulonate/xylulonate/lyxonate*	Carbohydrate	Pentose Metabolism	0.92 (0.86, 0.99)	3.50E-02	2.77E-01	LC/MS Polar			100019968
4-Acetylcatechol sulfate (1)	Xenobiotics	Food Component/Plant	1.08 (1.01, 1.16)	3.52E-02	2.77E-01	LC/MS Neg			100020853
SM (d18:2/24:2)*	Lipid	Sphingomyelins	0.92 (0.86, 0.99)	3.55E-02	2.77E-01	LC/MS Pos Late			100015789
Suberate (C8-DC)	Lipid	Fatty Acid, Dicarboxylate	0.93 (0.86, 0.99)	3.60E-02	2.77E-01	LC/MS Polar	HMDB00893	10457	100000016
Choline phosphate	Lipid	Phospholipid Metabolism	0.93 (0.87, 1.00)	3.60E-02	2.77E-01	LC/MS Polar	HMDB01565	1014	267
2-Hydroxybehenate	Lipid	Fatty Acid, Monohydroxy	0.93 (0.86, 1.00)	3.61E-02	2.77E-01	LC/MS Neg		193484	100015637
PC (16:0/18:0)	Lipid	Phosphatidylcholine (PC)	0.93 (0.86, 1.00)	3.68E-02	2.78E-01	LC/MS Pos Late	HMDB07970		100008921
Linoleoylcarnitine (C18:2)*	Lipid	Fatty Acid Metabolism (Acyl Carnitine, Polyunsaturated)	0.92 (0.85, 0.99)	3.68E-02	2.78E-01	LC/MS Pos Late	HMDB06469	6450015	100003151
SM (d18:2/21:0, d16:2/23:0)*	Lipid	Sphingomyelins	0.92 (0.86, 1.00)	3.76E-02	2.82E-01	LC/MS Pos Late			100015790
1,7-Dimethylurate	Xenobiotics	Xanthine Metabolism	0.92 (0.86, 1.00)	3.90E-02	2.89E-01	LC/MS Neg	HMDB11103	91611	100001399
LysoPE (O-16:0)*	Lipid	Lysoplasmalogen	0.93 (0.86, 1.00)	4.04E-02	2.96E-01	LC/MS Pos Late			100003000
PC (O-16:0/16:0)*	Lipid	Plasmalogen	0.93 (0.86, 1.00)	4.07E-02	2.96E-01	LC/MS Pos Late	HMDB11206	11146967	100009162
N-Acetylglycine	Amino Acid	Glycine, Serine and Threonine Metabolism	0.93 (0.86, 1.00)	4.09E-02	2.96E-01	LC/MS Pos Early	HMDB00532	10972	100001006
N-Acetylglutamate	Amino Acid	Glutamate Metabolism	0.92 (0.86, 1.00)	4.15E-02	2.97E-01	LC/MS Pos Early	HMDB01138	70914	100000282
Histidine	Amino Acid	Histidine Metabolism	0.93 (0.86, 1.00)	4.25E-02	2.99E-01	LC/MS Neg	HMDB00177	6274	355
Glycosyl ceramide (d18:2/22:0)*	Lipid	Hexosylceramides (HCER)	0.93 (0.86, 1.00)	4.28E-02	2.99E-01	LC/MS Pos Late			100015625
Cortisone	Lipid	Corticosteroids	1.08 (1.00, 1.17)	4.29E-02	2.99E-01	LC/MS Neg	HMDB02802	222786	273
LysoPG (18:1)*	Lipid	Lysophospholipid	0.93 (0.86, 1.00)	4.34E-02	2.99E-01	LC/MS Neg			100005716
5α-Androstan-3β,17β-diol disulfate	Lipid	Androgenic Steroids	1.08 (1.00, 1.16)	4.35E-02	2.99E-01	LC/MS Neg	HMDB00493	242332	100001987
Glycosyl ceramide (d18:2/24:1, d18:1/24:2)*	Lipid	Hexosylceramides (HCER)	0.93 (0.86, 1.00)	4.41E-02	2.99E-01	LC/MS Pos Late			100015745
SM (d18:1/22:0)*	Lipid	Sphingomyelins	0.93 (0.86, 1.00)	4.42E-02	2.99E-01	LC/MS Pos Late	HMDB12103		100006294
DihydroSM (d18:0/16:0)*	Lipid	Dihydrosphingomyelins	0.93 (0.86, 1.00)	4.57E-02	3.05E-01	LC/MS Pos Late		9939965	100008954
PE (O-18:0/20:4)*	Lipid	Plasmalogen	0.93 (0.86, 1.00)	4.59E-02	3.05E-01	LC/MS Pos Late	HMDB05779	9547058	100008999
Glycolithocholate	Lipid	Secondary Bile Acid Metabolism	0.93 (0.86, 1.00)	4.60E-02	3.05E-01	LC/MS Neg	HMDB00698	115245	100001064
3-Hydroxy-2-methylpyridine sulfate	Xenobiotics	Chemical	1.08 (1.00, 1.16)	4.77E-02	3.12E-01	LC/MS Neg			100020975

3β-Hydroxy-5-cholenoic acid	Lipid	Secondary Bile Acid Metabolism	0.93 (0.86, 1.00)	4.78E-02	3.12E-01	LC/MS Neg	HMDB00308	92997	100004182
DihydroSM (d18:0/18:0, d19:0/17:0)*	Lipid	Dihydrosphingomyelins	1.08 (1.00, 1.17)	4.83E-02	3.13E-01	LC/MS Pos Late	HMDB12087		100009027
3-Methoxycatechol sulfate (1)	Xenobiotics	Benzoate Metabolism	1.08 (1.00, 1.16)	4.96E-02	3.18E-01	LC/MS Neg			100006375
Arachidate (20:0)	Lipid	Long Chain Saturated Fatty Acid	0.93 (0.86, 1.00)	4.98E-02	3.18E-01	LC/MS Neg	HMDB02212	10467	893
Hypotaurine	Amino Acid	Methionine, Cysteine, SAM and Taurine Metabolism	0.93 (0.87, 1.00)	5.01E-02	3.18E-01	LC/MS Polar	HMDB00965	107812	358
SM (d18:1/14:0, d16:1/16:0)*	Lipid	Sphingomyelins	0.93 (0.86, 1.00)	5.10E-02	3.21E-01	LC/MS Pos Late	HMDB12097	11433862	100004328
5α-Pregnan-diol disulfate	Lipid	Progestin Steroids	1.09 (1.00, 1.18)	5.32E-02	3.30E-01	LC/MS Neg		5127902	100002015
Glycodeoxycholate	Lipid	Secondary Bile Acid Metabolism	0.93 (0.86, 1.00)	5.34E-02	3.30E-01	LC/MS Neg	HMDB00631	3035026	100000436
LysoPA (18:2)*	Lipid	Lysophospholipid	0.93 (0.86, 1.00)	5.36E-02	3.30E-01	LC/MS Neg	HMDB07856		100009082
2-Methoxyacetaminophen glucuronide*	Xenobiotics	Drug - Analgesics, Anesthetics	0.93 (0.86, 1.00)	5.51E-02	3.36E-01	LC/MS Neg		14367271	100001795
LysoPE (16:0)	Lipid	Lysophospholipid	0.93 (0.86, 1.00)	5.57E-02	3.36E-01	LC/MS Pos Late	HMDB11503	9547069	100001567
3-Hydroxybutyroylglycine**	Lipid	Fatty Acid Metabolism (Acyl Glycine)	0.93 (0.86, 1.00)	5.58E-02	3.36E-01	LC/MS Pos Early			100020211
Gluconate	Xenobiotics	Food Component/Plant	0.93 (0.86, 1.00)	5.62E-02	3.37E-01	LC/MS Polar	HMDB00625	10690	338
Hydroquinone β-D-glucopyranoside	Xenobiotics	Food Component/Plant	1.07 (1.00, 1.16)	5.72E-02	3.40E-01	LC/MS Neg	HMDB29943	346	100002220
Ergothioneine	Xenobiotics	Food Component/Plant	0.93 (0.86, 1.00)	5.78E-02	3.41E-01	LC/MS Pos Early	HMDB03045	3032311	100002154
3-Hydroxytridecanoate	Lipid	Fatty Acid, Monohydroxy	0.93 (0.85, 1.00)	5.87E-02	3.44E-01	LC/MS Neg	HMDB61655	5312749	100021388
N-(2-Furoyl)glycine	Xenobiotics	Food Component/Plant	1.07 (1.00, 1.15)	5.90E-02	3.44E-01	LC/MS Neg	HMDB00439	21863	100001086
Guanidinoacetate	Amino Acid	Creatine Metabolism	0.93 (0.86, 1.00)	6.16E-02	3.56E-01	LC/MS Polar	HMDB00128	763	344
Taurine	Amino Acid	Methionine, Cysteine, SAM and Taurine Metabolism	0.93 (0.87, 1.00)	6.19E-02	3.56E-01	LC/MS Pos Early	HMDB00251	1123	512
Hydrochlorothiazide	Xenobiotics	Drug - Cardiovascular	1.07 (1.00, 1.15)	6.39E-02	3.64E-01	LC/MS Neg	HMDB01928	3639	100002734
Androstenediol (3α, 17α) monosulfate (3)	Lipid	Androgenic Steroids	1.08 (1.00, 1.17)	6.42E-02	3.64E-01	LC/MS Neg			100002027
2-Methoxyresorcinol sulfate	Xenobiotics	Chemical	1.07 (1.00, 1.15)	6.50E-02	3.66E-01	LC/MS Neg			100006184
3-Methoxytyramine sulfate	Amino Acid	Tyrosine Metabolism	0.93 (0.86, 1.00)	6.64E-02	3.66E-01	LC/MS Neg			100004634
Pregnenediol sulfate (C21H34O5S)*	Lipid	Pregnenolone Steroids	1.08 (0.99, 1.17)	6.65E-02	3.66E-01	LC/MS Neg			100002067
6-Hydroxyindole sulfate	Xenobiotics	Chemical	0.93 (0.86, 1.00)	6.69E-02	3.66E-01	LC/MS Neg		122198196	100006260
PC (P-16:0/16:1)*	Lipid	Plasmalogen	0.94 (0.87, 1.00)	6.71E-02	3.66E-01	LC/MS Pos Late	HMDB11207		100009160
PC (18:0/18:1)	Lipid	Phosphatidylcholine (PC)	0.93 (0.86, 1.01)	6.77E-02	3.66E-01	LC/MS Pos Late	HMDB08038		100008904
3-Indoxyl sulfate	Amino Acid	Tryptophan Metabolism	0.93 (0.87, 1.01)	6.84E-02	3.66E-01	LC/MS Neg	HMDB00682	10258	100000467
S-Methylcysteine	Amino Acid	Methionine, Cysteine, SAM and Taurine Metabolism	0.93 (0.86, 1.01)	6.85E-02	3.66E-01	LC/MS Neg	HMDB02108	24417	100002749
PE (18:0/18:1)	Lipid	Phosphatidylethanolamine (PE)	0.94 (0.87, 1.01)	6.91E-02	3.66E-01	LC/MS Pos Late	HMDB08993		100001856
N-Oleoyltaurine	Lipid	Endocannabinoid	0.93 (0.87, 1.01)	6.92E-02	3.66E-01	LC/MS Neg		6437033	100003119
Urea	Amino Acid	Urea cycle; Arginine and Proline Metabolism	0.93 (0.86, 1.01)	6.93E-02	3.66E-01	LC/MS Pos Early	HMDB00294	1176	533
4-Hydroxyglutamate	Amino Acid	Glutamate Metabolism	1.07 (0.99, 1.16)	6.95E-02	3.66E-01	LC/MS Pos Early	HMDB01344	439902	100002544

Indolin-2-one	Xenobiotics	Food Component/Plant	0.94 (0.87, 1.01)	7.00E-02	3.66E-01	LC/MS Pos Early		321710	100004318
LysoPG (18:2)*	Lipid	Lysophospholipid	0.93 (0.87, 1.01)	7.13E-02	3.70E-01	LC/MS Neg			100009227
N-Palmitoyl-sphinganine (d18:0/16:0)	Lipid	Dihydroceramides	0.93 (0.87, 1.01)	7.17E-02	3.70E-01	LC/MS Pos Late	HMDB11760	5283572	100009028
1-Methylxanthine	Xenobiotics	Xanthine Metabolism	0.93 (0.86, 1.01)	7.26E-02	3.73E-01	LC/MS Neg	HMDB10738	80220	100001405
5α-Androstan-3α,17α-diol monosulfate	Lipid	Androgenic Steroids	1.07 (0.99, 1.15)	7.56E-02	3.85E-01	LC/MS Neg			100002008
PE (0-16:0/20:4)*	Lipid	Plasmalogen	0.94 (0.87, 1.01)	7.59E-02	3.85E-01	LC/MS Pos Late	HMDB11352		100009002
PE (18:1/20:4)*	Lipid	Phosphatidylethanolamine (PE)	0.94 (0.87, 1.01)	7.67E-02	3.85E-01	LC/MS Pos Late	HMDB09069		100009219
3-Formylindole	Xenobiotics	Food Component/Plant	1.07 (0.99, 1.16)	7.68E-02	3.85E-01	LC/MS Polar	HMDB29737	10256	100020219
Cholesterol	Lipid	Sterol	0.93 (0.87, 1.01)	7.75E-02	3.87E-01	LC/MS Pos Late	HMDB00067	11025495	266
2-Methoxyacetaminophen sulfate*	Xenobiotics	Drug - Analgesics, Anesthetics	0.94 (0.87, 1.01)	7.95E-02	3.92E-01	LC/MS Neg		86290014	100001793
Maleate	Lipid	Fatty Acid, Dicarboxylate	1.07 (0.99, 1.15)	7.96E-02	3.92E-01	LC/MS Polar	HMDB00176	444266	100000707
Citrulline	Amino Acid	Urea cycle; Arginine and Proline Metabolism	0.93 (0.86, 1.01)	8.03E-02	3.94E-01	LC/MS Pos Early	HMDB00904	9750	391
3-Carboxy-4-methyl-5-pentyl-2-furanpropionate (3-CMPFP)**	Lipid	Fatty Acid, Dicarboxylate	0.93 (0.87, 1.01)	8.09E-02	3.95E-01	LC/MS Neg		194501	100020004
N,N-Dimethyl-5-aminovalerate	Amino Acid	Lysine Metabolism	0.94 (0.87, 1.01)	8.21E-02	3.98E-01	LC/MS Pos Early			100020842
Ethyl β-glucopyranoside	Xenobiotics	Food Component/Plant	0.93 (0.86, 1.01)	8.28E-02	3.99E-01	LC/MS Neg		121667	100020377
Cinnamoylglycine	Xenobiotics	Food Component/Plant	0.94 (0.87, 1.01)	8.32E-02	3.99E-01	LC/MS Neg	HMDB11621	709625	100002253
2,3-Dihydroxypyridine	Xenobiotics	Food Component/Plant	1.07 (0.99, 1.15)	8.41E-02	3.99E-01	LC/MS Pos Early		28115	100000861
Threonine	Amino Acid	Glycine, Serine and Threonine Metabolism	0.94 (0.87, 1.01)	8.41E-02	3.99E-01	LC/MS Pos Early	HMDB00167	6288	564
Octadecenedioate (C18:1-DC)	Lipid	Fatty Acid, Dicarboxylate	0.94 (0.87, 1.01)	8.65E-02	4.06E-01	LC/MS Neg			100019978
S-Methylmethionine	Amino Acid	Methionine, Cysteine, SAM and Taurine Metabolism	0.94 (0.87, 1.01)	8.66E-02	4.06E-01	LC/MS Pos Early	HMDB38670	458	100002183
Theanine	Xenobiotics	Food Component/Plant	1.07 (0.99, 1.15)	8.81E-02	4.11E-01	LC/MS Neg	HMDB34365	439378	100000784
2-Hydroxyarachidate*	Lipid	Fatty Acid, Monohydroxy	0.94 (0.88, 1.01)	8.96E-02	4.16E-01	LC/MS Neg		5225199	100019892
Ibuprofen	Xenobiotics	Drug - Analgesics, Anesthetics	0.94 (0.88, 1.01)	9.07E-02	4.19E-01	LC/MS Neg	HMDB01925	3672	1487
2-Aminoadipate	Amino Acid	Lysine Metabolism	1.07 (0.99, 1.15)	9.11E-02	4.19E-01	LC/MS Polar	HMDB00510	469	381
9,10-DiHOME	Lipid	Fatty Acid, Dihydroxy	0.94 (0.87, 1.01)	9.26E-02	4.22E-01	LC/MS Neg	HMDB04704	9966640	179
LysoPE (O-18:1)*	Lipid	Lysoplasmalogen	0.94 (0.87, 1.01)	9.34E-02	4.22E-01	LC/MS Pos Late			100005372
Sphingosine 1-phosphate	Lipid	Sphingosines	0.94 (0.88, 1.01)	9.34E-02	4.22E-01	LC/MS Pos Late	HMDB00277	5283560	100000626
Citraconate/glutaconate	Energy	TCA Cycle	1.07 (0.99, 1.15)	9.55E-02	4.23E-01	LC/MS Polar			100006438
Pregnenetriol disulfate*	Lipid	Pregnenolone Steroids	1.07 (0.99, 1.15)	9.59E-02	4.23E-01	LC/MS Neg			100021104
Linolenate [α or γ; (18:3n3 or 6)]	Lipid	Long Chain Polyunsaturated Fatty Acid (n3 and n6)	0.94 (0.87, 1.01)	9.59E-02	4.23E-01	LC/MS Neg	HMDB03073	5280934	100001337
Hydroxy-CMPF*	Lipid	Fatty Acid, Dicarboxylate	0.94 (0.87, 1.01)	9.66E-02	4.23E-01	LC/MS Neg			100019794
N-Palmitoyl-sphingosine (d18:1/16:0)	Lipid	Ceramides	0.94 (0.87, 1.01)	9.67E-02	4.23E-01	LC/MS Pos Late	HMDB04949	5283564	1518
Glycolithocholate sulfate*	Lipid	Secondary Bile Acid Metabolism	0.94 (0.87, 1.01)	9.70E-02	4.23E-01	LC/MS Neg	HMDB02639	72222	100001657
Oleoyl ethanolamide	Lipid	Endocannabinoid	0.94 (0.87, 1.01)	9.74E-02	4.23E-01	LC/MS Neg	HMDB02088	5283454	1137

Nonanoylcarnitine (C9)	Lipid	Fatty Acid Metabolism (Acyl Carnitine, Medium Chain)	1.06 (0.99, 1.14)	9.79E-02	4.23E-01	LC/MS Pos Late	HMDB13288		100006620
Dihomo-linoleoylcarnitine (C20:2)*	Lipid	Fatty Acid Metabolism (Acyl Carnitine, Polyunsaturated)	0.94 (0.87, 1.01)	9.81E-02	4.23E-01	LC/MS Pos Late			100015839
Eicosenedioate (C20:1-DC)*	Lipid	Fatty Acid, Dicarboxylate	0.94 (0.87, 1.01)	9.88E-02	4.23E-01	LC/MS Neg			100021105
3-Acetylphenol sulfate	Xenobiotics	Chemical	1.06 (0.99, 1.15)	9.88E-02	4.23E-01	LC/MS Neg			100004326
2-Hydroxyacetaminophen sulfate*	Xenobiotics	Drug - Analgesics, Anesthetics	0.94 (0.87, 1.01)	1.03E-01	4.32E-01	LC/MS Neg		86290013	100001791
Glycocholate glucuronide (1)	Lipid	Primary Bile Acid Metabolism	0.94 (0.87, 1.01)	1.03E-01	4.32E-01	LC/MS Neg			100009259
LysoPI (18:2)*	Lipid	Lysophospholipid	0.94 (0.87, 1.01)	1.03E-01	4.32E-01	LC/MS Neg			100001778
SM (d18:2/23:1)*	Lipid	Sphingomyelins	0.94 (0.87, 1.01)	1.03E-01	4.32E-01	LC/MS Pos Late			100015791
Oxalate (ethanedioate)	Cofactors and Vitamins	Ascorbate and Aldarate Metabolism	0.94 (0.87, 1.01)	1.04E-01	4.32E-01	LC/MS Neg	HMDB02329	971	100000841
Linoleoyl ethanolamide	Lipid	Endocannabinoid	0.94 (0.88, 1.01)	1.06E-01	4.36E-01	LC/MS Neg	HMDB12252	5283446	100006726
Succinate	Energy	TCA Cycle	0.94 (0.88, 1.01)	1.06E-01	4.36E-01	LC/MS Neg	HMDB00254	1110	252
SM (d17:1/16:0, d18:1/15:0, d16:1/17:0)*	Lipid	Sphingomyelins	0.94 (0.87, 1.01)	1.06E-01	4.36E-01	LC/MS Pos Late			100006314
1-Methylnicotinamide	Cofactors and Vitamins	Nicotinate and Nicotinamide Metabolism	0.94 (0.87, 1.01)	1.07E-01	4.36E-01	LC/MS Pos Early	HMDB00699	457	55
3-Methoxycatechol sulfate (2)	Xenobiotics	Benzoate Metabolism	1.06 (0.99, 1.14)	1.07E-01	4.36E-01	LC/MS Neg			100006376
Sucralose	Xenobiotics	Food Component/Plant	1.06 (0.99, 1.14)	1.10E-01	4.45E-01	LC/MS Neg	HMDB31554	71485	100001789
PS (18:0/20:4)	Lipid	Phosphatidylserine (PS)	0.94 (0.87, 1.01)	1.11E-01	4.47E-01	LC/MS Pos Late	HMDB12383		100001872
PE (18:0/18:2)*	Lipid	Phosphatidylethanolamine (PE)	0.94 (0.87, 1.01)	1.12E-01	4.51E-01	LC/MS Pos Late	HMDB08994	9546749	100008976
4-Acetaminophen sulfate	Xenobiotics	Drug - Analgesics, Anesthetics	0.94 (0.87, 1.01)	1.14E-01	4.57E-01	LC/MS Neg	HMDB59911	83939	2049
Glycoursodeoxycholate	Lipid	Secondary Bile Acid Metabolism	0.94 (0.87, 1.01)	1.15E-01	4.58E-01	LC/MS Neg	HMDB00708	12310288	100002911
3-Methyl catechol sulfate (1)	Xenobiotics	Benzoate Metabolism	1.06 (0.99, 1.14)	1.16E-01	4.60E-01	LC/MS Neg			100004112
Glyco-β-muricholate**	Lipid	Primary Bile Acid Metabolism	0.94 (0.87, 1.02)	1.17E-01	4.60E-01	LC/MS Neg			100020214
N-Acetyltyrosine	Amino Acid	Tyrosine Metabolism	1.06 (0.99, 1.13)	1.17E-01	4.61E-01	LC/MS Neg	HMDB00866	68310	100001104
Vanillylmandelate (VMA)	Amino Acid	Tyrosine Metabolism	0.94 (0.87, 1.02)	1.18E-01	4.61E-01	LC/MS Neg	HMDB00291	1245	1111
Propionylglycine	Lipid	Fatty Acid Metabolism (also BCAA Metabolism)	0.94 (0.88, 1.02)	1.20E-01	4.67E-01	LC/MS Polar	HMDB00783	98681	100001150
Phosphoethanolamine	Lipid	Phospholipid Metabolism	0.95 (0.88, 1.01)	1.20E-01	4.67E-01	LC/MS Pos Early	HMDB00224	1015	1026
Pregnenetriol sulfate*	Lipid	Pregnenolone Steroids	1.06 (0.98, 1.15)	1.22E-01	4.72E-01	LC/MS Neg			100021103
PI (18:0/20:4)	Lipid	Phosphatidylinositol (PI)	0.94 (0.88, 1.02)	1.24E-01	4.76E-01	LC/MS Pos Late	HMDB09815		100000616
Aconitate [cis or trans]	Energy	TCA Cycle	0.94 (0.88, 1.02)	1.25E-01	4.77E-01	LC/MS Neg			100001359
3-(N-Acetyl-L-cystein-S-yl) acetaminophen	Xenobiotics	Drug - Analgesics, Anesthetics	0.94 (0.88, 1.02)	1.26E-01	4.77E-01	LC/MS Neg		83967	2048
N-Acetyl-aspartyl-glutamate (NAAG)	Amino Acid	Glutamate Metabolism	0.94 (0.87, 1.02)	1.26E-01	4.77E-01	LC/MS Pos Early	HMDB01067	5255	100001612
Paraxanthine	Xenobiotics	Xanthine Metabolism	0.94 (0.87, 1.02)	1.27E-01	4.78E-01	LC/MS Neg	HMDB01860	4687	100000453
Eicosanedioate (C20-DC)	Lipid	Fatty Acid, Dicarboxylate	0.94 (0.87, 1.02)	1.28E-01	4.79E-01	LC/MS Neg		75502	100002951
Malate	Energy	TCA Cycle	0.94 (0.88, 1.02)	1.28E-01	4.79E-01	LC/MS Neg	HMDB00156	525	409
Hydroxyasparagine**	Amino Acid	Alanine and Aspartate Metabolism	1.07 (0.98, 1.16)	1.29E-01	4.79E-01	LC/MS Pos Early	HMDB32332	97663	100020205

2,4-Di-tert-butylphenol	Xenobiotics	Chemical	0.94 (0.88, 1.02)	1.30E-01	4.81E-01	LC/MS Neg			100021918
6-Bromotryptophan	Amino Acid	Tryptophan Metabolism	1.06 (0.98, 1.15)	1.33E-01	4.91E-01	LC/MS Neg			100020414
Lignoceroylcarnitine (C24)*	Lipid	Fatty Acid Metabolism (Acyl Carnitine, Long Chain Saturated)	0.94 (0.87, 1.02)	1.35E-01	4.95E-01	LC/MS Pos Late			100015834
Carotene diol (1)	Cofactors and Vitamins	Vitamin A Metabolism	0.94 (0.88, 1.02)	1.37E-01	5.02E-01	LC/MS Pos Late			100015966
5α-Androstan-3α,17β-diol monosulfate (1)	Lipid	Androgenic Steroids	1.06 (0.98, 1.14)	1.38E-01	5.03E-01	LC/MS Neg			100002018
SM (d18:1/20:0, d16:1/22:0)*	Lipid	Sphingomyelins	0.95 (0.88, 1.02)	1.39E-01	5.03E-01	LC/MS Pos Late	HMDB12102		100006290
5α-Pregnan-3β,20β-diol monosulfate (1)	Lipid	Progestin Steroids	1.07 (0.98, 1.16)	1.40E-01	5.05E-01	LC/MS Neg			100002009
Androsterone sulfate	Lipid	Androgenic Steroids	1.06 (0.98, 1.14)	1.42E-01	5.09E-01	LC/MS Neg	HMDB02759	159663	100001073
3-Hydroxy-3-methylglutarate	Lipid	Mevalonate Metabolism	0.94 (0.88, 1.02)	1.42E-01	5.09E-01	LC/MS Polar	HMDB00355	1662	112
PC (16:0/18:1)	Lipid	Phosphatidylcholine (PC)	0.95 (0.88, 1.02)	1.46E-01	5.22E-01	LC/MS Pos Late	HMDB07972	6436017	1539
PC (16:0/20:3n3 or 6)*	Lipid	Phosphatidylcholine (PC)	0.95 (0.88, 1.02)	1.47E-01	5.24E-01	LC/MS Pos Late			100008985
Oleoylcarnitine (C18:1)	Lipid	Fatty Acid Metabolism (Acyl Carnitine, Monounsaturated)	0.94 (0.87, 1.02)	1.48E-01	5.25E-01	LC/MS Pos Late	HMDB05065	6441392	100001501
Arginine	Amino Acid	Urea cycle; Arginine and Proline Metabolism	0.95 (0.88, 1.02)	1.50E-01	5.27E-01	LC/MS Pos Early	HMDB00517	232	231
4-Hydroxycoumarin	Xenobiotics	Drug - Cardiovascular	0.95 (0.88, 1.02)	1.51E-01	5.27E-01	LC/MS Neg		54682930	100002049
SM (d18:1/20:1, d18:2/20:0)*	Lipid	Sphingomyelins	0.95 (0.88, 1.02)	1.51E-01	5.27E-01	LC/MS Pos Late			100006292
Methionine sulfoxide	Amino Acid	Methionine, Cysteine, SAM and Taurine Metabolism	0.95 (0.88, 1.02)	1.52E-01	5.27E-01	LC/MS Pos Early	HMDB02005	158980	100000039
Glycerol 3-phosphate	Lipid	Glycerolipid Metabolism	1.06 (0.98, 1.14)	1.52E-01	5.27E-01	LC/MS Pos Early	HMDB00126	754	100000258
5α-Androstan-3β,17β-diol monosulfate (2)	Lipid	Androgenic Steroids	1.06 (0.98, 1.14)	1.52E-01	5.27E-01	LC/MS Neg			100002024
4-Acetamidophenylglucuronide	Xenobiotics	Drug - Analgesics, Anesthetics	0.95 (0.88, 1.02)	1.54E-01	5.32E-01	LC/MS Neg	HMDB10316	83944	100000043
4-Hydroxyphenylacetylglutamine	Peptide	Acetylated Peptides	0.94 (0.87, 1.02)	1.61E-01	5.51E-01	LC/MS Pos Early			100010850
Carotene diol (2)	Cofactors and Vitamins	Vitamin A Metabolism	0.95 (0.88, 1.02)	1.64E-01	5.59E-01	LC/MS Pos Late			100015967
3-(Methylthio)acetaminophen sulfate*	Xenobiotics	Drug - Analgesics, Anesthetics	0.95 (0.88, 1.02)	1.65E-01	5.59E-01	LC/MS Neg			100020827
Allantoin	Nucleotide	Purine Metabolism, (Hypo)Xanthine/Inosine containing	0.95 (0.88, 1.02)	1.66E-01	5.59E-01	LC/MS Polar	HMDB00462	204	1002
Glycerate	Carbohydrate	Glycolysis, Gluconeogenesis, and Pyruvate Metabolism	0.95 (0.88, 1.02)	1.66E-01	5.59E-01	LC/MS Polar	HMDB00139	752	1052
Maltol sulfate	Xenobiotics	Food Component/Plant	1.05 (0.98, 1.13)	1.66E-01	5.59E-01	LC/MS Neg			100006106
3β-Dydroxy-5-cholestenoate	Lipid	Sterol	0.95 (0.88, 1.02)	1.67E-01	5.59E-01	LC/MS Neg		165511	100006370
3-(3-Hydroxyphenyl)propionate	Xenobiotics	Benzoate Metabolism	0.95 (0.88, 1.02)	1.67E-01	5.59E-01	LC/MS Neg	HMDB00375	91	100001624
Nonadecanoate (19:0)	Lipid	Long Chain Saturated Fatty Acid	0.95 (0.88, 1.02)	1.68E-01	5.59E-01	LC/MS Neg	HMDB00772	12591	892
Erucate (22:1n9)	Lipid	Long Chain Monounsaturated Fatty Acid	0.95 (0.88, 1.02)	1.70E-01	5.62E-01	LC/MS Neg	HMDB02068	5281116	1087
Formiminoglutamate	Amino Acid	Histidine Metabolism	1.05 (0.98, 1.14)	1.71E-01	5.66E-01	LC/MS Pos Early	HMDB00854	439233	100002500
N6-Carbamoylthreonyladenosine	Nucleotide	Purine Metabolism, Adenine containing	1.05 (0.98, 1.14)	1.73E-01	5.70E-01	LC/MS Neg	HMDB41623	161466	100001415

Ethyl glucuronide	Xenobiotics	Chemical	0.95 (0.88, 1.02)	1.76E-01	5.76E-01	LC/MS Neg	HMDB10325	152226	100002849
Threonate	Cofactors and Vitamins	Ascorbate and Aldarate Metabolism	0.95 (0.88, 1.02)	1.77E-01	5.77E-01	LC/MS Polar	HMDB00943	151152	100001022
N-Acetylneuraminate	Carbohydrate	Nicotinate and Nicotinamide Metabolism	0.95 (0.88, 1.02)	1.79E-01	5.79E-01	LC/MS Pos Early	HMDB00230	439197	1162
γ-Glutamylcitrulline*	Peptide	Gamma-glutamyl Amino Acid	0.95 (0.88, 1.02)	1.79E-01	5.79E-01	LC/MS Pos Early			100020241
SM (d18:1/16:0(OH))**	Lipid	Sphingomyelins	0.95 (0.88, 1.02)	1.80E-01	5.79E-01	LC/MS Pos Late			100021000
Epiandrosterone sulfate	Lipid	Androgenic Steroids	1.05 (0.98, 1.14)	1.81E-01	5.79E-01	LC/MS Neg		9929317	100001287
2-Naphthol sulfate	Xenobiotics	Chemical	1.05 (0.98, 1.14)	1.81E-01	5.79E-01	LC/MS Neg		74428	100020515
Glycosyl ceramide (d18:1/20:0, d16:1/22:0)*	Lipid	Hexosylceramides (HCER)	0.95 (0.88, 1.02)	1.81E-01	5.79E-01	LC/MS Pos Late			100015882
Eicosapentaenoate (EPA; 20:5n3)	Lipid	Long Chain Polyunsaturated Fatty Acid (n3 and n6)	0.95 (0.88, 1.02)	1.83E-01	5.82E-01	LC/MS Neg	HMDB01999	446284	2050
PE (16:0/18:2)	Lipid	Phosphatidylethanolamine (PE)	0.95 (0.88, 1.03)	1.87E-01	5.91E-01	LC/MS Pos Late	HMDB05322	9546747	100001870
Dihydroorotate	Nucleotide	Pyrimidine Metabolism, Orotate containing	1.05 (0.98, 1.13)	1.87E-01	5.91E-01	LC/MS Polar	HMDB03349	648	923
Sebacate (C10-DC)	Lipid	Fatty Acid, Dicarboxylate	0.95 (0.88, 1.02)	1.89E-01	5.95E-01	LC/MS Polar	HMDB00792	5192	100001211
4-Methylguaiacol sulfate	Xenobiotics	Benzoate Metabolism	1.05 (0.98, 1.13)	1.90E-01	5.95E-01	LC/MS Neg			100006105
Ascorbic acid 2-sulfate	Cofactors and Vitamins	Ascorbate and Aldarate Metabolism	0.95 (0.88, 1.03)	1.91E-01	5.95E-01	LC/MS Polar		86484	100009329
cis-3,4-Methyleneheptanoylglycine	Lipid	Fatty Acid Metabolism (Acyl Glycine)	0.95 (0.88, 1.03)	1.92E-01	5.96E-01	LC/MS Neg			100021992
Deoxycholate	Lipid	Secondary Bile Acid Metabolism	0.95 (0.89, 1.02)	1.94E-01	6.00E-01	LC/MS Pos Late	HMDB00626	222528	302
Linoleate (18:2n6)	Lipid	Long Chain Polyunsaturated Fatty Acid (n3 and n6)	0.95 (0.89, 1.03)	1.94E-01	6.00E-01	LC/MS Neg	HMDB00673	5280450	180
Hydantoin-5-propionate	Amino Acid	Histidine Metabolism	1.05 (0.98, 1.13)	1.95E-01	6.00E-01	LC/MS Polar	HMDB01212	782	100002514
Cerotoylcarnitine (C26)*	Lipid	Fatty Acid Metabolism (Acyl Carnitine, Long Chain Saturated)	0.95 (0.88, 1.03)	1.96E-01	6.01E-01	LC/MS Pos Late	HMDB06347		100015835
Thyroxine	Amino Acid	Tyrosine Metabolism	1.05 (0.97, 1.14)	1.96E-01	6.01E-01	LC/MS Neg	HMDB01918	5819	1094
Galactonate	Carbohydrate	Fructose, Mannose and Galactose Metabolism	0.95 (0.88, 1.03)	1.98E-01	6.02E-01	LC/MS Polar	HMDB00565	128869	100001026
Dihomo-linolenoylcarnitine (C20:3n3 or 6)*	Lipid	Fatty Acid Metabolism (Acyl Carnitine, Polyunsaturated)	0.95 (0.88, 1.03)	1.99E-01	6.02E-01	LC/MS Pos Late			100015840
Pregnanolone/alloPregnanolone sulfate	Lipid	Progestin Steroids	1.06 (0.97, 1.15)	2.00E-01	6.02E-01	LC/MS Neg			100006173
Nicotinamide	Cofactors and Vitamins	Nicotinate and Nicotinamide Metabolism	0.96 (0.89, 1.02)	2.02E-01	6.02E-01	LC/MS Pos Early	HMDB01406	936	432
Taurodeoxycholate	Lipid	Secondary Bile Acid Metabolism	0.95 (0.88, 1.03)	2.02E-01	6.02E-01	LC/MS Neg	HMDB00896	2733768	1668
Methionine	Amino Acid	Methionine, Cysteine, SAM and Taurine Metabolism	0.95 (0.88, 1.03)	2.03E-01	6.02E-01	LC/MS Pos Early	HMDB00696	6137	415
Arachidonoylcarnitine (C20:4)	Lipid	Fatty Acid Metabolism (Acyl Carnitine, Polyunsaturated)	0.95 (0.88, 1.03)	2.04E-01	6.02E-01	LC/MS Pos Late			100015837
2-Aminooctanoate	Lipid	Fatty Acid, Amino	0.95 (0.88, 1.03)	2.04E-01	6.02E-01	LC/MS Pos Late	HMDB00991	69522	100004227
β-Citrylglutamate	Amino Acid	Glutamate Metabolism	0.96 (0.89, 1.02)	2.04E-01	6.02E-01	LC/MS Neg		72715786	100003271
γ-Glutamylmethionine	Peptide	Gamma-glutamyl Amino Acid	0.95 (0.88, 1.03)	2.04E-01	6.02E-01	LC/MS Pos Early	HMDB29155	7009567	100001313
4-Hydroxyhippurate	Xenobiotics	Benzoate Metabolism	0.95 (0.88, 1.03)	2.05E-01	6.02E-01	LC/MS Neg	HMDB13678	151012	100001423

2-Hydroxynervonate*	Lipid	Fatty Acid, Monohydroxy	0.95 (0.89, 1.03)	2.08E-01	6.10E-01	LC/MS Neg		5312783	100015638
3β,7β-Dihydroxy-5-cholestenoate	Lipid	Sterol	1.05 (0.97, 1.14)	2.10E-01	6.10E-01	LC/MS Neg			100001774
4-Ethylcatechol sulfate	Xenobiotics	Benzoate Metabolism	1.05 (0.97, 1.13)	2.10E-01	6.10E-01	LC/MS Neg			100020536
Decanoylcarnitine (C10)	Lipid	Fatty Acid Metabolism (Acyl Carnitine, Medium Chain)	1.05 (0.97, 1.13)	2.10E-01	6.10E-01	LC/MS Pos Late	HMDB00651	10245190	100001251
Hexadecasphingosine (d16:1)*	Lipid	Sphingosines	0.96 (0.89, 1.03)	2.13E-01	6.12E-01	LC/MS Pos Late			100015723
N-Stearoylserine*	Lipid	Endocannabinoid	0.95 (0.89, 1.03)	2.13E-01	6.12E-01	LC/MS Neg			100019957
Enterolactone sulfate	Xenobiotics	Food Component/Plant	0.95 (0.88, 1.03)	2.13E-01	6.12E-01	LC/MS Neg			100020407
3-Hydroxypyridine sulfate	Xenobiotics	Chemical	1.05 (0.97, 1.13)	2.15E-01	6.12E-01	LC/MS Neg			100006098
SM (d18:1/22:2, d18:2/22:1, d16:1/24:2)*	Lipid	Sphingomyelins	0.95 (0.89, 1.03)	2.15E-01	6.12E-01	LC/MS Pos Late			100006296
Theophylline	Xenobiotics	Xanthine Metabolism	0.95 (0.88, 1.03)	2.15E-01	6.12E-01	LC/MS Neg	HMDB01889	2153	100000437
SM (d18:2/18:1)*	Lipid	Sphingomyelins	0.95 (0.89, 1.03)	2.17E-01	6.13E-01	LC/MS Pos Late			100015788
Sphingosine	Lipid	Sphingosines	0.96 (0.90, 1.03)	2.18E-01	6.13E-01	LC/MS Pos Late	HMDB00252	5353955	297
Betaine	Amino Acid	Glycine, Serine and Threonine Metabolism	0.95 (0.88, 1.03)	2.18E-01	6.13E-01	LC/MS Pos Early	HMDB00043	247	799
Isovalerylglycine	Amino Acid	Leucine, Isoleucine and Valine Metabolism	0.95 (0.89, 1.03)	2.19E-01	6.13E-01	LC/MS Neg	HMDB00678	546304	100001452
Citrate	Energy	TCA Cycle	0.95 (0.89, 1.03)	2.19E-01	6.13E-01	LC/MS Neg	HMDB00094	311	1124
4-Acetamidophenol	Xenobiotics	Drug - Analgesics, Anesthetics	0.96 (0.89, 1.03)	2.19E-01	6.13E-01	LC/MS Pos Early	HMDB01859	1983	1383
γ-Glutamylglutamate	Peptide	Gamma-glutamyl Amino Acid	0.96 (0.89, 1.03)	2.22E-01	6.19E-01	LC/MS Pos Early	HMDB11737	92865	331
Succinoyltaurine	Amino Acid	Methionine, Cysteine, SAM and Taurine Metabolism	0.96 (0.89, 1.03)	2.23E-01	6.19E-01	LC/MS Polar			100021220
Saccharin	Xenobiotics	Food Component/Plant	0.96 (0.89, 1.03)	2.25E-01	6.22E-01	LC/MS Neg	HMDB29723	5143	100000870
DihydroSM (d18:0/20:0, d16:0/22:0)*	Lipid	Dihydrosphingomyelins	1.05 (0.97, 1.13)	2.26E-01	6.22E-01	LC/MS Pos Late			100015786
Linoleoylcholine*	Lipid	Fatty Acid Metabolism (Acyl Choline)	0.96 (0.89, 1.03)	2.27E-01	6.23E-01	LC/MS Pos Late			100015760
Homostachydrine*	Xenobiotics	Food Component/Plant	0.95 (0.88, 1.03)	2.28E-01	6.23E-01	LC/MS Pos Early	HMDB33433	441447	100001550
Glucose	Carbohydrate	Glycolysis, Gluconeogenesis, and Pyruvate Metabolism	0.96 (0.89, 1.03)	2.28E-01	6.23E-01	LC/MS Polar	HMDB00122	5793	572
N-δ-Acetylornithine	Amino Acid	Urea cycle; Arginine and Proline Metabolism	0.95 (0.88, 1.03)	2.30E-01	6.23E-01	LC/MS Polar		9920500	100004523
Pyrraline	Xenobiotics	Food Component/Plant	0.96 (0.89, 1.03)	2.31E-01	6.23E-01	LC/MS Neg	HMDB33143	122228	100001767
Alanine	Amino Acid	Alanine and Aspartate Metabolism	0.96 (0.89, 1.03)	2.33E-01	6.23E-01	LC/MS Pos Early	HMDB00161	5950	811
2-Isopropylmalate	Xenobiotics	Food Component/Plant	1.05 (0.97, 1.12)	2.34E-01	6.23E-01	LC/MS Neg	HMDB00402	77	100000409
Glycosyl ceramide (d18:1/23:1, d17:1/24:1)*	Lipid	Hexosylceramides (HCER)	1.04 (0.97, 1.12)	2.34E-01	6.23E-01	LC/MS Pos Late			100015751
Cortisol	Lipid	Corticosteroids	1.05 (0.97, 1.13)	2.35E-01	6.23E-01	LC/MS Neg	HMDB00063	5754	356
Behenoylcarnitine (C22)*	Lipid	Fatty Acid Metabolism (Acyl Carnitine, Long Chain Saturated)	0.96 (0.89, 1.03)	2.36E-01	6.23E-01	LC/MS Pos Late			100015832
2-Methylserine	Amino Acid	Glycine, Serine and Threonine Metabolism	0.95 (0.88, 1.03)	2.36E-01	6.23E-01	LC/MS Pos Early		94309	100009246
N-Acetyltryptophan	Amino Acid	Tryptophan Metabolism	1.05 (0.97, 1.13)	2.37E-01	6.23E-01	LC/MS Neg	HMDB13713	700653	100001254

Guaiacol sulfate	Xenobiotics	Benzoate Metabolism	1.05 (0.97, 1.13)	2.37E-01	6.23E-01	LC/MS Neg	HMDB60013	22473	100004208
Isovalerate (i5:0)	Amino Acid	Leucine, Isoleucine and Valine Metabolism	0.96 (0.89, 1.03)	2.38E-01	6.23E-01	LC/MS Neg	HMDB00718	10430	100000708
N-Oleoylserine	Lipid	Endocannabinoid	0.96 (0.89, 1.03)	2.38E-01	6.23E-01	LC/MS Neg			100015641
(14 or 15)-Methylpalmitate (a17:0 or i17:0)	Lipid	Fatty Acid, Branched	0.96 (0.89, 1.03)	2.38E-01	6.23E-01	LC/MS Neg			100002945
Hyocholate	Lipid	Secondary Bile Acid Metabolism	0.96 (0.89, 1.03)	2.38E-01	6.23E-01	LC/MS Neg	HMDB00760	92805	100001279
SM (d18:1/17:0, d17:1/18:0, d19:1/16:0)	Lipid	Sphingomyelins	0.96 (0.89, 1.03)	2.40E-01	6.27E-01	LC/MS Pos Late			100008920
Adenosine 5'-diphosphate (ADP)	Nucleotide	Purine Metabolism, Adenine containing	0.96 (0.88, 1.03)	2.45E-01	6.35E-01	LC/MS Neg	HMDB01341	6022	208
N-Acetylcarnosine	Amino Acid	Histidine Metabolism	1.05 (0.97, 1.13)	2.45E-01	6.35E-01	LC/MS Neg	HMDB12881	9903482	100004046
PC (16:0/16:0)	Lipid	Phosphatidylcholine (PC)	0.96 (0.89, 1.03)	2.46E-01	6.35E-01	LC/MS Pos Late	HMDB00564	452110	100000657
LysoPC (20:4n6)*	Lipid	Lysophospholipid	0.96 (0.89, 1.03)	2.46E-01	6.35E-01	LC/MS Pos Late	HMDB10395		100001551
3-Bromo-5-chloro-2,6-dihydroxybenzoic acid*	Xenobiotics	Chemical	0.96 (0.89, 1.03)	2.48E-01	6.36E-01	LC/MS Neg			100021707
5α-Pregnan-3β,20α-diol monosulfate (1)	Lipid	Progestin Steroids	1.05 (0.97, 1.14)	2.49E-01	6.36E-01	LC/MS Neg			100002013
Margaroylcarnitine (C17)*	Lipid	Fatty Acid Metabolism (Acyl Carnitine, Long Chain Saturated)	0.96 (0.89, 1.03)	2.49E-01	6.36E-01	LC/MS Pos Late	HMDB06210		100004054
Dopamine 3-O-sulfate	Amino Acid	Tyrosine Metabolism	0.96 (0.88, 1.03)	2.51E-01	6.36E-01	LC/MS Neg	HMDB06275	122136	100006361
δ-tocopherol	Cofactors and Vitamins	Tocopherol Metabolism	1.04 (0.97, 1.12)	2.51E-01	6.36E-01	LC/MS Pos Late	HMDB02902	92094	100001216
N-Acetyl-2-aminooctanoate*	Lipid	Fatty Acid, Amino	0.95 (0.88, 1.03)	2.51E-01	6.36E-01	LC/MS Neg	HMDB59745	95555	100020204
PC (18:1/22:6)*	Lipid	Phosphatidylcholine (PC)	0.96 (0.89, 1.03)	2.52E-01	6.36E-01	LC/MS Pos Late	HMDB08123		100009130
1-Methylurate	Xenobiotics	Xanthine Metabolism	0.96 (0.89, 1.03)	2.53E-01	6.36E-01	LC/MS Neg	HMDB03099	69726	100001400
2-Hydroxyphytanate*	Lipid	Fatty Acid, Branched	0.96 (0.89, 1.03)	2.55E-01	6.39E-01	LC/MS Neg		189026	100004414
Homocitrulline	Amino Acid	Urea cycle; Arginine and Proline Metabolism	1.05 (0.97, 1.14)	2.55E-01	6.39E-01	LC/MS Polar	HMDB00679	65072	100000963
Genistein sulfate*	Xenobiotics	Food Component/Plant	0.96 (0.89, 1.03)	2.57E-01	6.40E-01	LC/MS Neg			100020207
Octanoylcarnitine (C8)	Lipid	Fatty Acid Metabolism (Acyl Carnitine, Medium Chain)	1.04 (0.97, 1.13)	2.59E-01	6.45E-01	LC/MS Pos Late	HMDB00791	123701	100001247
Glycochenodeoxycholate	Lipid	Primary Bile Acid Metabolism	0.96 (0.89, 1.03)	2.62E-01	6.50E-01	LC/MS Neg	HMDB00637	12544	1628
7-Methylxanthine	Xenobiotics	Xanthine Metabolism	0.96 (0.89, 1.03)	2.63E-01	6.50E-01	LC/MS Pos Early	HMDB01991	68374	100001396
N-Methyltaurine	Amino Acid	Methionine, Cysteine, SAM and Taurine Metabolism	0.96 (0.89, 1.03)	2.64E-01	6.52E-01	LC/MS Polar		7882	100004056
Butyrylglycine	Lipid	Fatty Acid Metabolism (also BCAA Metabolism)	0.96 (0.88, 1.03)	2.66E-01	6.53E-01	LC/MS Neg	HMDB00808	88412	100001151
Indoleacetate	Amino Acid	Tryptophan Metabolism	0.96 (0.89, 1.03)	2.69E-01	6.59E-01	LC/MS Neg	HMDB00197	802	100001034
Retinal	Cofactors and Vitamins	Vitamin A Metabolism	1.04 (0.97, 1.13)	2.72E-01	6.64E-01	LC/MS Pos Late	HMDB01358	638015	100004088
Trans-2-hexenoylglycine	Lipid	Fatty Acid Metabolism (Acyl Glycine)	0.96 (0.89, 1.03)	2.73E-01	6.65E-01	LC/MS Neg			100019884
Glycohyocholate	Lipid	Secondary Bile Acid Metabolism	0.96 (0.89, 1.03)	2.75E-01	6.68E-01	LC/MS Neg			100004083
Imidazole propionate	Amino Acid	Histidine Metabolism	1.04 (0.97, 1.12)	2.76E-01	6.69E-01	LC/MS Pos Early	HMDB02271	70630	100003434

α-CEHC sulfate	Cofactors and Vitamins	Tocopherol Metabolism	1.04 (0.97, 1.12)	2.77E-01	6.69E-01	LC/MS Neg			100005972
5-Hydroxymethyl-2-furoic acid	Amino Acid	Tyrosine Metabolism	1.04 (0.97, 1.12)	2.78E-01	6.69E-01	LC/MS Neg	HMDB02432	80642	100003954
1-Ribosyl-imidazoleacetate*	Amino Acid	Histidine Metabolism	0.96 (0.88, 1.04)	2.80E-01	6.70E-01	LC/MS Pos Early	HMDB02331	5117448	100001668
4-Acetamidobenzoate	Xenobiotics	Chemical	0.96 (0.88, 1.04)	2.81E-01	6.70E-01	LC/MS Neg		19266	100009413
Erythritol	Xenobiotics	Food Component/Plant	0.96 (0.89, 1.03)	2.82E-01	6.70E-01	LC/MS Polar	HMDB02994	222285	100000846
Methyl glucopyranoside (α + β)	Xenobiotics	Food Component/Plant	0.96 (0.88, 1.04)	2.83E-01	6.70E-01	LC/MS Neg			100005864
1,5-Anhydroglucitol (1,5-AG)	Carbohydrate	Glycolysis, Gluconeogenesis, and Pyruvate Metabolism	0.96 (0.89, 1.03)	2.83E-01	6.70E-01	LC/MS Neg	HMDB02712	64960	100000580
Phenol sulfate	Amino Acid	Tyrosine Metabolism	0.96 (0.89, 1.03)	2.84E-01	6.70E-01	LC/MS Neg	HMDB60015	74426	100001510
N-Methylproline	Amino Acid	Urea cycle; Arginine and Proline Metabolism	0.96 (0.89, 1.03)	2.84E-01	6.70E-01	LC/MS Pos Early		557	100001956
Ethylmalonate	Amino Acid	Leucine, Isoleucine and Valine Metabolism	0.96 (0.89, 1.04)	2.84E-01	6.70E-01	LC/MS Polar	HMDB00622	11756	2054
γ-Glutamylthreonine	Peptide	Gamma-glutamyl Amino Acid	0.96 (0.89, 1.04)	2.87E-01	6.75E-01	LC/MS Pos Early	HMDB29159	76078708	100001314
N-Acetyl-1-methylhistidine*	Amino Acid	Histidine Metabolism	1.04 (0.97, 1.13)	2.89E-01	6.77E-01	LC/MS Pos Early		53859791	100004299
3-Carboxy-4-methyl-5-propyl-2-furanpropanoate (CMPF)	Lipid	Fatty Acid, Dicarboxylate	0.96 (0.89, 1.03)	2.90E-01	6.78E-01	LC/MS Neg	HMDB61112	123979	100001178
Pristanate	Lipid	Fatty Acid, Branched	0.96 (0.89, 1.03)	2.91E-01	6.78E-01	LC/MS Neg	HMDB00795	123929	477
Propyl 4-hydroxybenzoate sulfate	Xenobiotics	Benzoate Metabolism	0.96 (0.89, 1.04)	2.93E-01	6.82E-01	LC/MS Neg			100006264
Ornithine	Amino Acid	Urea cycle; Arginine and Proline Metabolism	0.96 (0.89, 1.04)	2.95E-01	6.83E-01	LC/MS Pos Early	HMDB03374	6262	444
PC (14:0/20:4)*	Lipid	Phosphatidylcholine (PC)	0.96 (0.89, 1.04)	2.95E-01	6.83E-01	LC/MS Pos Late	HMDB07883		100009139
3-(3-Hydroxyphenyl)propionate sulfate	Xenobiotics	Benzoate Metabolism	0.96 (0.89, 1.04)	3.02E-01	6.97E-01	LC/MS Neg		187488	100005391
1,2,3-Benzenetriol sulfate (2)	Xenobiotics	Chemical	1.04 (0.97, 1.12)	3.03E-01	6.98E-01	LC/MS Neg			100006374
Stachydrine	Xenobiotics	Food Component/Plant	0.96 (0.89, 1.04)	3.05E-01	6.98E-01	LC/MS Pos Early	HMDB04827	115244	100001296
Ceramide(d17:1/16:0)*	Lipid	Ceramides	0.96 (0.90, 1.03)	3.05E-01	6.98E-01	LC/MS Pos Late			100015731
Cholate	Lipid	Primary Bile Acid Metabolism	0.96 (0.89, 1.04)	3.06E-01	6.98E-01	LC/MS Neg	HMDB00619	221493	136
Biliverdin	Cofactors and Vitamins	Hemoglobin and Porphyrin Metabolism	1.04 (0.97, 1.11)	3.09E-01	7.03E-01	LC/MS Pos Late	HMDB01008	5353439	250
α-Hydroxyisocaproate	Amino Acid	Leucine, Isoleucine and Valine Metabolism	1.04 (0.96, 1.12)	3.11E-01	7.03E-01	LC/MS Neg	HMDB00746	83697	100000706
5α-Pregnan-3β-ol,20-one sulfate	Lipid	Progestin Steroids	1.05 (0.96, 1.14)	3.11E-01	7.03E-01	LC/MS Neg			100002035
Cysteine-glutathione disulfide	Amino Acid	Glutathione Metabolism	0.96 (0.89, 1.04)	3.11E-01	7.03E-01	LC/MS Pos Early	HMDB00656	4247235	100001437
Maltotetraose	Carbohydrate	Glycogen Metabolism	0.96 (0.90, 1.04)	3.12E-01	7.04E-01	LC/MS Neg	HMDB01296	446495	100000275
Iminodiacetate (IDA)	Xenobiotics	Chemical	0.96 (0.89, 1.04)	3.13E-01	7.04E-01	LC/MS Pos Early	HMDB11753	8897	100000900
Sulfate of piperine metabolite C16H19NO3 (2)*	Xenobiotics	Food Component/Plant	1.04 (0.97, 1.12)	3.14E-01	7.05E-01	LC/MS Neg			100020496
Tyramine O-sulfate	Amino Acid	Tyrosine Metabolism	0.96 (0.89, 1.04)	3.16E-01	7.06E-01	LC/MS Neg	HMDB06409	153005	100006092
3-Hydroxyhippurate	Xenobiotics	Benzoate Metabolism	0.96 (0.89, 1.04)	3.17E-01	7.07E-01	LC/MS Neg	HMDB06116	450268	100002122
N-Succinyl-phenylalanine	Amino Acid	Phenylalanine Metabolism	0.96 (0.89, 1.04)	3.18E-01	7.07E-01	LC/MS Neg		3131586	100021472
Caffeic acid sulfate	Xenobiotics	Food Component/Plant	1.04 (0.96, 1.12)	3.21E-01	7.10E-01	LC/MS Polar	HMDB41708		100009337

Proline	Amino Acid	Urea cycle; Arginine and Proline Metabolism	0.96 (0.89, 1.04)	3.21E-01	7.10E-01	LC/MS Pos Early	HMDB00162	145742	480
3-Phenylpropionate (hydrocinnamate)	Xenobiotics	Benzoate Metabolism	0.96 (0.89, 1.04)	3.23E-01	7.13E-01	LC/MS Neg	HMDB00764	107	100000010
5α-Pregnan-3β,20α-diol disulfate	Lipid	Progestin Steroids	1.04 (0.96, 1.14)	3.25E-01	7.15E-01	LC/MS Neg			100001988
Glucuronide of piperine metabolite C17H21NO3 (5)*	Xenobiotics	Food Component/Plant	1.04 (0.96, 1.12)	3.26E-01	7.15E-01	LC/MS Neg			100020493
N-Acetyltaurine	Amino Acid	Methionine, Cysteine, SAM and Taurine Metabolism	0.96 (0.89, 1.04)	3.27E-01	7.15E-01	LC/MS Polar		159864	100005466
Glycosyl-N-palmitoyl-sphingosine (d18:1/16:0)	Lipid	Hexosylceramides (HCER)	0.96 (0.89, 1.04)	3.28E-01	7.15E-01	LC/MS Pos Late			100009272
Diacylglycerol (16:1/18:2 [2], 16:0/18:3 [1])*	Lipid	Diacylglycerol	1.04 (0.96, 1.11)	3.31E-01	7.15E-01	LC/MS Pos Late			100010940
Cholic acid glucuronide	Lipid	Primary Bile Acid Metabolism	0.97 (0.90, 1.03)	3.31E-01	7.15E-01	LC/MS Neg			100020549
Stearate (18:0)	Lipid	Long Chain Saturated Fatty Acid	0.96 (0.90, 1.04)	3.31E-01	7.15E-01	LC/MS Neg	HMDB00827	5281	439
Ascorbic acid 3-sulfate*	Cofactors and Vitamins	Ascorbate and Aldarate Metabolism	0.96 (0.89, 1.04)	3.31E-01	7.15E-01	LC/MS Polar		11425365	100020409
Glutarylcarnitine (C5-DC)	Amino Acid	Lysine Metabolism	1.04 (0.96, 1.12)	3.31E-01	7.15E-01	LC/MS Pos Early	HMDB13130	71464488	100001593
2-O-Methylascorbic acid	Cofactors and Vitamins	Ascorbate and Aldarate Metabolism	1.04 (0.96, 1.13)	3.32E-01	7.15E-01	LC/MS Neg		99779	100003258
Mevalonate	Lipid	Mevalonate Metabolism	1.04 (0.96, 1.12)	3.34E-01	7.15E-01	LC/MS Neg	HMDB00227	439230	30
5-Hydroxyhexanoate	Lipid	Fatty Acid, Monohydroxy	0.96 (0.90, 1.04)	3.35E-01	7.15E-01	LC/MS Neg	HMDB00525	170748	100001148
Theobromine	Xenobiotics	Xanthine Metabolism	0.97 (0.90, 1.04)	3.35E-01	7.15E-01	LC/MS Pos Early	HMDB02825	5429	100000445
Quinolinate	Cofactors and Vitamins	Nicotinate and Nicotinamide Metabolism	1.04 (0.96, 1.12)	3.38E-01	7.21E-01	LC/MS Pos Early	HMDB00232	1066	182
Imidazole lactate	Amino Acid	Histidine Metabolism	1.04 (0.96, 1.13)	3.41E-01	7.23E-01	LC/MS Pos Early	HMDB02320	793	100000263
2,3-Dihydroxy-2-methylbutyrate	Amino Acid	Leucine, Isoleucine and Valine Metabolism	1.04 (0.96, 1.12)	3.42E-01	7.23E-01	LC/MS Neg	HMDB29576	301941	100010869
Phosphate	Energy	Oxidative Phosphorylation	0.96 (0.89, 1.04)	3.44E-01	7.23E-01	LC/MS Pos Early	HMDB01429	1061	461
3-Hydroxysebacate	Lipid	Fatty Acid, Monohydroxy	0.96 (0.90, 1.04)	3.44E-01	7.23E-01	LC/MS Neg	HMDB00350	3017884	100001145
3-Amino-2-piperidone	Amino Acid	Urea cycle; Arginine and Proline Metabolism	0.96 (0.89, 1.04)	3.44E-01	7.23E-01	LC/MS Pos Early	HMDB00323	5200225	100020361
3,5-Dichloro-2,6-dihydroxybenzoic acid	Xenobiotics	Chemical	0.96 (0.89, 1.04)	3.44E-01	7.23E-01	LC/MS Polar			100021324
Phytanate	Xenobiotics	Food Component/Plant	0.97 (0.90, 1.04)	3.46E-01	7.25E-01	LC/MS Neg	HMDB00801	26840	466
Oleoylcholine	Lipid	Fatty Acid Metabolism (Acyl Choline)	0.97 (0.90, 1.04)	3.47E-01	7.25E-01	LC/MS Pos Late			100009331
3-Ethylcatechol sulfate (1)	Xenobiotics	Food Component/Plant	1.04 (0.96, 1.11)	3.48E-01	7.25E-01	LC/MS Neg			100020851
Eugenol sulfate	Xenobiotics	Food Component/Plant	1.03 (0.96, 1.11)	3.48E-01	7.25E-01	LC/MS Neg		180632	100006171
5-Dodecenoylcarnitine (C12:1)	Lipid	Fatty Acid Metabolism (Acyl Carnitine, Monounsaturated)	1.04 (0.96, 1.12)	3.50E-01	7.25E-01	LC/MS Pos Late	HMDB13326		100016069
Adenine	Nucleotide	Purine Metabolism, Adenine containing	0.97 (0.90, 1.04)	3.51E-01	7.25E-01	LC/MS Pos Early	HMDB00034	190	880
Isoursodeoxycholate	Lipid	Secondary Bile Acid Metabolism	0.97 (0.90, 1.04)	3.52E-01	7.25E-01	LC/MS Neg	HMDB00686	127601	100002488
Arabitol/xylitol	Carbohydrate	Pentose Metabolism	0.96 (0.89, 1.04)	3.53E-01	7.25E-01	LC/MS Polar			100006430
PE (18:0/20:4)	Lipid	Phosphatidylethanolamine (PE)	0.97 (0.90, 1.04)	3.54E-01	7.25E-01	LC/MS Pos Late	HMDB09003	5289133	100008977
Quinate	Xenobiotics	Food Component/Plant	1.04 (0.96, 1.12)	3.54E-01	7.25E-01	LC/MS Polar	HMDB03072	6508	100000442

N-Methylpipecolate	Xenobiotics	Bacterial/Fungal	1.04 (0.96, 1.12)	3.55E-01	7.25E-01	LC/MS Pos Early		11286529	100005383
2-Methylcitrate/homocitrate	Energy	TCA Cycle	0.97 (0.90, 1.04)	3.56E-01	7.25E-01	LC/MS Neg			100008929
Tartronate (hydroxymalonate)	Xenobiotics	Food Component/Plant	0.97 (0.90, 1.04)	3.56E-01	7.25E-01	LC/MS Neg	HMDB35227	45	100000840
Dodecadienoate (12:2)*	Lipid	Fatty Acid, Dicarboxylate	0.97 (0.90, 1.04)	3.57E-01	7.25E-01	LC/MS Neg			100020478
S-1-Pyrroline-5-carboxylate	Amino Acid	Glutamate Metabolism	0.97 (0.90, 1.04)	3.57E-01	7.25E-01	LC/MS Pos Early	HMDB01301	1196	35
1-Dihomo-linolenylglycerol (20:3)	Lipid	Monoacylglycerol	0.97 (0.90, 1.04)	3.60E-01	7.28E-01	LC/MS Neg			100006121
4-Acetylphenol sulfate	Xenobiotics	Benzoate Metabolism	1.03 (0.96, 1.11)	3.60E-01	7.28E-01	LC/MS Neg		4684006	100000711
3-Phosphoglycerate	Carbohydrate	Glycolysis, Gluconeogenesis, and Pyruvate Metabolism	0.97 (0.90, 1.04)	3.64E-01	7.32E-01	LC/MS Pos Early	HMDB00807	724	132
N-Acetyl-β-alanine	Nucleotide	Pyrimidine Metabolism, Uracil containing	1.03 (0.96, 1.11)	3.64E-01	7.32E-01	LC/MS Neg		76406	100002102
cis-4-Decenoate (10:1n6)*	Lipid	Medium Chain Fatty Acid	0.97 (0.90, 1.04)	3.67E-01	7.35E-01	LC/MS Neg		5312351	100002796
Thymol sulfate	Xenobiotics	Food Component/Plant	0.97 (0.90, 1.04)	3.68E-01	7.36E-01	LC/MS Neg	HMDB01878		100001757
Tyrosine	Amino Acid	Tyrosine Metabolism	0.97 (0.90, 1.04)	3.71E-01	7.38E-01	LC/MS Pos Early	HMDB00158	6057	815
Palmitoylcarnitine (C16)	Lipid	Fatty Acid Metabolism (Acyl Carnitine, Long Chain Saturated)	0.97 (0.89, 1.04)	3.72E-01	7.38E-01	LC/MS Pos Late	HMDB00222	461	100000776
Taurolithocholate 3-sulfate	Lipid	Secondary Bile Acid Metabolism	0.97 (0.90, 1.04)	3.72E-01	7.38E-01	LC/MS Neg	HMDB02580	440071	100001658
2-Arachidonoylglycerol (20:4)	Lipid	Monoacylglycerol	1.03 (0.96, 1.12)	3.73E-01	7.38E-01	LC/MS Neg	HMDB04666	5282280	100000584
Androsterone glucuronide	Lipid	Androgenic Steroids	1.04 (0.96, 1.12)	3.73E-01	7.38E-01	LC/MS Neg	HMDB02829	114833	100002761
Indolepropionate	Amino Acid	Tryptophan Metabolism	0.97 (0.90, 1.04)	3.76E-01	7.40E-01	LC/MS Neg	HMDB02302	3744	100001083
PC (O-16:0/20:4)*	Lipid	Plasmalogen	0.97 (0.90, 1.04)	3.76E-01	7.40E-01	LC/MS Pos Late	HMDB11220		100009014
N-Stearoyl-sphingadienine (d18:2/18:0)*	Lipid	Ceramides	1.03 (0.96, 1.11)	3.77E-01	7.41E-01	LC/MS Pos Late			100015632
2-Hydroxyoctanoate	Lipid	Fatty Acid, Monohydroxy	0.97 (0.89, 1.04)	3.79E-01	7.41E-01	LC/MS Neg	HMDB02264	94180	100000743
PC (16:0/22:6)	Lipid	Phosphatidylcholine (PC)	0.97 (0.90, 1.04)	3.80E-01	7.41E-01	LC/MS Pos Late	HMDB07991	6441886	100008915
Sulfate of piperine metabolite C16H19NO3 (3)*	Xenobiotics	Food Component/Plant	1.03 (0.96, 1.11)	3.81E-01	7.41E-01	LC/MS Neg			100020497
Phenylalanine	Amino Acid	Phenylalanine Metabolism	0.97 (0.90, 1.04)	3.81E-01	7.41E-01	LC/MS Pos Early	HMDB00159	6140	460
fructosyllysine	Amino Acid	Lysine Metabolism	0.97 (0.90, 1.04)	3.82E-01	7.41E-01	LC/MS Pos Early		123708	100005373
Dodecanedioate (C12-DC)	Lipid	Fatty Acid, Dicarboxylate	0.97 (0.90, 1.04)	3.82E-01	7.41E-01	LC/MS Neg	HMDB00623	12736	100001102
N,N,N-Trimethyl-5-aminovalerate	Amino Acid	Lysine Metabolism	0.97 (0.89, 1.05)	3.84E-01	7.41E-01	LC/MS Pos Early		14274897	100015962
DihydroSM (d18:0/14:0)*	Lipid	Dihydrosphingomyelins	0.97 (0.90, 1.04)	3.85E-01	7.41E-01	LC/MS Pos Late	HMDB12085		100009038
S-Allylcysteine	Xenobiotics	Food Component/Plant	0.97 (0.90, 1.04)	3.86E-01	7.41E-01	LC/MS Pos Early	HMDB34323	98280	100004509
3-Decenoylcarnitine	Lipid	Fatty Acid Metabolism (Acyl Carnitine, Monounsaturated)	0.97 (0.90, 1.04)	3.86E-01	7.41E-01	LC/MS Pos Late			100021136
C-Glycosyltryptophan	Amino Acid	Tryptophan Metabolism	1.04 (0.96, 1.12)	3.87E-01	7.41E-01	LC/MS Neg		10981970	100006379
5-Hydroxylysine	Amino Acid	Lysine Metabolism	1.03 (0.96, 1.12)	3.89E-01	7.44E-01	LC/MS Pos Early	HMDB00450	1029	100000054
N-Stearoyl-sphingosine (d18:1/18:0)*	Lipid	Ceramides	1.03 (0.96, 1.12)	3.92E-01	7.47E-01	LC/MS Pos Late	HMDB04950	5283565	1547
Oleate/vaccenate (18:1)	Lipid	Long Chain Monounsaturated Fatty Acid	0.97 (0.90, 1.04)	3.93E-01	7.48E-01	LC/MS Neg			100008930

Glucuronide of piperine metabolite C17H21NO3 (3)*	Xenobiotics	Food Component/Plant	1.03 (0.96, 1.11)	3.96E-01	7.50E-01	LC/MS Neg			100020491
1-Carboxyethylleucine	Amino Acid	Leucine, Isoleucine and Valine Metabolism	1.03 (0.96, 1.12)	3.97E-01	7.50E-01	LC/MS Neg			100020425
Caprylate (8:0)	Lipid	Medium Chain Fatty Acid	1.03 (0.96, 1.11)	3.97E-01	7.50E-01	LC/MS Neg	HMDB00482	379	932
Pregnanediol-3-glucuronide	Lipid	Progestin Steroids	1.04 (0.95, 1.13)	3.98E-01	7.50E-01	LC/MS Neg	HMDB10318	123796	100003470
Acetylcarnitine (C2)	Lipid	Fatty Acid Metabolism (Acyl Carnitine, Short Chain)	0.97 (0.90, 1.04)	4.02E-01	7.56E-01	LC/MS Pos Early	HMDB00201	1	100000802
5α-Pregnan-3β,20α-diol monosulfate (2)	Lipid	Progestin Steroids	1.04 (0.95, 1.13)	4.02E-01	7.56E-01	LC/MS Neg			100002014
4-Methyl-2-oxopentanoate	Amino Acid	Leucine, Isoleucine and Valine Metabolism	0.97 (0.90, 1.04)	4.05E-01	7.59E-01	LC/MS Neg	HMDB00695	70	100000551
LysoPI (20:4)*	Lipid	Lysophospholipid	0.97 (0.90, 1.04)	4.08E-01	7.61E-01	LC/MS Neg	HMDB61690		100001654
3-Methylxanthine	Xenobiotics	Xanthine Metabolism	0.97 (0.90, 1.04)	4.08E-01	7.61E-01	LC/MS Neg	HMDB01886	70639	100001108
N2-Acetyl,N6,N6-dimethyllysine	Amino Acid	Lysine Metabolism	1.03 (0.96, 1.11)	4.10E-01	7.61E-01	LC/MS Pos Early			100020545
α-Hydroxyisovalerate	Amino Acid	Leucine, Isoleucine and Valine Metabolism	0.97 (0.89, 1.05)	4.10E-01	7.61E-01	LC/MS Neg	HMDB00407	99823	100001300
Glycodeoxycholate glucuronide (1)	Lipid	Secondary Bile Acid Metabolism	0.97 (0.90, 1.05)	4.10E-01	7.61E-01	LC/MS Polar			100009266
Palmitoylcholine	Lipid	Fatty Acid Metabolism (Acyl Choline)	0.97 (0.90, 1.04)	4.12E-01	7.63E-01	LC/MS Pos Late		151731	100009233
Aspartate	Amino Acid	Alanine and Aspartate Metabolism	0.97 (0.91, 1.04)	4.14E-01	7.64E-01	LC/MS Pos Early	HMDB00191	5960	234
Inosine	Nucleotide	Purine Metabolism, (Hypo)Xanthine/Inosine containing	1.03 (0.96, 1.11)	4.17E-01	7.64E-01	LC/MS Neg	HMDB00195	6021	361
Palmitoyl-linoleoyl-glycerol (16:0/18:2) [2]*	Lipid	Diacylglycerol	1.03 (0.96, 1.11)	4.18E-01	7.64E-01	LC/MS Pos Late			100009055
Adrenate (22:4n6)	Lipid	Long Chain Polyunsaturated Fatty Acid (n3 and n6)	1.03 (0.96, 1.11)	4.18E-01	7.64E-01	LC/MS Neg	HMDB02226	5497181	100001193
Ceramide (d18:1/17:0, d17:1/18:0)*	Lipid	Ceramides	1.03 (0.96, 1.11)	4.18E-01	7.64E-01	LC/MS Pos Late			100015737
1-Linoleoylglycerol (18:2)	Lipid	Monoacylglycerol	0.97 (0.90, 1.04)	4.19E-01	7.64E-01	LC/MS Neg		5283469	100001040
tetradecadienoate (14:2)*	Lipid	Long Chain Polyunsaturated Fatty Acid (n3 and n6)	0.97 (0.90, 1.04)	4.19E-01	7.64E-01	LC/MS Neg	HMDB00560	5312409	100020343
N-Acetyl-3-methylhistidine*	Amino Acid	Histidine Metabolism	0.97 (0.90, 1.04)	4.21E-01	7.65E-01	LC/MS Neg		193270	100002204
N,N,N-Trimethyl-alanylproline betaine (TMAP)	Amino Acid	Urea cycle; Arginine and Proline Metabolism	0.97 (0.90, 1.05)	4.22E-01	7.65E-01	LC/MS Pos Early			100020217
Dihydroxyacetone phosphate (DHAP)	Carbohydrate	Glycolysis, Gluconeogenesis, and Pyruvate Metabolism	0.97 (0.90, 1.05)	4.22E-01	7.65E-01	LC/MS Pos Early	HMDB01473	668	309
PC (16:0/20:4n6)	Lipid	Phosphatidylcholine (PC)	0.97 (0.90, 1.05)	4.24E-01	7.66E-01	LC/MS Pos Late	HMDB07982	10747814	100008914
Retinol (Vitamin A)	Cofactors and Vitamins	Vitamin A Metabolism	0.97 (0.89, 1.05)	4.27E-01	7.71E-01	LC/MS Pos Late	HMDB00305	445354	498
Chiro-inositol	Lipid	Inositol Metabolism	0.97 (0.90, 1.05)	4.28E-01	7.71E-01	LC/MS Polar	HMDB34220		100001859
Serotonin	Amino Acid	Tryptophan Metabolism	0.97 (0.90, 1.05)	4.31E-01	7.74E-01	LC/MS Pos Early	HMDB00259	5202	504
Xanthurenate	Amino Acid	Tryptophan Metabolism	1.03 (0.96, 1.11)	4.33E-01	7.76E-01	LC/MS Pos Early	HMDB00881	5699	100000015
Deoxycholic acid glucuronide	Lipid	Secondary Bile Acid Metabolism	0.97 (0.90, 1.04)	4.34E-01	7.76E-01	LC/MS Neg			100020550
Bilirubin (E,Z or Z,E)*	Cofactors and Vitamins	Hemoglobin and Porphyrin Metabolism	0.97 (0.90, 1.05)	4.36E-01	7.76E-01	LC/MS Pos Late	HMDB00488	5799469	100001951
2-Acetamidophenol sulfate	Xenobiotics	Food Component/Plant	0.97 (0.90, 1.05)	4.36E-01	7.76E-01	LC/MS Neg		181671	100006190

N-Acetyl-isoputreanine	Amino Acid	Polyamine Metabolism	0.97 (0.90, 1.05)	4.37E-01	7.76E-01	LC/MS Pos Early		131802901	100020487
3-(4-Hydroxyphenyl)lactate	Amino Acid	Tyrosine Metabolism	1.03 (0.95, 1.11)	4.39E-01	7.79E-01	LC/MS Neg	HMDB00755	9378	240
Perfluorooctanesulfonate (PFOS)	Xenobiotics	Chemical	0.97 (0.90, 1.05)	4.40E-01	7.80E-01	LC/MS Polar	HMDB59586	74483	100010955
β-Alanine	Nucleotide	Pyrimidine Metabolism, Uracil containing	1.03 (0.96, 1.11)	4.42E-01	7.82E-01	LC/MS Pos Early	HMDB00056	239	244
Picolinoylglycine	Lipid	Fatty Acid Metabolism (Acyl Glycine)	1.03 (0.95, 1.11)	4.45E-01	7.83E-01	LC/MS Pos Early	HMDB59766	11788622	100021198
2-Hydroxyglutarate	Lipid	Fatty Acid, Dicarboxylate	0.97 (0.90, 1.05)	4.46E-01	7.83E-01	LC/MS Pos Early	HMDB0059655	43	100002070
Carnitine	Lipid	Carnitine Metabolism	0.97 (0.90, 1.05)	4.46E-01	7.83E-01	LC/MS Pos Early	HMDB00062	10917	100000007
5-Acetylamino-6-amino-3-methyluracil	Xenobiotics	Xanthine Metabolism	0.97 (0.90, 1.05)	4.47E-01	7.83E-01	LC/MS Polar	HMDB04400	88299	100001403
Catechol sulfate	Xenobiotics	Benzoate Metabolism	1.03 (0.95, 1.11)	4.47E-01	7.83E-01	LC/MS Neg	HMDB59724	3083879	100001605
Glycodeoxycholate 3-sulfate	Lipid	Secondary Bile Acid Metabolism	0.97 (0.90, 1.05)	4.50E-01	7.87E-01	LC/MS Neg			100006642
Isobutyrylglycine	Amino Acid	Leucine, Isoleucine and Valine Metabolism	0.97 (0.90, 1.05)	4.55E-01	7.89E-01	LC/MS Neg	HMDB00730	10855600	100001590
Phenyllactate (PLA)	Amino Acid	Phenylalanine Metabolism	0.97 (0.90, 1.05)	4.57E-01	7.89E-01	LC/MS Neg	HMDB00779	3848	100000774
Choline	Lipid	Phospholipid Metabolism	0.97 (0.90, 1.05)	4.57E-01	7.89E-01	LC/MS Pos Early	HMDB00097	305	1256
LysoPI (18:0)	Lipid	Lysophospholipid	0.97 (0.90, 1.05)	4.57E-01	7.89E-01	LC/MS Neg	HMDB61696		100000656
Ximenoylcarnitine (C26:1)*	Lipid	Fatty Acid Metabolism (Acyl Carnitine, Monounsaturated)	0.97 (0.90, 1.05)	4.57E-01	7.89E-01	LC/MS Pos Late			100015836
Fructose	Carbohydrate	Fructose, Mannose and Galactose Metabolism	0.97 (0.90, 1.05)	4.57E-01	7.89E-01	LC/MS Polar	HMDB00660	5984	878
Dopamine 4-sulfate	Amino Acid	Tyrosine Metabolism	0.97 (0.90, 1.05)	4.61E-01	7.92E-01	LC/MS Neg	HMDB04148	123932	100006360
Xanthine	Nucleotide	Purine Metabolism, (Hypo)Xanthine/Inosine containing	1.03 (0.95, 1.11)	4.61E-01	7.92E-01	LC/MS Polar	HMDB00292	1188	1004
4-Allylcatechol sulfate	Xenobiotics	Benzoate Metabolism	1.03 (0.96, 1.11)	4.63E-01	7.92E-01	LC/MS Neg			100020578
Isobutyrylcarnitine (C4)	Amino Acid	Leucine, Isoleucine and Valine Metabolism	0.97 (0.90, 1.05)	4.64E-01	7.92E-01	LC/MS Pos Early	HMDB00736	168379	100001055
2-Aminobutyrate	Amino Acid	Glutathione Metabolism	0.97 (0.90, 1.05)	4.64E-01	7.92E-01	LC/MS Pos Early	HMDB00650	439691	1128
3-(4-Hydroxyphenyl)propionate	Xenobiotics	Benzoate Metabolism	0.97 (0.91, 1.05)	4.66E-01	7.92E-01	LC/MS Neg	HMDB02199	10394	100002914
Isoleucylglycine	Peptide	Dipeptide	0.97 (0.90, 1.05)	4.66E-01	7.92E-01	LC/MS Neg	HMDB28907	342532	100003169
Taurochenodeoxycholic acid 3-sulfate	Lipid	Secondary Bile Acid Metabolism	1.03 (0.95, 1.12)	4.66E-01	7.92E-01	LC/MS Neg	HMDB02486		100021093
Cysteinylglycine	Amino Acid	Glutathione Metabolism	1.03 (0.95, 1.11)	4.67E-01	7.92E-01	LC/MS Pos Early	HMDB00078	439498	278
(16 or 17)-Methylstearate (a19:0 or i19:0)	Lipid	Fatty Acid, Branched	0.97 (0.91, 1.05)	4.69E-01	7.93E-01	LC/MS Neg	HMDB37397	3083779	100002356
4-Acetamidobutanoate	Amino Acid	Polyamine Metabolism	0.97 (0.89, 1.05)	4.70E-01	7.93E-01	LC/MS Pos Early	HMDB03681	18189	1113
Mannitol/sorbitol	Carbohydrate	Fructose, Mannose and Galactose Metabolism	0.97 (0.90, 1.05)	4.71E-01	7.95E-01	LC/MS Polar	HMDB00247	5780	100001740
Hexanoylcarnitine (C6)	Lipid	Fatty Acid Metabolism (Acyl Carnitine, Medium Chain)	1.03 (0.95, 1.11)	4.79E-01	8.05E-01	LC/MS Pos Early	HMDB00705	6426853	100000781
2,3-Dihydroxy-5-methylthio-4-pentenoate (DMTPA)*	Amino Acid	Methionine, Cysteine, SAM and Taurine Metabolism	1.03 (0.95, 1.12)	4.79E-01	8.05E-01	LC/MS Neg			100020893
3-Methylglutaconate	Amino Acid	Leucine, Isoleucine and Valine Metabolism	0.97 (0.90, 1.05)	4.82E-01	8.06E-01	LC/MS Polar	HMDB00522	1551553	100002458

Uracil	Nucleotide	Pyrimidine Metabolism, Uracil containing	0.97 (0.91, 1.05)	4.82E-01	8.06E-01	LC/MS Polar	HMDB00300	1174	825
Ursodeoxycholate	Lipid	Secondary Bile Acid Metabolism	0.97 (0.91, 1.05)	4.83E-01	8.06E-01	LC/MS Neg	HMDB00946	31401	1135
Estrone 3-sulfate	Lipid	Estrogenic Steroids	1.03 (0.95, 1.12)	4.85E-01	8.07E-01	LC/MS Neg	HMDB01425	3001028	1230
N-Palmitoylglycine	Lipid	Fatty Acid Metabolism (Acyl Glycine)	0.97 (0.90, 1.05)	4.87E-01	8.07E-01	LC/MS Neg	HMDB13034	151008	100003686
Cystathionine	Amino Acid	Methionine, Cysteine, SAM and Taurine Metabolism	1.03 (0.95, 1.11)	4.87E-01	8.07E-01	LC/MS Pos Early	HMDB00099	439258	310
3-Methyl-2-oxobutyrate	Amino Acid	Leucine, Isoleucine and Valine Metabolism	0.97 (0.90, 1.05)	4.88E-01	8.07E-01	LC/MS Neg	HMDB00019	49	100000936
2-Hydroxyphenylacetate	Amino Acid	Phenylalanine Metabolism	1.03 (0.95, 1.11)	4.88E-01	8.07E-01	LC/MS Polar	HMDB00669	11970	235
LysoPI (16:0)	Lipid	Lysophospholipid	0.97 (0.90, 1.05)	4.91E-01	8.11E-01	LC/MS Neg	HMDB61695		100001655
PE (16:0/18:1)	Lipid	Phosphatidylethanolamine (PE)	0.97 (0.90, 1.05)	4.94E-01	8.13E-01	LC/MS Pos Late	HMDB05320	5283496	1526
Pyridoxate	Cofactors and Vitamins	Vitamin B6 Metabolism	0.97 (0.90, 1.05)	4.97E-01	8.13E-01	LC/MS Neg	HMDB00017	6723	100001121
SM (d18:1/20:2, d18:2/20:1, d16:1/22:2)*	Lipid	Sphingomyelins	0.97 (0.90, 1.05)	4.97E-01	8.13E-01	LC/MS Pos Late			100006293
3-Hydroxyoctanoate	Lipid	Fatty Acid, Monohydroxy	0.97 (0.91, 1.05)	5.01E-01	8.13E-01	LC/MS Neg	HMDB01954	26613	100000773
10-Nonadecenoate (19:1n9)	Lipid	Long Chain Monounsaturated Fatty Acid	0.98 (0.91, 1.05)	5.01E-01	8.13E-01	LC/MS Neg	HMDB13622	5312513	100001277
Ceramide (d18:1/14:0, d16:1/16:0)*	Lipid	Ceramides	1.02 (0.95, 1.10)	5.01E-01	8.13E-01	LC/MS Pos Late			100015735
Pantoate	Cofactors and Vitamins	Pantothenate and CoA Metabolism	0.97 (0.90, 1.05)	5.02E-01	8.13E-01	LC/MS Neg		439251	100002613
Hexanoylglutamine	Lipid	Fatty Acid Metabolism (Acyl Glutamine)	0.97 (0.90, 1.05)	5.03E-01	8.13E-01	LC/MS Neg			100001733
PE (16:0/20:4)*	Lipid	Phosphatidylethanolamine (PE)	0.97 (0.90, 1.05)	5.03E-01	8.13E-01	LC/MS Pos Late	HMDB05323	9546800	100008990
Carboxyibuprofen	Xenobiotics	Drug - Analgesics, Anesthetics	0.97 (0.90, 1.05)	5.04E-01	8.13E-01	LC/MS Neg	HMDB60564	10444113	100004171
Eicosenoate (20:1)	Lipid	Long Chain Monounsaturated Fatty Acid	0.98 (0.91, 1.05)	5.05E-01	8.13E-01	LC/MS Neg	HMDB02231	5282768	100001335
2R,3R-Dihydroxybutyrate	Lipid	Fatty Acid, Dihydroxy	1.03 (0.95, 1.11)	5.06E-01	8.13E-01	LC/MS Polar	HMDB00498	13120901	100002367
Mannose	Carbohydrate	Fructose, Mannose and Galactose Metabolism	1.03 (0.95, 1.11)	5.06E-01	8.13E-01	LC/MS Polar	HMDB00169	18950	803
LysoPC (16:1)*	Lipid	Lysophospholipid	0.97 (0.90, 1.05)	5.06E-01	8.13E-01	LC/MS Pos Late	HMDB10383	24779461	100001511
Arachidonoylcholine	Lipid	Fatty Acid Metabolism (Acyl Choline)	0.98 (0.91, 1.05)	5.07E-01	8.13E-01	LC/MS Pos Late			100009332
Tetradecanedioate (C14-DC)	Lipid	Fatty Acid, Dicarboxylate	0.98 (0.91, 1.05)	5.09E-01	8.13E-01	LC/MS Neg	HMDB00872	13185	100001613
Ethyl maltol sulfate	Xenobiotics	Food Component/Plant	1.03 (0.95, 1.10)	5.10E-01	8.13E-01	LC/MS Neg			100006255
9-Hydroxystearate	Lipid	Fatty Acid, Monohydroxy	0.98 (0.91, 1.05)	5.10E-01	8.13E-01	LC/MS Neg	HMDB61661	9570127	100005834
Trimethylamine N-oxide	Lipid	Phospholipid Metabolism	0.97 (0.90, 1.05)	5.10E-01	8.13E-01	LC/MS Pos Early	HMDB00925	1145	100003397
Homoarginine	Amino Acid	Urea cycle; Arginine and Proline Metabolism	1.03 (0.95, 1.11)	5.12E-01	8.14E-01	LC/MS Pos Early	HMDB00670	9085	100000961
PE (16:0/22:6)*	Lipid	Phosphatidylethanolamine (PE)	0.97 (0.90, 1.05)	5.14E-01	8.15E-01	LC/MS Pos Late	HMDB05324	9546799	100008991
Pyroglutamine*	Amino Acid	Glutamate Metabolism	0.98 (0.90, 1.05)	5.17E-01	8.17E-01	LC/MS Pos Early			100001540
(2 or 3)-Decenoate (10:1n7 or n8)	Lipid	Medium Chain Fatty Acid	0.98 (0.91, 1.05)	5.17E-01	8.17E-01	LC/MS Neg			100021502
Succinylcarnitine (C4-DC)	Energy	TCA Cycle	1.03 (0.95, 1.11)	5.18E-01	8.17E-01	LC/MS Pos Early	HMDB61717	71464481	100001948

Glucuronide of piperine metabolite C17H21NO3 (4)*	Xenobiotics	Food Component/Plant	1.02 (0.95, 1.10)	5.21E-01	8.20E-01	LC/MS Neg			100020492
Glucuronate	Carbohydrate	Aminosugar Metabolism	0.98 (0.91, 1.05)	5.22E-01	8.20E-01	LC/MS Polar	HMDB00127	444791	100000257
2-Aminophenol sulfate	Xenobiotics	Food Component/Plant	0.98 (0.91, 1.05)	5.22E-01	8.20E-01	LC/MS Neg	HMDB61116	181670	100004322
Stearidonate (18:4n3)	Lipid	Long Chain Polyunsaturated Fatty Acid (n3 and n6)	0.98 (0.91, 1.05)	5.23E-01	8.20E-01	LC/MS Neg	HMDB06547	5312508	100001229
Glycochenodeoxycholate glucuronide (1)	Lipid	Primary Bile Acid Metabolism	0.98 (0.90, 1.05)	5.26E-01	8.21E-01	LC/MS Neg			100009264
Trans-4-hydroxyproline	Amino Acid	Urea cycle; Arginine and Proline Metabolism	0.98 (0.90, 1.05)	5.27E-01	8.21E-01	LC/MS Pos Early	HMDB00725	5810	1001
Taurodeoxycholic acid 3-sulfate	Lipid	Secondary Bile Acid Metabolism	0.98 (0.90, 1.05)	5.27E-01	8.21E-01	LC/MS Neg			100006644
Tartarate	Xenobiotics	Food Component/Plant	0.98 (0.91, 1.05)	5.31E-01	8.25E-01	LC/MS Neg	HMDB00956	444305	100000295
Dimethylglycine	Amino Acid	Glycine, Serine and Threonine Metabolism	1.02 (0.95, 1.10)	5.31E-01	8.25E-01	LC/MS Pos Early	HMDB00092	673	806
Dihydroferulate	Xenobiotics	Food Component/Plant	0.98 (0.90, 1.05)	5.32E-01	8.25E-01	LC/MS Neg		14340	100003432
2-Linoleoylglycerol (18:2)	Lipid	Monoacylglycerol	0.98 (0.91, 1.05)	5.33E-01	8.25E-01	LC/MS Neg	HMDB11538	5365676	100000987
Pantothenate	Cofactors and Vitamins	Pantothenate and CoA Metabolism	0.98 (0.90, 1.06)	5.35E-01	8.25E-01	LC/MS Neg	HMDB00210	6613	1024
Margarate (17:0)	Lipid	Long Chain Saturated Fatty Acid	0.98 (0.91, 1.05)	5.36E-01	8.25E-01	LC/MS Neg	HMDB02259	10465	891
Carotene diol (3)	Cofactors and Vitamins	Vitamin A Metabolism	0.98 (0.91, 1.05)	5.36E-01	8.25E-01	LC/MS Pos Late			100015968
Dihydrocaffeate sulfate (2)	Xenobiotics	Food Component/Plant	1.02 (0.95, 1.10)	5.37E-01	8.25E-01	LC/MS Neg			100020822
Etiocholanolone glucuronide	Lipid	Androgenic Steroids	1.02 (0.95, 1.10)	5.39E-01	8.27E-01	LC/MS Neg	HMDB04484	270605	100005403
Docosapentaenoate (n3 DPA; 22:5n3)	Lipid	Long Chain Polyunsaturated Fatty Acid (n3 and n6)	0.98 (0.91, 1.05)	5.40E-01	8.27E-01	LC/MS Neg	HMDB06528	6441454	100001181
Creatine	Amino Acid	Creatine Metabolism	0.98 (0.91, 1.05)	5.42E-01	8.27E-01	LC/MS Neg	HMDB00064	586	1221
α-CEHC	Cofactors and Vitamins	Tocopherol Metabolism	1.02 (0.95, 1.10)	5.43E-01	8.27E-01	LC/MS Neg	HMDB01518	9943542	100002206
Docosahexaenoylcholine	Lipid	Fatty Acid Metabolism (Acyl Choline)	0.98 (0.91, 1.05)	5.43E-01	8.27E-01	LC/MS Pos Late			100009333
N-Acetylproline	Amino Acid	Urea cycle; Arginine and Proline Metabolism	0.98 (0.91, 1.05)	5.46E-01	8.30E-01	LC/MS Pos Early		322640	100001334
N-Formylmethionine	Amino Acid	Methionine, Cysteine, SAM and Taurine Metabolism	0.98 (0.90, 1.06)	5.47E-01	8.30E-01	LC/MS Neg	HMDB01015	439750	194
Lactosyl-N-palmitoyl-sphingosine (d18:1/16:0)	Lipid	Lactosylceramides (LCER)	0.98 (0.91, 1.05)	5.48E-01	8.31E-01	LC/MS Pos Late			100009030
Methylsuccinoylcarnitine	Amino Acid	Leucine, Isoleucine and Valine Metabolism	0.98 (0.90, 1.06)	5.50E-01	8.32E-01	LC/MS Pos Early			100009275
PS (18:0/18:1)	Lipid	Phosphatidylserine (PS)	0.98 (0.91, 1.05)	5.53E-01	8.33E-01	LC/MS Pos Late	HMDB10163	9547087	100000639
δ-CEHC	Cofactors and Vitamins	Tocopherol Metabolism	0.98 (0.91, 1.05)	5.53E-01	8.33E-01	LC/MS Neg		11536125	100020416
Triclosan sulfate	Xenobiotics	Drug - Antibiotic	0.98 (0.91, 1.05)	5.55E-01	8.34E-01	LC/MS Neg	HMDB61387		100020610
PE (18:0/22:6)*	Lipid	Phosphatidylethanolamine (PE)	1.02 (0.95, 1.10)	5.57E-01	8.37E-01	LC/MS Pos Late	HMDB05334	9546798	100008992
2-Methoxyhydroquinone sulfate (1)	Xenobiotics	Benzoate Metabolism	0.98 (0.91, 1.05)	5.62E-01	8.42E-01	LC/MS Neg			100021839
Pimeloylcarnitine/3-methyladipoylcarnitine (C7-DC)	Lipid	Fatty Acid Metabolism (Acyl Carnitine, Dicarboxylate)	0.98 (0.91, 1.05)	5.62E-01	8.42E-01	LC/MS Pos Early			100009407
Stearoylcholine*	Lipid	Fatty Acid Metabolism (Acyl Choline)	0.98 (0.91, 1.05)	5.65E-01	8.44E-01	LC/MS Pos Late			100015759

O-Sulfo-L-tyrosine	Xenobiotics	Chemical	0.98 (0.90, 1.06)	5.67E-01	8.44E-01	LC/MS Neg		514186	100005384
4-Vinylguaiacol glucuronide	Xenobiotics	Food Component/Plant	1.02 (0.95, 1.10)	5.67E-01	8.44E-01	LC/MS Neg			100021209
6-Oxopiperidine-2-carboxylate	Amino Acid	Lysine Metabolism	1.02 (0.95, 1.10)	5.68E-01	8.44E-01	LC/MS Neg	HMDB61705	3014237	100004499
1-Methyl-4-imidazoleacetate	Amino Acid	Histidine Metabolism	0.98 (0.91, 1.06)	5.69E-01	8.44E-01	LC/MS Pos Early	HMDB02820	75810	100001208
3-Methyl-2-oxovalerate	Amino Acid	Leucine, Isoleucine and Valine Metabolism	0.98 (0.91, 1.06)	5.70E-01	8.44E-01	LC/MS Neg	HMDB03736	47	100000036
1,3-Dimethylurate	Xenobiotics	Xanthine Metabolism	0.98 (0.91, 1.06)	5.71E-01	8.44E-01	LC/MS Neg	HMDB01857	70346	100001106
3-Hydroxy-2-ethylpropionate	Amino Acid	Leucine, Isoleucine and Valine Metabolism	0.98 (0.91, 1.05)	5.73E-01	8.45E-01	LC/MS Neg	HMDB00396	188979	100001170
Trans-3,4-methyleneheptanoate	Xenobiotics	Food Component/Plant	0.98 (0.91, 1.05)	5.74E-01	8.45E-01	LC/MS Neg			100006651
Myo-inositol	Lipid	Inositol Metabolism	0.98 (0.91, 1.06)	5.74E-01	8.45E-01	LC/MS Polar	HMDB00211	892	363
α-CEHC glucuronide*	Cofactors and Vitamins	Tocopherol Metabolism	1.02 (0.94, 1.11)	5.78E-01	8.47E-01	LC/MS Neg		134819285	100003101
SM (d18:1/18:0)	Lipid	Sphingomyelins	1.02 (0.95, 1.10)	5.80E-01	8.47E-01	LC/MS Pos Late	HMDB01348	6453725	1538
Octadecanedioate (C18-DC)	Lipid	Fatty Acid, Dicarboxylate	0.98 (0.91, 1.06)	5.81E-01	8.47E-01	LC/MS Neg	HMDB00782	70095	100001615
Xylose	Carbohydrate	Pentose Metabolism	0.98 (0.91, 1.06)	5.81E-01	8.47E-01	LC/MS Polar	HMDB00098	135191	826
PC (18:0/20:4)	Lipid	Phosphatidylcholine (PC)	0.98 (0.91, 1.06)	5.82E-01	8.47E-01	LC/MS Pos Late	HMDB08048	16219824	100001869
1-Linolenoylglycerol (18:3)	Lipid	Monoacylglycerol	0.98 (0.91, 1.05)	5.82E-01	8.47E-01	LC/MS Neg	HMDB11569	53480978	100001435
Sphingadienine	Lipid	Sphingolipid Synthesis	0.98 (0.92, 1.05)	5.83E-01	8.47E-01	LC/MS Pos Late		6449795	100015643
Lactose	Carbohydrate	Disaccharides and Oligosaccharides	0.98 (0.91, 1.05)	5.83E-01	8.47E-01	LC/MS Polar	HMDB00186	84571	393
Hexadecadienoate (16:2n6)	Lipid	Long Chain Polyunsaturated Fatty Acid (n3 and n6)	0.98 (0.91, 1.05)	5.86E-01	8.49E-01	LC/MS Neg	HMDB00477	13932172	100009394
Benzoate	Xenobiotics	Benzoate Metabolism	0.98 (0.91, 1.05)	5.87E-01	8.49E-01	LC/MS Neg	HMDB01870	243	100000008
N-Acetylphenylalanine	Amino Acid	Phenylalanine Metabolism	1.02 (0.95, 1.10)	5.92E-01	8.54E-01	LC/MS Neg	HMDB00512	74839	100001256
N-Carbamoylalanine	Amino Acid	Alanine and Aspartate Metabolism	0.98 (0.90, 1.06)	5.92E-01	8.54E-01	LC/MS Pos Early		426409	100006369
Vanillic acid glycine	Xenobiotics	Food Component/Plant	0.98 (0.91, 1.06)	5.94E-01	8.55E-01	LC/MS Neg	HMDB60026	3083688	100021363
3-Hydroxydecanoylcarnitine	Lipid	Fatty Acid Metabolism (Acyl Carnitine, Hydroxy)	0.98 (0.91, 1.06)	5.95E-01	8.55E-01	LC/MS Pos Late	HMDB61636		100021141
Indolepropionylglycine	Amino Acid	Tryptophan Metabolism	0.98 (0.91, 1.06)	5.98E-01	8.55E-01	LC/MS Neg		7677842	100010863
Methylsuccinate	Amino Acid	Leucine, Isoleucine and Valine Metabolism	0.98 (0.91, 1.06)	5.98E-01	8.55E-01	LC/MS Polar	HMDB01844	10349	2051
5-Hydroxy-2-methylpyridine sulfate	Xenobiotics	Chemical	0.98 (0.91, 1.06)	5.99E-01	8.55E-01	LC/MS Neg			100020973
Undecenoylcarnitine (C11:1)	Lipid	Fatty Acid Metabolism (Acyl Carnitine, Monounsaturated)	1.02 (0.95, 1.10)	6.00E-01	8.55E-01	LC/MS Pos Late			100021131
Dibutyl sulfosuccinate	Xenobiotics	Chemical	1.02 (0.95, 1.09)	6.00E-01	8.55E-01	LC/MS Neg		423891	100021538
Furaneol sulfate	Xenobiotics	Food Component/Plant	1.02 (0.95, 1.10)	6.02E-01	8.56E-01	LC/MS Neg			100009328
Maltose	Carbohydrate	Glycogen Metabolism	0.98 (0.91, 1.05)	6.03E-01	8.56E-01	LC/MS Polar	HMDB00163	10991489	913
2-Hydroxypalmitate	Lipid	Fatty Acid, Monohydroxy	0.98 (0.91, 1.06)	6.03E-01	8.56E-01	LC/MS Neg	HMDB31057	92836	100001579
2-Hydroxy-3-methylvalerate	Amino Acid	Leucine, Isoleucine and Valine Metabolism	0.98 (0.91, 1.06)	6.06E-01	8.59E-01	LC/MS Neg	HMDB00317	164623	100001541
Glycocholenate sulfate*	Lipid	Secondary Bile Acid Metabolism	1.02 (0.95, 1.10)	6.07E-01	8.59E-01	LC/MS Neg			100001989

4-Ethylphenylsulfate	Xenobiotics	Benzoate Metabolism	1.02 (0.95, 1.10)	6.09E-01	8.59E-01	LC/MS Neg	HMDB62551	20822574	100001756
Mannonate*	Xenobiotics	Food Component/Plant	1.02 (0.95, 1.10)	6.11E-01	8.61E-01	LC/MS Polar		3246006	100003695
Tauro-β-muricholate	Lipid	Primary Bile Acid Metabolism	0.98 (0.91, 1.06)	6.12E-01	8.61E-01	LC/MS Neg	HMDB00932	168408	100001250
SM (d18:1/19:0, d19:1/18:0)*	Lipid	Sphingomyelins	0.98 (0.91, 1.06)	6.16E-01	8.65E-01	LC/MS Pos Late			100015787
Laurylcarnitine (C12)	Lipid	Fatty Acid Metabolism (Acyl Carnitine, Medium Chain)	1.02 (0.95, 1.10)	6.17E-01	8.65E-01	LC/MS Pos Late	HMDB02250	10427569	100001392
I-Urobilinogen	Cofactors and Vitamins	Hemoglobin and Porphyrin Metabolism	0.98 (0.91, 1.06)	6.18E-01	8.65E-01	LC/MS Neg	HMDB04157	26818	100001226
2-Hydroxyphenylacetate sulfate	Amino Acid	Phenylalanine Metabolism	1.02 (0.95, 1.10)	6.19E-01	8.65E-01	LC/MS Neg			100006635
Glycocholate	Lipid	Primary Bile Acid Metabolism	0.98 (0.91, 1.06)	6.20E-01	8.66E-01	LC/MS Neg	HMDB00138	10140	342
4-Hydroxyphenylpyruvate	Amino Acid	Tyrosine Metabolism	0.98 (0.91, 1.06)	6.25E-01	8.67E-01	LC/MS Neg	HMDB00707	979	1141
Glycosyl-N-stearoyl-sphingosine (d18:1/18:0)	Lipid	Hexosylceramides (HCER)	0.98 (0.91, 1.06)	6.25E-01	8.67E-01	LC/MS Pos Late			100001882
γ-Glutamylisoleucine*	Peptide	Gamma-glutamyl Amino Acid	1.02 (0.94, 1.11)	6.25E-01	8.67E-01	LC/MS Pos Early	HMDB11170	14253342	100001485
Docosahexaenoate (DHA; 22:6n3)	Lipid	Long Chain Polyunsaturated Fatty Acid (n3 and n6)	0.98 (0.91, 1.06)	6.26E-01	8.67E-01	LC/MS Neg	HMDB02183	445580	100000665
3-Aminoisobutyrate	Nucleotide	Pyrimidine Metabolism, Thymine containing	0.98 (0.91, 1.06)	6.27E-01	8.67E-01	LC/MS Pos Early	HMDB03911	64956	1114
N-Stearoyltaurine	Lipid	Endocannabinoid	0.98 (0.92, 1.05)	6.28E-01	8.67E-01	LC/MS Neg		168274	100003240
Pyridoxal	Cofactors and Vitamins	Vitamin B6 Metabolism	1.02 (0.95, 1.09)	6.29E-01	8.67E-01	LC/MS Neg	HMDB01545	1050	491
2-Piperidinone	Xenobiotics	Food Component/Plant	0.98 (0.91, 1.06)	6.29E-01	8.67E-01	LC/MS Pos Early	HMDB11749	12665	100004295
3-Methylcrotonylglycine	Amino Acid	Leucine, Isoleucine and Valine Metabolism	1.02 (0.95, 1.09)	6.31E-01	8.68E-01	LC/MS Pos Early	HMDB00459	169485	100001149
Deoxycarnitine	Lipid	Carnitine Metabolism	0.98 (0.91, 1.06)	6.32E-01	8.68E-01	LC/MS Pos Early	HMDB01161	134	100001662
Dihydroferulic acid sulfate	Xenobiotics	Food Component/Plant	0.98 (0.91, 1.06)	6.33E-01	8.68E-01	LC/MS Polar	HMDB41724	187489	100020272
4-Methoxyphenol sulfate	Amino Acid	Tyrosine Metabolism	0.98 (0.91, 1.06)	6.42E-01	8.76E-01	LC/MS Neg			100006262
Lanthionine	Amino Acid	Methionine, Cysteine, SAM and Taurine Metabolism	0.98 (0.91, 1.06)	6.43E-01	8.76E-01	LC/MS Pos Early		98504	100003892
Acesulfame	Xenobiotics	Food Component/Plant	1.02 (0.95, 1.10)	6.44E-01	8.76E-01	LC/MS Neg	HMDB33585	36573	100006203
N-Acetyltheanine	Xenobiotics	Food Component/Plant	1.02 (0.95, 1.09)	6.48E-01	8.76E-01	LC/MS Polar			100020514
Ferulic acid 4-sulfate	Xenobiotics	Food Component/Plant	0.98 (0.91, 1.06)	6.48E-01	8.76E-01	LC/MS Neg	HMDB29200	6305574	100005389
Vanillate glucuronide	Xenobiotics	Food Component/Plant	1.02 (0.94, 1.10)	6.49E-01	8.76E-01	LC/MS Polar			100020606
Pentadecanoate (15:0)	Lipid	Long Chain Saturated Fatty Acid	0.98 (0.91, 1.06)	6.49E-01	8.76E-01	LC/MS Neg	HMDB00826	13849	980
δ-CEHC glucuronide	Cofactors and Vitamins	Tocopherol Metabolism	1.02 (0.94, 1.10)	6.50E-01	8.76E-01	LC/MS Neg			100020483
4-Hydroxy-2-oxoglutaric acid	Lipid	Fatty Acid, Dicarboxylate	1.02 (0.95, 1.09)	6.51E-01	8.76E-01	LC/MS Polar	HMDB02070	599	100002537
5α-Androstan-3α,17β-diol disulfate	Lipid	Androgenic Steroids	1.02 (0.94, 1.10)	6.52E-01	8.76E-01	LC/MS Neg			100002017
Isoeugenol sulfate	Xenobiotics	Food Component/Plant	1.02 (0.94, 1.10)	6.54E-01	8.76E-01	LC/MS Neg			100006089
Taurocholenate sulfate*	Lipid	Secondary Bile Acid Metabolism	1.02 (0.94, 1.10)	6.55E-01	8.76E-01	LC/MS Neg			100001990
N-Acetylcitrulline	Amino Acid	Urea cycle; Arginine and Proline Metabolism	1.02 (0.94, 1.10)	6.55E-01	8.76E-01	LC/MS Neg	HMDB00856	656979	100001577
4-Allylphenol sulfate	Xenobiotics	Food Component/Plant	1.02 (0.94, 1.10)	6.58E-01	8.76E-01	LC/MS Neg			100001868

Phenol glucuronide	Amino Acid	Tyrosine Metabolism	1.02 (0.95, 1.09)	6.58E-01	8.76E-01	LC/MS Neg	HMDB60014	87235	100006679
2-Oxoarginine*	Amino Acid	Urea cycle; Arginine and Proline Metabolism	1.02 (0.94, 1.10)	6.58E-01	8.76E-01	LC/MS Pos Early	HMDB04225	558	100002784
Palmitoleoylcarnitine (C16:1)*	Lipid	Fatty Acid Metabolism (Acyl Carnitine, Monounsaturated)	0.98 (0.91, 1.06)	6.58E-01	8.76E-01	LC/MS Pos Late		71464547	100009406
Dihomo-linolenoyl-choline	Lipid	Fatty Acid Metabolism (Acyl Choline)	0.98 (0.91, 1.06)	6.59E-01	8.76E-01	LC/MS Pos Late			100009335
Citramalate	Amino Acid	Glutamate Metabolism	1.02 (0.95, 1.09)	6.59E-01	8.76E-01	LC/MS Pos Early	HMDB00426	1081	100000998
3-Hydroxyisobutyrate	Amino Acid	Leucine, Isoleucine and Valine Metabolism	1.02 (0.94, 1.10)	6.59E-01	8.76E-01	LC/MS Polar	HMDB00336	87	111
N-Acetylvaline	Amino Acid	Leucine, Isoleucine and Valine Metabolism	0.98 (0.91, 1.06)	6.60E-01	8.76E-01	LC/MS Neg	HMDB11757	66789	1084
Arachidonate (20:4n6)	Lipid	Long Chain Polyunsaturated Fatty Acid (n3 and n6)	0.98 (0.91, 1.06)	6.65E-01	8.80E-01	LC/MS Neg	HMDB01043	444899	229
Umbelliferone sulfate	Xenobiotics	Food Component/Plant	0.98 (0.91, 1.06)	6.66E-01	8.80E-01	LC/MS Neg		129659	100006282
γ-CEHC	Cofactors and Vitamins	Tocopherol Metabolism	0.98 (0.91, 1.06)	6.67E-01	8.80E-01	LC/MS Neg	HMDB01931	133098	100002094
3-Hydroxyhexanoate	Lipid	Fatty Acid, Monohydroxy	0.98 (0.91, 1.06)	6.68E-01	8.80E-01	LC/MS Neg		151492	100006367
Alliin	Xenobiotics	Food Component/Plant	0.98 (0.91, 1.06)	6.69E-01	8.80E-01	LC/MS Pos Early	HMDB33592	87310	100003473
Tryptophan	Amino Acid	Tryptophan Metabolism	1.02 (0.94, 1.10)	6.70E-01	8.80E-01	LC/MS Pos Early	HMDB00929	6305	565
N1-Methyl-2-pyridone-5-carboxamide	Cofactors and Vitamins	Nicotinate and Nicotinamide Metabolism	0.98 (0.92, 1.06)	6.70E-01	8.80E-01	LC/MS Neg	HMDB04193	69698	100001468
4-Hydroxyphenylacetate	Amino Acid	Phenylalanine Metabolism	0.98 (0.91, 1.06)	6.77E-01	8.87E-01	LC/MS Neg	HMDB00020	127	144
Palmitate (16:0)	Lipid	Long Chain Saturated Fatty Acid	0.98 (0.91, 1.06)	6.80E-01	8.90E-01	LC/MS Neg	HMDB00220	985	424
3β,7α-Dihydroxy-5-cholestenoate	Lipid	Sterol	0.98 (0.92, 1.06)	6.82E-01	8.91E-01	LC/MS Neg		3081084	100001611
SM (d18:1/25:0, d19:0/24:1, d20:1/23:0, d19:1/24:0)*	Lipid	Sphingomyelins	0.98 (0.91, 1.06)	6.84E-01	8.92E-01	LC/MS Pos Late			100015792
3,7-Dimethylurate	Xenobiotics	Xanthine Metabolism	0.99 (0.92, 1.06)	6.84E-01	8.92E-01	LC/MS Neg	HMDB01982	83126	100001398
1-Oleoylglycerol (18:1)	Lipid	Monoacylglycerol	1.02 (0.94, 1.10)	6.88E-01	8.96E-01	LC/MS Neg	HMDB11567	5283468	100000924
Naringenin 7-glucuronide	Xenobiotics	Food Component/Plant	0.99 (0.92, 1.06)	6.90E-01	8.96E-01	LC/MS Neg			100010898
2'-O-Methyluridine	Nucleotide	Pyrimidine Metabolism, Uracil containing	0.98 (0.91, 1.06)	6.93E-01	8.99E-01	LC/MS Neg		102212	100010896
DihydroSM (18:0/22:0)*	Lipid	Dihydrosphingomyelins	1.01 (0.94, 1.09)	6.97E-01	9.00E-01	LC/MS Pos Late	HMDB12091		100009026
heme	Cofactors and Vitamins	Hemoglobin and Porphyrin Metabolism	1.02 (0.94, 1.10)	6.98E-01	9.00E-01	LC/MS Pos Late	HMDB03178	26945	100001386
(R)-3-Hydroxybutyrylcarnitine	Lipid	Fatty Acid Metabolism (Acyl Carnitine, Hydroxy)	1.02 (0.94, 1.10)	6.98E-01	9.00E-01	LC/MS Pos Early	HMDB13127	53481617	100003926
Desmethylnaproxen sulfate	Xenobiotics	Drug - Analgesics, Anesthetics	1.01 (0.94, 1.09)	6.99E-01	9.00E-01	LC/MS Neg		184679	100001788
Docosahexaenoylcarnitine (C22:6)*	Lipid	Fatty Acid Metabolism (Acyl Carnitine, Polyunsaturated)	0.99 (0.92, 1.06)	6.99E-01	9.00E-01	LC/MS Pos Late			100015845
Trans-urocanate	Amino Acid	Histidine Metabolism	1.01 (0.94, 1.09)	7.00E-01	9.00E-01	LC/MS Polar	HMDB00301	736715	537
α-Ketobutyrate	Amino Acid	Methionine, Cysteine, SAM and Taurine Metabolism	0.99 (0.91, 1.06)	7.05E-01	9.04E-01	LC/MS Polar	HMDB00005	58	796
Daidzein sulfate (2)	Xenobiotics	Food Component/Plant	0.99 (0.92, 1.06)	7.06E-01	9.04E-01	LC/MS Neg			100006363
Linoleoyl-linoleoyl-glycerol (18:2/18:2) [2]*	Lipid	Diacylglycerol	0.99 (0.92, 1.06)	7.06E-01	9.04E-01	LC/MS Pos Late			100010942
3-Hydroxyhippurate sulfate	Xenobiotics	Benzoate Metabolism	0.99 (0.91, 1.06)	7.08E-01	9.05E-01	LC/MS Neg		186339	100020410

4-Methylcatechol sulfate	Xenobiotics	Benzoate Metabolism	0.99 (0.91, 1.06)	7.10E-01	9.05E-01	LC/MS Neg			100004111
Palmitoyl ethanolamide	Lipid	Endocannabinoid	0.99 (0.92, 1.06)	7.10E-01	9.05E-01	LC/MS Neg	HMDB02100	4671	1489
11-Ketoetiocholanolone glucuronide	Lipid	Androgenic Steroids	0.99 (0.92, 1.06)	7.15E-01	9.08E-01	LC/MS Neg			100005402
Glycoursodeoxycholic acid sulfate (1)	Lipid	Secondary Bile Acid Metabolism	0.99 (0.92, 1.06)	7.15E-01	9.08E-01	LC/MS Neg			100020819
Myristoleate (14:1n5)	Lipid	Long Chain Monounsaturated Fatty Acid	0.99 (0.92, 1.06)	7.17E-01	9.08E-01	LC/MS Neg	HMDB02000	5281119	100001198
Trigonelline (N'-methylnicotinate)	Cofactors and Vitamins	Nicotinate and Nicotinamide Metabolism	1.01 (0.94, 1.09)	7.17E-01	9.08E-01	LC/MS Pos Early	HMDB00875	5570	100001092
N6-Methyllysine	Amino Acid	Lysine Metabolism	1.01 (0.94, 1.09)	7.19E-01	9.08E-01	LC/MS Pos Early	HMDB02038	164795	100003415
Glutamate	Amino Acid	Glutamate Metabolism	0.99 (0.91, 1.06)	7.20E-01	9.08E-01	LC/MS Pos Early	HMDB00148	611	561
1-Carboxyethylvaline	Amino Acid	Leucine, Isoleucine and Valine Metabolism	1.01 (0.94, 1.09)	7.20E-01	9.08E-01	LC/MS Neg			100020423
Orotate	Nucleotide	Pyrimidine Metabolism, Orotate containing	1.01 (0.94, 1.09)	7.25E-01	9.14E-01	LC/MS Polar	HMDB00226	967	445
1-Carboxyethylisoleucine	Amino Acid	Leucine, Isoleucine and Valine Metabolism	1.01 (0.94, 1.10)	7.27E-01	9.14E-01	LC/MS Neg			100020427
Tigloylglycine	Amino Acid	Leucine, Isoleucine and Valine Metabolism	0.99 (0.92, 1.06)	7.28E-01	9.14E-01	LC/MS Pos Early	HMDB00959	6441567	1161
5-Methyluridine (ribothymidine)	Nucleotide	Pyrimidine Metabolism, Uracil containing	1.01 (0.94, 1.09)	7.31E-01	9.15E-01	LC/MS Neg	HMDB00884	445408	100001446
3-Hydroxyoleoylcarnitine	Lipid	Fatty Acid Metabolism (Acyl Carnitine, Hydroxy)	0.99 (0.91, 1.06)	7.31E-01	9.15E-01	LC/MS Pos Late			100019801
1-Methylhistidine	Amino Acid	Histidine Metabolism	1.01 (0.94, 1.10)	7.32E-01	9.15E-01	LC/MS Neg	HMDB00001	92105	100001051
Kynurenate	Amino Acid	Tryptophan Metabolism	0.99 (0.92, 1.06)	7.33E-01	9.15E-01	LC/MS Neg	HMDB00715	3845	98
α-Tocopherol	Cofactors and Vitamins	Tocopherol Metabolism	0.99 (0.91, 1.07)	7.35E-01	9.17E-01	LC/MS Pos Late	HMDB01893	14985	1105
β-Hydroxyisovalerate	Amino Acid	Leucine, Isoleucine and Valine Metabolism	1.01 (0.94, 1.09)	7.37E-01	9.18E-01	LC/MS Neg	HMDB00754	69362	1442
Tetradecadienedioate (C14:2-DC)*	Lipid	Fatty Acid, Dicarboxylate	0.99 (0.92, 1.06)	7.39E-01	9.19E-01	LC/MS Neg			100021100
Tauroursodeoxycholate	Lipid	Secondary Bile Acid Metabolism	0.99 (0.91, 1.07)	7.41E-01	9.20E-01	LC/MS Neg	HMDB00874	9848818	100002912
Caffeine	Xenobiotics	Xanthine Metabolism	0.99 (0.92, 1.06)	7.42E-01	9.20E-01	LC/MS Pos Early	HMDB01847	2519	849
SM (d18:1/18:1, d18:2/18:0)	Lipid	Sphingomyelins	0.99 (0.91, 1.07)	7.43E-01	9.20E-01	LC/MS Pos Late	HMDB12101	6443882	100002106
Salicyluric glucuronide*	Xenobiotics	Drug - Analgesics, Anesthetics	1.01 (0.94, 1.09)	7.48E-01	9.25E-01	LC/MS Neg			100001332
5-Methylthioadenosine (MTA)	Amino Acid	Polyamine Metabolism	1.01 (0.94, 1.09)	7.50E-01	9.25E-01	LC/MS Pos Early	HMDB01173	439176	212
Methylmalonate (MMA)	Lipid	Fatty Acid Metabolism (also BCAA Metabolism)	1.01 (0.94, 1.09)	7.50E-01	9.25E-01	LC/MS Polar	HMDB00202	487	418
Hypoxanthine	Nucleotide	Purine Metabolism, (Hypo)Xanthine/Inosine containing	1.01 (0.94, 1.09)	7.51E-01	9.25E-01	LC/MS Neg	HMDB00157	790	171
Picolinate	Amino Acid	Tryptophan Metabolism	0.99 (0.92, 1.06)	7.53E-01	9.26E-01	LC/MS Pos Early	HMDB02243	1018	1022
3-Hydroxydecanoate	Lipid	Fatty Acid, Monohydroxy	0.99 (0.92, 1.06)	7.56E-01	9.28E-01	LC/MS Neg	HMDB02203	26612	100000997
Pyruvate	Carbohydrate	Glycolysis, Gluconeogenesis, and Pyruvate Metabolism	0.99 (0.92, 1.06)	7.59E-01	9.30E-01	LC/MS Polar	HMDB00243	1060	823
Sulfate of piperine metabolite C18H21NO3 (1)*	Xenobiotics	Food Component/Plant	1.01 (0.94, 1.09)	7.60E-01	9.30E-01	LC/MS Neg			100020502
Decadienedioic acid (C10:2-DC)**	Lipid	Fatty Acid, Dicarboxylate	0.99 (0.92, 1.07)	7.68E-01	9.34E-01	LC/MS Neg			100021711

N2-Acetyl,N6-methyllysine	Amino Acid	Lysine Metabolism	1.01 (0.94, 1.09)	7.68E-01	9.34E-01	LC/MS Pos Early			100020546
N-Formylphenylalanine	Amino Acid	Tyrosine Metabolism	0.99 (0.92, 1.06)	7.68E-01	9.34E-01	LC/MS Neg		759256	100006056
γ-Carboxyglutamate	Amino Acid	Glutamate Metabolism	0.99 (0.92, 1.07)	7.68E-01	9.34E-01	LC/MS Pos Early	HMDB41900	40772	100002679
2-Hydroxy-4-(methylthio)butanoic acid	Amino Acid	Methionine, Cysteine, SAM and Taurine Metabolism	0.99 (0.92, 1.06)	7.69E-01	9.34E-01	LC/MS Polar			100021372
Fibrinopeptide A, des-ala(1)*	Peptide	Fibrinogen Cleavage Peptide	1.01 (0.94, 1.09)	7.70E-01	9.34E-01	LC/MS Neg			100001323
5-Oxoproline	Amino Acid	Glutathione Metabolism	0.99 (0.92, 1.07)	7.71E-01	9.34E-01	LC/MS Neg	HMDB00267	7405	1021
Sarcosine	Amino Acid	Glycine, Serine and Threonine Metabolism	1.01 (0.94, 1.09)	7.71E-01	9.34E-01	LC/MS Pos Early	HMDB00271	1088	1023
γ-Glutamyltryptophan	Peptide	Gamma-glutamyl Amino Acid	0.99 (0.92, 1.07)	7.73E-01	9.35E-01	LC/MS Neg	HMDB29160	3989307	100001295
10-Heptadecenoate (17:1n7)	Lipid	Long Chain Monounsaturated Fatty Acid	0.99 (0.92, 1.07)	7.75E-01	9.35E-01	LC/MS Neg	HMDB60038	5312435	100001278
Hexadecanedioate (C16-DC)	Lipid	Fatty Acid, Dicarboxylate	0.99 (0.92, 1.07)	7.76E-01	9.35E-01	LC/MS Neg	HMDB00672	10459	100001614
Myristoylcarnitine (C14)	Lipid	Fatty Acid Metabolism (Acyl Carnitine, Long Chain Saturated)	0.99 (0.92, 1.07)	7.79E-01	9.37E-01	LC/MS Pos Late	HMDB05066	6426854	100001270
N-Methylhydroxyproline**	Amino Acid	Urea cycle; Arginine and Proline Metabolism	0.99 (0.91, 1.07)	7.81E-01	9.38E-01	LC/MS Pos Early		9860390	100020215
Naproxen	Xenobiotics	Drug - Analgesics, Anesthetics	1.01 (0.94, 1.08)	7.82E-01	9.39E-01	LC/MS Neg	HMDB01923	156391	1384
Glutarate (C5-DC)	Lipid	Fatty Acid, Dicarboxylate	0.99 (0.92, 1.07)	7.85E-01	9.39E-01	LC/MS Polar	HMDB00661	743	339
4-Vinylguaiacol sulfate	Xenobiotics	Food Component/Plant	0.99 (0.92, 1.07)	7.85E-01	9.39E-01	LC/MS Neg			100006126
Acisoga	Amino Acid	Polyamine Metabolism	0.99 (0.92, 1.07)	7.85E-01	9.39E-01	LC/MS Neg		129397	100004541
Taurocholate	Lipid	Primary Bile Acid Metabolism	1.01 (0.94, 1.09)	7.87E-01	9.40E-01	LC/MS Neg	HMDB00036	6675	1648
Methionine sulfone	Amino Acid	Methionine, Cysteine, SAM and Taurine Metabolism	0.99 (0.91, 1.07)	7.93E-01	9.43E-01	LC/MS Pos Early		69961	100004635
Adrenoylcarnitine (C22:4)*	Lipid	Fatty Acid Metabolism (Acyl Carnitine, Polyunsaturated)	1.01 (0.94, 1.09)	7.96E-01	9.43E-01	LC/MS Pos Late			100015850
Linoleoyl-linoleoyl-glycerol (18:2/18:2) [1]*	Lipid	Diacylglycerol	0.99 (0.92, 1.07)	7.96E-01	9.43E-01	LC/MS Pos Late			100010941
γ-Glutamyltyrosine	Peptide	Gamma-glutamyl Amino Acid	0.99 (0.91, 1.07)	7.96E-01	9.43E-01	LC/MS Pos Early	HMDB11741	94340	1102
β-Hydroxyisovaleroylcarnitine	Amino Acid	Leucine, Isoleucine and Valine Metabolism	0.99 (0.92, 1.07)	7.97E-01	9.43E-01	LC/MS Pos Early			100001594
3-Methoxytyrosine	Amino Acid	Tyrosine Metabolism	0.99 (0.92, 1.06)	7.98E-01	9.43E-01	LC/MS Pos Early	HMDB01434	1670	1342
2'-Deoxyuridine	Nucleotide	Pyrimidine Metabolism, Uracil containing	0.99 (0.92, 1.06)	7.99E-01	9.43E-01	LC/MS Polar	HMDB00012	13712	536
Bilirubin (E,E)*	Cofactors and Vitamins	Hemoglobin and Porphyrin Metabolism	0.99 (0.92, 1.06)	7.99E-01	9.43E-01	LC/MS Neg		5315454	100001950
Prolylglycine	Peptide	Dipeptide	1.01 (0.94, 1.09)	8.00E-01	9.43E-01	LC/MS Pos Early	HMDB11178	6426709	100003674
Laurate (12:0)	Lipid	Medium Chain Fatty Acid	1.01 (0.94, 1.08)	8.01E-01	9.43E-01	LC/MS Neg	HMDB00638	3893	181
N6,N6,N6-Trimethyllysine	Amino Acid	Lysine Metabolism	0.99 (0.92, 1.07)	8.02E-01	9.43E-01	LC/MS Pos Early	HMDB01325	440120	189
Octadecenedioylcarnitine (C18:1-DC)*	Lipid	Fatty Acid Metabolism (Acyl Carnitine, Dicarboxylate)	0.99 (0.91, 1.07)	8.04E-01	9.43E-01	LC/MS Pos Late			100005996
Palmitoleoyl-linoleoyl-glycerol (16:1/18:2) [1]*	Lipid	Diacylglycerol	1.01 (0.94, 1.09)	8.07E-01	9.43E-01	LC/MS Pos Late			100010930
Bilirubin (Z,Z)	Cofactors and Vitamins	Hemoglobin and Porphyrin Metabolism	0.99 (0.92, 1.07)	8.10E-01	9.43E-01	LC/MS Pos Late	HMDB00054	5280352	1090
Vanillactate	Amino Acid	Tyrosine Metabolism	0.99 (0.92, 1.07)	8.10E-01	9.43E-01	LC/MS Neg	HMDB00913	160637	100006129

Dihomo-linolenate (20:3n3 or n6)	Lipid	Long Chain Polyunsaturated Fatty Acid (n3 and n6)	0.99 (0.92, 1.07)	8.10E-01	9.43E-01	LC/MS Neg	HMDB02925	5280581	100001739
5-Acetylamino-6-formylamino-3-methyluracil	Xenobiotics	Xanthine Metabolism	1.01 (0.93, 1.09)	8.11E-01	9.43E-01	LC/MS Polar	HMDB11105	108214	100001402
3-Hydroxyarachidate*	Lipid	Fatty Acid, Monohydroxy	1.01 (0.94, 1.09)	8.11E-01	9.43E-01	LC/MS Neg		5282918	100019893
Carboxyibuprofen glucuronide*	Xenobiotics	Drug - Analgesics, Anesthetics	1.01 (0.94, 1.08)	8.11E-01	9.43E-01	LC/MS Polar	HMDB60564		100020212
5,6-Dihydrouridine	Nucleotide	Pyrimidine Metabolism, Uracil containing	1.01 (0.93, 1.09)	8.12E-01	9.43E-01	LC/MS Neg		94312	100001425
N-Acetylalliin	Xenobiotics	Food Component/Plant	0.99 (0.92, 1.07)	8.13E-01	9.43E-01	LC/MS Pos Early			100005367
Palmitoloelycholine	Lipid	Fatty Acid Metabolism (Acyl Choline)	0.99 (0.92, 1.07)	8.14E-01	9.43E-01	LC/MS Pos Late			100009334
Urate	Nucleotide	Purine Metabolism, (Hypo)Xanthine/Inosine containing	1.01 (0.94, 1.09)	8.16E-01	9.44E-01	LC/MS Neg	HMDB00289	1175	1134
Vanillic alcohol sulfate	Amino Acid	Tyrosine Metabolism	0.99 (0.92, 1.07)	8.18E-01	9.45E-01	LC/MS Neg			100006125
1H-Indole-7-acetic acid	Xenobiotics	Bacterial/Fungal	0.99 (0.92, 1.07)	8.21E-01	9.47E-01	LC/MS Neg			100004601
cis-4-Decenoylcarnitine (C10:1)	Lipid	Fatty Acid Metabolism (Acyl Carnitine, Monounsaturated)	1.01 (0.93, 1.09)	8.22E-01	9.47E-01	LC/MS Pos Late			100002259
Pseudouridine	Nucleotide	Pyrimidine Metabolism, Uracil containing	1.01 (0.93, 1.09)	8.23E-01	9.47E-01	LC/MS Neg	HMDB00767	15047	821
7-α-Hydroxy-3-oxo-4-cholestenoate (7-Hoca)	Lipid	Sterol	0.99 (0.92, 1.07)	8.23E-01	9.47E-01	LC/MS Neg	HMDB12458	3081085	100001609
N-Acetylarginine	Amino Acid	Urea cycle; Arginine and Proline Metabolism	0.99 (0.92, 1.07)	8.26E-01	9.47E-01	LC/MS Pos Early	HMDB04620	67427	100001266
Cys-gly, oxidized	Amino Acid	Glutathione Metabolism	1.01 (0.94, 1.09)	8.28E-01	9.47E-01	LC/MS Neg		333293	1224
5α-Androstan-3β,17α-diol disulfate	Lipid	Androgenic Steroids	1.01 (0.94, 1.09)	8.29E-01	9.47E-01	LC/MS Neg			100002021
Llithocholate sulfate (1)	Lipid	Secondary Bile Acid Metabolism	1.01 (0.94, 1.08)	8.30E-01	9.47E-01	LC/MS Neg			100020823
Dimethyl sulfone	Xenobiotics	Chemical	1.01 (0.93, 1.09)	8.30E-01	9.47E-01	LC/MS Pos Early	HMDB04983	6213	100004284
Propionylcarnitine (C3)	Lipid	Fatty Acid Metabolism (also BCAA Metabolism)	1.01 (0.93, 1.09)	8.30E-01	9.47E-01	LC/MS Pos Early	HMDB00824	107738	100001162
PI (16:0/20:4)*	Lipid	Phosphatidylinositol (PI)	1.01 (0.93, 1.09)	8.34E-01	9.49E-01	LC/MS Polar	HMDB09789		100008993
N-Acetylleucine	Amino Acid	Leucine, Isoleucine and Valine Metabolism	1.01 (0.94, 1.09)	8.35E-01	9.50E-01	LC/MS Neg	HMDB11756	70912	1082
Lactate	Carbohydrate	Glycolysis, Gluconeogenesis, and Pyruvate Metabolism	1.01 (0.94, 1.08)	8.37E-01	9.51E-01	LC/MS Neg	HMDB00190	612	482
Hydroxy-N6,N6,N6-trimethyllysine*	Amino Acid	Lysine Metabolism	1.01 (0.93, 1.10)	8.39E-01	9.51E-01	LC/MS Pos Early			100021123
γ-Glutamylleucine	Peptide	Gamma-glutamyl Amino Acid	1.01 (0.93, 1.09)	8.39E-01	9.51E-01	LC/MS Neg	HMDB11171	151023	1268
Piperine	Xenobiotics	Food Component/Plant	0.99 (0.92, 1.07)	8.42E-01	9.51E-01	LC/MS Pos Late	HMDB29377	638024	100001267
3-Hydroxyadipate*	Lipid	Fatty Acid, Dicarboxylate	0.99 (0.92, 1.07)	8.43E-01	9.51E-01	LC/MS Polar	HMDB00345	151913	100004396
Spermine	Amino Acid	Polyamine Metabolism	0.99 (0.92, 1.07)	8.43E-01	9.51E-01	LC/MS Pos Early	HMDB01256	1103	507
Orotidine	Nucleotide	Pyrimidine Metabolism, Orotate containing	0.99 (0.91, 1.08)	8.44E-01	9.51E-01	LC/MS Polar	HMDB00788	92751	100001416
2,3-Dihydroxyisovalerate	Xenobiotics	Food Component/Plant	1.01 (0.93, 1.09)	8.45E-01	9.51E-01	LC/MS Polar	HMDB12141	677	100002417
S-Carboxyethylcysteine	Amino Acid	Methionine, Cysteine, SAM and Taurine Metabolism	0.99 (0.92, 1.07)	8.47E-01	9.52E-01	LC/MS Pos Early			100021530
Myristate (14:0)	Lipid	Long Chain Saturated Fatty Acid	0.99 (0.92, 1.07)	8.52E-01	9.56E-01	LC/MS Neg	HMDB00806	11005	519

5-Dodecenoate (12:1n7)	Lipid	Medium Chain Fatty Acid	1.01 (0.94, 1.08)	8.53E-01	9.56E-01	LC/MS Neg	HMDB00529	5312378	100001232
Oleoyl-linOleoyl-glycerol (18:1/18:2) [2]	Lipid	Diacylglycerol	0.99 (0.92, 1.07)	8.54E-01	9.56E-01	LC/MS Pos Late	HMDB07219		100002990
γ-tocopherol/β-tocopherol	Cofactors and Vitamins	Tocopherol Metabolism	0.99 (0.92, 1.07)	8.57E-01	9.59E-01	LC/MS Pos Late			100008998
N6,N6-Dimethyllysine	Amino Acid	Lysine Metabolism	0.99 (0.92, 1.07)	8.61E-01	9.61E-01	LC/MS Pos Early	HMDB13287	193344	100020417
2S,3R-Dihydroxybutyrate	Lipid	Fatty Acid, Dihydroxy	0.99 (0.92, 1.07)	8.63E-01	9.61E-01	LC/MS Polar	HMDB02453	10964471	100002284
Taurochenodeoxycholate	Lipid	Primary Bile Acid Metabolism	1.01 (0.93, 1.09)	8.63E-01	9.61E-01	LC/MS Neg	HMDB00951	387316	1629
Nervonoylcarnitine (C24:1)*	Lipid	Fatty Acid Metabolism (Acyl Carnitine, Monounsaturated)	0.99 (0.92, 1.07)	8.64E-01	9.61E-01	LC/MS Pos Late			100015846
N1-Methylinosine	Nucleotide	Purine Metabolism, (Hypo)Xanthine/Inosine containing	1.01 (0.93, 1.09)	8.65E-01	9.61E-01	LC/MS Pos Early	HMDB02721	65095	100001409
Cysteinylglycine disulfide*	Amino Acid	Glutathione Metabolism	1.01 (0.93, 1.09)	8.67E-01	9.61E-01	LC/MS Pos Early	HMDB00709		100002466
Linoleoyl-arachidonoyl-glycerol (18:2/20:4) [2]*	Lipid	Diacylglycerol	0.99 (0.92, 1.07)	8.68E-01	9.61E-01	LC/MS Pos Late			100010923
Cysteine s-sulfate	Amino Acid	Methionine, Cysteine, SAM and Taurine Metabolism	1.01 (0.93, 1.09)	8.68E-01	9.61E-01	LC/MS Polar	HMDB00731	115015	100000808
Isovalerylcarnitine (C5)	Amino Acid	Leucine, Isoleucine and Valine Metabolism	1.01 (0.93, 1.08)	8.69E-01	9.61E-01	LC/MS Pos Early	HMDB00688	6426851	100001393
10-Undecenoate (11:1n1)	Lipid	Medium Chain Fatty Acid	0.99 (0.92, 1.07)	8.70E-01	9.61E-01	LC/MS Neg	HMDB33724	14891	100001197
γ-Glutamyl-α-lysine	Peptide	Gamma-glutamyl Amino Acid	0.99 (0.92, 1.08)	8.71E-01	9.61E-01	LC/MS Pos Early		65254	100010901
3-Hydroxydodecanedioate*	Lipid	Fatty Acid, Dicarboxylate	0.99 (0.92, 1.07)	8.75E-01	9.63E-01	LC/MS Neg	HMDB00413	16663321	100001454
LysoPC (16:1)*	Lipid	Lysophospholipid	0.99 (0.92, 1.07)	8.76E-01	9.63E-01	LC/MS Neg	HMDB10383		100001561
Octadecanedioylcarnitine (C18-DC)*	Lipid	Fatty Acid Metabolism (Acyl Carnitine, Dicarboxylate)	1.01 (0.93, 1.09)	8.81E-01	9.64E-01	LC/MS Pos Late			100005998
Indolelactate	Amino Acid	Tryptophan Metabolism	1.01 (0.93, 1.09)	8.82E-01	9.64E-01	LC/MS Neg	HMDB00671	92904	100000463
PC (16:0/16:1)*	Lipid	Phosphatidylcholine (PC)	1.01 (0.93, 1.09)	8.82E-01	9.64E-01	LC/MS Pos Late			100008984
Linoleoyl-arachidonoyl-glycerol (18:2/20:4) [1]*	Lipid	Diacylglycerol	0.99 (0.92, 1.07)	8.83E-01	9.64E-01	LC/MS Pos Late			100010922
Oleoyl-arachidonoyl-glycerol (18:1/20:4) [1]*	Lipid	Diacylglycerol	0.99 (0.92, 1.07)	8.86E-01	9.64E-01	LC/MS Pos Late			100010936
Heptenedioate (C7:1-DC)*	Lipid	Fatty Acid, Dicarboxylate	1.01 (0.93, 1.08)	8.87E-01	9.64E-01	LC/MS Polar			100019981
2-Hydroxybutyrate/2-hydroxyisobutyrate	Amino Acid	Glutathione Metabolism	1.01 (0.93, 1.09)	8.89E-01	9.64E-01	LC/MS Polar			100008928
Valine	Amino Acid	Leucine, Isoleucine and Valine Metabolism	1.01 (0.93, 1.08)	8.90E-01	9.64E-01	LC/MS Neg	HMDB00883	6287	566
1-Methyl-5-imidazoleacetate	Amino Acid	Histidine Metabolism	1.01 (0.93, 1.08)	8.90E-01	9.64E-01	LC/MS Pos Early	HMDB04988	6451814	100002910
N-Palmitoylserine	Lipid	Endocannabinoid	0.99 (0.92, 1.07)	8.90E-01	9.64E-01	LC/MS Neg		6453686	100015640
Leucine	Amino Acid	Leucine, Isoleucine and Valine Metabolism	1.01 (0.93, 1.09)	8.91E-01	9.64E-01	LC/MS Pos Early		6106	397
Myristoleoylcarnitine (C14:1)*	Lipid	Fatty Acid Metabolism (Acyl Carnitine, Monounsaturated)	0.99 (0.92, 1.07)	8.91E-01	9.64E-01	LC/MS Pos Late		90659872	100006051
Ectoine	Xenobiotics	Chemical	1.00 (0.92, 1.07)	8.96E-01	9.64E-01	LC/MS Pos Early		126041	100001635
1-Carboxyethyltyrosine	Amino Acid	Tyrosine Metabolism	1.00 (0.92, 1.07)	8.96E-01	9.64E-01	LC/MS Neg			100020421
γ-Glutamylphenylalanine	Peptide	Gamma-glutamyl Amino Acid	0.99 (0.92, 1.08)	8.97E-01	9.64E-01	LC/MS Neg	HMDB00594	111299	100000491
Phenylpyruvate	Amino Acid	Phenylalanine Metabolism	1.01 (0.93, 1.09)	8.97E-01	9.64E-01	LC/MS Neg	HMDB00205	997	241

1,6-Anhydroglucose	Xenobiotics	Food Component/Plant	1.00 (0.93, 1.07)	8.98E-01	9.64E-01	LC/MS Polar	HMDB00640	2724705	100000939
17α-Hydroxypregnanolone glucuronide	Lipid	Pregnenolone Steroids	1.01 (0.93, 1.09)	8.99E-01	9.64E-01	LC/MS Neg			100005418
Palmitoleate (16:1n7)	Lipid	Long Chain Monounsaturated Fatty Acid	1.00 (0.93, 1.08)	9.00E-01	9.64E-01	LC/MS Neg	HMDB03229	445638	452
N6-Succinyladenosine	Nucleotide	Purine Metabolism, Adenine containing	0.99 (0.92, 1.08)	9.01E-01	9.64E-01	LC/MS Neg	HMDB00912	165243	100001664
Argininate*	Amino Acid	Urea cycle; Arginine and Proline Metabolism	1.00 (0.92, 1.07)	9.03E-01	9.64E-01	LC/MS Pos Early	HMDB03148	160437	100002769
N2,N5-Diacetylornithine	Amino Acid	Urea cycle; Arginine and Proline Metabolism	1.00 (0.93, 1.09)	9.04E-01	9.64E-01	LC/MS Neg		65977	100004575
Hydroquinone sulfate	Xenobiotics	Drug - Topical Agents	1.00 (0.93, 1.08)	9.06E-01	9.64E-01	LC/MS Neg	HMDB02434	161220	100001604
cis-3,4-Methyleneheptanoate	Lipid	Fatty Acid, Branched	1.00 (0.93, 1.07)	9.06E-01	9.64E-01	LC/MS Neg			100021727
N2-Acetyllysine	Amino Acid	Lysine Metabolism	1.00 (0.92, 1.07)	9.07E-01	9.64E-01	LC/MS Polar	HMDB00446	92907	100001721
Docosadienoate (22:2n6)	Lipid	Long Chain Polyunsaturated Fatty Acid (n3 and n6)	1.00 (0.92, 1.07)	9.10E-01	9.64E-01	LC/MS Neg	HMDB61714	5282807	100001182
2-Methoxyhydroquinone sulfate (2)	Xenobiotics	Benzoate Metabolism	1.00 (0.94, 1.08)	9.10E-01	9.64E-01	LC/MS Neg			100021840
Methyl-4-hydroxybenzoate sulfate	Xenobiotics	Benzoate Metabolism	1.00 (0.92, 1.07)	9.11E-01	9.64E-01	LC/MS Neg			100006116
4-Vinylphenol sulfate	Xenobiotics	Benzoate Metabolism	1.00 (0.93, 1.07)	9.12E-01	9.64E-01	LC/MS Neg	HMDB62775	6426766	100001755
3-Methylglutarylcarnitine (2)	Amino Acid	Leucine, Isoleucine and Valine Metabolism	1.00 (0.93, 1.08)	9.14E-01	9.64E-01	LC/MS Pos Early	HMDB00552	128145	100005850
γ-CEHC glucuronide*	Cofactors and Vitamins	Tocopherol Metabolism	1.00 (0.93, 1.08)	9.14E-01	9.64E-01	LC/MS Neg			100004243
EDTA	Xenobiotics	Chemical	1.00 (0.93, 1.09)	9.15E-01	9.64E-01	LC/MS Neg	HMDB15109	6049	100001002
1-Palmitoleoylglycerol (16:1)*	Lipid	Monoacylglycerol	1.00 (0.92, 1.07)	9.16E-01	9.64E-01	LC/MS Neg			100008952
α-Ketoglutarate	Energy	TCA Cycle	1.00 (0.92, 1.08)	9.17E-01	9.64E-01	LC/MS Polar	HMDB00208	51	93
Lysoglycerol (20:4)	Lipid	Monoacylglycerol	1.00 (0.93, 1.08)	9.17E-01	9.64E-01	LC/MS Neg	HMDB11549	5282281	100001433
Pipecolate	Amino Acid	Lysine Metabolism	1.00 (0.93, 1.08)	9.19E-01	9.64E-01	LC/MS Neg	HMDB00070	849	1025
Dihomo-linoleate (20:2n6)	Lipid	Long Chain Polyunsaturated Fatty Acid (n3 and n6)	1.00 (0.92, 1.07)	9.20E-01	9.64E-01	LC/MS Neg	HMDB05060	6439848	1231
1,3,7-Trimethylurate	Xenobiotics	Xanthine Metabolism	1.00 (0.92, 1.08)	9.21E-01	9.64E-01	LC/MS Neg	HMDB02123	79437	100001397
Uridine	Nucleotide	Pyrimidine Metabolism, Uracil containing	1.00 (0.92, 1.07)	9.23E-01	9.64E-01	LC/MS Neg	HMDB00296	6029	535
Sulfate of piperine metabolite C18H21NO3 (3)*	Xenobiotics	Food Component/Plant	1.00 (0.93, 1.08)	9.25E-01	9.64E-01	LC/MS Neg			100020504
3-Methylhistidine	Amino Acid	Histidine Metabolism	1.00 (0.93, 1.07)	9.25E-01	9.64E-01	LC/MS Neg	HMDB00479	64969	100000042
Tridecenedioate (C13:1-DC)*	Lipid	Fatty Acid, Dicarboxylate	1.00 (0.93, 1.08)	9.25E-01	9.64E-01	LC/MS Neg			100002199
3-Hydroxylaurate	Lipid	Fatty Acid, Monohydroxy	1.00 (0.93, 1.07)	9.26E-01	9.64E-01	LC/MS Neg	HMDB00387	94216	100001112
Sulfate*	Xenobiotics	Chemical	1.00 (0.92, 1.07)	9.26E-01	9.64E-01	LC/MS Neg	HMDB01448	1118	100002528
Sucrose	Carbohydrate	Disaccharides and Oligosaccharides	1.00 (0.93, 1.08)	9.26E-01	9.64E-01	LC/MS Polar	HMDB00258	5988	935
3-Indoleglyoxylic acid	Xenobiotics	Food Component/Plant	1.00 (0.93, 1.07)	9.36E-01	9.73E-01	LC/MS Neg		73863	100020372
Spermidine	Amino Acid	Polyamine Metabolism	1.00 (0.93, 1.07)	9.45E-01	9.81E-01	LC/MS Pos Early	HMDB01257	1102	50
Lysine	Amino Acid	Lysine Metabolism	1.00 (0.93, 1.08)	9.48E-01	9.83E-01	LC/MS Pos Early	HMDB00182	5962	407
1-Carboxyethylphenylalanine	Amino Acid	Phenylalanine Metabolism	1.00 (0.93, 1.08)	9.51E-01	9.84E-01	LC/MS Neg			100020419

2-Aminoheptanoate	Lipid	Fatty Acid, Amino	1.00 (0.93, 1.08)	9.51E-01	9.84E-01	LC/MS Pos Early		227939	100004542
PI (16:0/18:2)	Lipid	Phosphatidylinositol (PI)	1.00 (0.92, 1.08)	9.52E-01	9.84E-01	LC/MS Polar	HMDB09784		1528
Docosapentaenoate (n6 DPA; 22:5n6)	Lipid	Long Chain Polyunsaturated Fatty Acid (n3 and n6)	1.00 (0.93, 1.08)	9.54E-01	9.84E-01	LC/MS Neg	HMDB01976	6441454	100001580
PI (16:0/18:1)*	Lipid	Phosphatidylinositol (PI)	1.00 (0.93, 1.08)	9.54E-01	9.84E-01	LC/MS Polar	HMDB09783		100009066
Butyrylcarnitine (C4)	Lipid	Fatty Acid Metabolism (also BCAA Metabolism)	1.00 (0.93, 1.08)	9.59E-01	9.85E-01	LC/MS Pos Early	HMDB02013	439829	100001054
Oleoyl-linOleoyl-glycerol (18:1/18:2) [1]	Lipid	Diacylglycerol	1.00 (0.93, 1.08)	9.60E-01	9.85E-01	LC/MS Pos Late	HMDB07219		100002989
Caprate (10:0)	Lipid	Medium Chain Fatty Acid	1.00 (0.93, 1.07)	9.60E-01	9.85E-01	LC/MS Neg	HMDB00511	2969	888
Adenosine 5'-monophosphate (AMP)	Nucleotide	Purine Metabolism, Adenine containing	1.00 (0.93, 1.08)	9.61E-01	9.85E-01	LC/MS Neg	HMDB00045	6083	209
Pro-hydroxy-pro	Amino Acid	Urea cycle; Arginine and Proline Metabolism	1.00 (0.92, 1.08)	9.62E-01	9.85E-01	LC/MS Pos Early	HMDB06695	11673055	100001167
3-Hydroxymyristate	Lipid	Fatty Acid, Monohydroxy	1.00 (0.93, 1.08)	9.64E-01	9.85E-01	LC/MS Neg		16064	100000882
Oleoyl-arachidonoyl-glycerol (18:1/20:4) [2]*	Lipid	Diacylglycerol	1.00 (0.93, 1.08)	9.64E-01	9.85E-01	LC/MS Pos Late			100010937
Indoleacetylglutamine	Amino Acid	Tryptophan Metabolism	1.00 (0.92, 1.08)	9.65E-01	9.85E-01	LC/MS Neg	HMDB13240	25200879	100001731
2,6-Dihydroxybenzoic acid	Xenobiotics	Drug - Topical Agents	1.00 (0.93, 1.08)	9.68E-01	9.87E-01	LC/MS Neg	HMDB13676	9338	100020837
Isoleucine	Amino Acid	Leucine, Isoleucine and Valine Metabolism	1.00 (0.93, 1.08)	9.70E-01	9.88E-01	LC/MS Pos Early	HMDB00172	6306	376
16-Hydroxypalmitate	Lipid	Fatty Acid, Monohydroxy	1.00 (0.93, 1.07)	9.70E-01	9.88E-01	LC/MS Neg	HMDB06294	10466	100002953
N-Acetylputrescine	Amino Acid	Polyamine Metabolism	1.00 (0.92, 1.08)	9.74E-01	9.90E-01	LC/MS Pos Early	HMDB02064	122356	192
PC (18:0/22:6)	Lipid	Phosphatidylcholine (PC)	1.00 (0.93, 1.08)	9.75E-01	9.90E-01	LC/MS Pos Late			100008916
3-Hydroxybutyrate (BHBA)	Lipid	Ketone Bodies	1.00 (0.93, 1.08)	9.79E-01	9.91E-01	LC/MS Pos Early	HMDB00357	441	254
PC (14:0/16:0)	Lipid	Phosphatidylcholine (PC)	1.00 (0.93, 1.08)	9.79E-01	9.91E-01	LC/MS Pos Late	HMDB07869	129657	100000672
N6-Acetyllysine	Amino Acid	Lysine Metabolism	1.00 (0.93, 1.08)	9.80E-01	9.91E-01	LC/MS Neg	HMDB00206	92832	100001734
Glycochenodeoxycholate 3-sulfate	Lipid	Primary Bile Acid Metabolism	1.00 (0.92, 1.08)	9.83E-01	9.93E-01	LC/MS Neg			100006641
cis-3,4-Methyleneheptanoylcarnitine	Lipid	Fatty Acid Metabolism (Acyl Carnitine, Hydroxy)	1.00 (0.93, 1.08)	9.86E-01	9.94E-01	LC/MS Neg			100021728
Deoxycholic acid 12-sulfate*	Lipid	Secondary Bile Acid Metabolism	1.00 (0.93, 1.08)	9.87E-01	9.94E-01	LC/MS Neg			100021712
Glycerol	Lipid	Glycerolipid Metabolism	1.00 (0.93, 1.08)	9.89E-01	9.94E-01	LC/MS Neg	HMDB00131	753	1254
Hexadecenedioate (C16:1-DC)*	Lipid	Fatty Acid, Dicarboxylate	1.00 (0.93, 1.08)	9.90E-01	9.94E-01	LC/MS Neg			100019975
N,N-Dimethylalanine	Amino Acid	Alanine and Aspartate Metabolism	1.00 (0.93, 1.08)	9.92E-01	9.94E-01	LC/MS Pos Early		5488191	100020371
2-Ooylglycerol (18:1)	Lipid	Monoacylglycerol	1.00 (0.93, 1.08)	9.93E-01	9.94E-01	LC/MS Neg	HMDB11537	5319879	100000943
(S)-3-Hydroxybutyrylcarnitine	Lipid	Fatty Acid Metabolism (Acyl Carnitine, Hydroxy)	1.00 (0.93, 1.08)	9.93E-01	9.94E-01	LC/MS Pos Early			100009271
Histidine betaine (hercynine)*	Xenobiotics	Food Component/Plant	1.00 (0.93, 1.08)	9.93E-01	9.94E-01	LC/MS Pos Early		3083620	100004059
Kynurenine	Amino Acid	Tryptophan Metabolism	1.00 (0.93, 1.08)	9.98E-01	9.98E-01	LC/MS Pos Early	HMDB00684	161166	100000265

†Models were adjusted for age, race, education, family history of breast cancer, age at menarche, oral contraceptive use, postmenopausal hormone use, parity and age at first birth.

Supplemental Table S2. Results of analyses to define metabolite blocks and lead metabolites associated with breast cancer based on the heirarchial heat map shown in Figure S1.

			Original Breast Cancer Association				Round 1 - Adjust for Lead			
Block	Number	Metabolite	RR (95% CI)†	P	FDR	Lead #	Lead R	RR (95% CI)†	P	Final Block?
A	1	21-Hydroxypregnenolone disulfate	1.13 (1.04, 1.22)	2.90E-03	1.14E-01		0.866	1.05 (0.90, 1.23)	5.51E-01	Yes
A	2	Androstenediol (3β,17β) disulfate (2)	1.13 (1.05, 1.22)	1.86E-03	1.03E-01		1.000	-	-	
A	3	Androstenediol (3α, 17α) monosulfate (2)	1.11 (1.03, 1.20)	8.15E-03	1.41E-01		0.683	1.04 (0.94, 1.15)	4.78E-01	
A	4	Pregnenolone sulfate	1.11 (1.03, 1.20)	9.72E-03	1.60E-01		0.659	1.04 (0.94, 1.16)	4.22E-01	
A	5	Andro steroid monosulfate C19H28O6S (1)*	1.13 (1.04, 1.22)	2.58E-03	1.13E-01	8	0.670	1.07 (0.97, 1.19)	1.86E-01	
A	6	16α-Hydroxy DHEA 3-sulfate	1.11 (1.03, 1.20)	7.15E-03	1.34E-01		0.685	1.05 (0.94, 1.17)	3.64E-01	
A	7	Androstenediol (3β,17β) monosulfate (1)	1.11 (1.02, 1.19)	1.08E-02	1.67E-01		0.745	1.03 (0.92, 1.15)	6.52E-01	
A	8	**Dehydroepiandrosterone sulfate (DHEA-S)**	1.11 (1.02, 1.20)	1.35E-02	1.94E-01		0.827	0.99 (0.86, 1.15)	9.22E-01	
A	9	Androstenediol (3β,17β) disulfate (1)	1.11 (1.03, 1.20)	5.99E-03	1.24E-01		0.731	1.04 (0.94, 1.16)	4.42E-01	
B	10	**(2,4 or 2,5)-Dimethylphenol sulfate**	1.12 (1.04, 1.20)	4.09E-03	1.15E-01	10	1.000	-	-	Separate
B	11	Syringol sulfate	1.12 (1.04, 1.21)	4.23E-03	1.15E-01		0.424	1.09 (1.00, 1.18)	4.51E-02	Separate
C	12	3-Hydroxypyridine glucuronide	1.12 (1.04, 1.20)	3.47E-03	1.14E-01		0.726	1.03 (0.93, 1.15)	5.81E-01	Yes
C	13	Catechol glucuronide	1.10 (1.02, 1.19)	8.85E-03	1.51E-01	15	0.720	1.01 (0.91, 1.12)	8.57E-01	
C	14	3-Ethylcatechol sulfate (2)	1.11 (1.04, 1.20)	3.34E-03	1.14E-01		0.871	0.98 (0.85, 1.13)	8.12E-01	
C	15	**3-Methyl catechol sulfate (2)**	1.14 (1.06, 1.23)	4.76E-04	3.81E-02		1.000	-	-	
D	16	LysoPE (18:2)*	0.88 (0.82, 0.94)	4.60E-04	3.81E-02		0.778	1.00 (0.88, 1.12)	9.37E-01	Yes
D	17	LysoPE (18:1)	0.87 (0.81, 0.94)	2.23E-04	3.42E-02		0.672	0.95 (0.87, 1.05)	3.25E-01	
D	18	LysoPE (18:0)	0.91 (0.84, 0.98)	1.05E-02	1.66E-01		0.532	0.99 (0.91, 1.08)	7.81E-01	
D	19	LysoPC (18:2)	0.87 (0.81, 0.94)	2.81E-04	3.49E-02		0.790	0.99 (0.88, 1.12)	8.97E-01	
D	20	LysoPC (18:1)	0.90 (0.83, 0.96)	3.01E-03	1.14E-01		0.656	1.00 (0.91, 1.10)	9.82E-01	
D	21	LysoPC (18:3)*	0.87 (0.81, 0.94)	4.83E-04	3.81E-02	25	0.624	0.95 (0.86, 1.05)	2.97E-01	
D	22	PI (18:0/18:1)*	0.90 (0.83, 0.97)	5.25E-03	1.24E-01		0.566	0.99 (0.90, 1.08)	7.57E-01	
D	23	PI (18:0/18:2)	0.90 (0.84, 0.97)	6.18E-03	1.24E-01		0.671	1.03 (0.93, 1.15)	5.64E-01	
D	24	PC (18:2/18:3)*	0.85 (0.79, 0.91)	1.58E-05	6.87E-03		0.811	0.91 (0.80, 1.04)	1.53E-01	
D	25	**PC (18:2/18:2)**	0.85 (0.80, 0.92)	1.19E-05	6.87E-03		1.000	-	-	
D	26	PE (18:2/18:2)*	0.90 (0.84, 0.97)	5.99E-03	1.24E-01		0.613	0.99 (0.90, 1.09)	8.95E-01	
D	27	PE (18:1/18:2)*	0.91 (0.85, 0.98)	1.34E-02	1.94E-01		0.608	1.01 (0.92, 1.11)	7.78E-01	
E	29	2-Hydroxysebacate	0.89 (0.83, 0.96)	1.89E-03	1.03E-01		0.595	0.96 (0.88, 1.05)	3.61E-01	
E	30	2-Hydroxydecanoate	0.87 (0.81, 0.94)	2.37E-04	3.42E-02		0.484	0.92 (0.85, 1.00)	4.88E-02	
E	31	12,13-DiHOME	0.89 (0.83, 0.96)	2.61E-03	1.13E-01	33	0.473	0.94 (0.87, 1.03)	1.89E-01	Yes
E	32	Undecanedioate (C11-DC)	0.91 (0.84, 0.97)	8.01E-03	1.41E-01		0.608	0.98 (0.90, 1.08)	7.06E-01	
E	33	**Azelate (C9-DC)**	0.87 (0.81, 0.93)	1.17E-04	3.38E-02		1.000	-	-	
E	34	Docosadioate (C22-DC)	0.90 (0.83, 0.97)	4.77E-03	1.24E-01		0.389	0.95 (0.87, 1.03)	1.83E-01	
F	35	Sphinganine-1-phosphate	0.91 (0.85, 0.97)	6.07E-03	1.24E-01		0.438	0.95 (0.88, 1.03)	2.03E-01	Yes
F	36	Sphinganine	0.91 (0.85, 0.98)	1.10E-02	1.67E-01		0.153	0.93 (0.87, 1.00)	5.36E-02	
F	37	LysoPE (O-18:0)*	0.91 (0.84, 0.98)	9.94E-03	1.60E-01		0.487	0.96 (0.89, 1.05)	3.85E-01	
F	38	Glycerophosphoethanolamine	0.91 (0.84, 0.97)	7.09E-03	1.34E-01		0.388	0.95 (0.88, 1.03)	2.18E-01	
F	39	PE (O-18:0/18:2)*	0.89 (0.83, 0.96)	1.41E-03	9.40E-02		0.733	0.96 (0.86, 1.07)	4.64E-01	
F	40	PE (O-16:0/18:2)*	0.90 (0.84, 0.97)	6.30E-03	1.24E-01		0.662	0.98 (0.89, 1.08)	7.21E-01	
F	41	PE (O-16:0/18:1)*	0.90 (0.84, 0.97)	5.10E-03	1.24E-01		0.611	0.97 (0.88, 1.06)	4.63E-01	
F	42	PE (O-18:0/18:1)	0.90 (0.83, 0.96)	2.41E-03	1.13E-01		0.625	0.96 (0.87, 1.05)	3.35E-01	
F	43	Arachidoylcarnitine (C20)*	0.89 (0.82, 0.96)	3.55E-03	1.14E-01		0.399	0.93 (0.86, 1.01)	1.04E-01	
F	44	**PC (O-16:0/18:2)***	0.87 (0.82, 0.94)	1.93E-04	3.42E-02		1.000	-	-	
F	45	PC (O-16:0/18:1)*	0.88 (0.82, 0.94)	3.32E-04	3.60E-02	44	0.852	0.95 (0.82, 1.10)	4.76E-01	
F	46	LysoPC (24:0)	0.90 (0.83, 0.97)	5.48E-03	1.24E-01		0.562	0.96 (0.87, 1.05)	3.32E-01	
F	47	Asparagine	0.91 (0.84, 0.97)	8.05E-03	1.41E-01		0.553	0.97 (0.89, 1.06)	5.04E-01	
F	48	Serine	0.89 (0.83, 0.96)	1.74E-03	1.03E-01		0.568	0.94 (0.87, 1.03)	2.06E-01	
F	49	Glutamine	0.91 (0.85, 0.98)	1.37E-02	1.94E-01		0.575	0.99 (0.90, 1.08)	7.49E-01	
F	50	PC (18:2/20:4n6)*	0.90 (0.84, 0.97)	5.02E-03	1.24E-01		0.579	0.97 (0.89, 1.06)	4.82E-01	
F	51	PC (18:0/18:2)*	0.90 (0.84, 0.97)	3.75E-03	1.15E-01		0.650	0.98 (0.89, 1.08)	6.70E-01	
F	52	SM (d18:1/24:0)	0.91 (0.84, 0.97)	7.25E-03	1.34E-01		0.582	0.97 (0.89, 1.06)	5.36E-01	
F	53	SM (d18:2/23:0, d18:1/23:1, d17:1/24:1)*	0.89 (0.83, 0.96)	2.31E-03	1.13E-01		0.608	0.96 (0.87, 1.05)	3.80E-01	
F	54	SM (d18:1/22:1, d18:2/22:0, d16:1/24:1)*	0.90 (0.84, 0.96)	3.03E-03	1.14E-01		0.638	0.97 (0.88, 1.07)	5.32E-01	
F	55	SM (d18:2/14:0, d18:1/14:1)*	0.90 (0.84, 0.97)	5.89E-03	1.24E-01		0.411	0.95 (0.88, 1.03)	2.33E-01	
G	56	N-Formylanthranilic acid	0.89 (0.83, 0.96)	4.09E-03	1.15E-01	57	0.448	0.93 (0.85, 1.02)	1.18E-01	Yes
G	57	**Phenylacetylglutamine**	0.88 (0.81, 0.95)	1.11E-03	8.04E-02		1.000	-	-	
H	59	Octadecadienedioate (C18:2-DC)*	0.89 (0.83, 0.96)	4.13E-03	1.15E-01	61	0.430	0.92 (0.85, 1.01)	6.53E-02	Yes
H	60	**S-Methylcysteine sulfoxide**	0.89 (0.83, 0.96)	3.56E-03	1.14E-01		1.000	-	-	

Round 2 - Adjust for new lead						
Number	Metabolite	Lead #	Lead R	RR (95% CI)†	P	Final Block?
29	2-Hydroxysebacate	30	0.74	0.97 (0.87, 1.08)	5.41E-01	Yes
30	**2-Hydroxydecanoate**		1.0			

†Models were adjusted for age, race, education, family history of breast cancer, age at menarche, oral contraceptive use, postmenopausal hormone use, parity and age at first birth.

Supplemental Table S3. The influence of adjustment for BMI on the associations of the 61 metabolites associated with breast cancer at FDR<0.20.

Metabolite	SUPER PATHWAY	SUBPATHWAY	Multivariable† Adjusted			Multivariable† + BMI Adjusted		
			RR (95% CI)†	P	FDR	RR (95% CI)†	P	FDR
PC (18:2/18:2)	Lipid	Phosphatidylcholine (PC)	0.85 (0.80, 0.92)	1.19E-05	6.87E-03	0.86 (0.80, 0.92)	3.97E-05	2.24E-02
PC (18:2/18:3)*	Lipid	Phosphatidylcholine (PC)	0.85 (0.79, 0.91)	1.58E-05	6.87E-03	0.85 (0.79, 0.92)	5.17E-05	2.24E-02
Azelate (C9-DC)	Lipid	Fatty Acid, Dicarboxylate	0.87 (0.81, 0.93)	1.17E-04	3.38E-02	0.87 (0.81, 0.93)	1.48E-04	4.28E-02
PC (O-16:0/18:2)*	Lipid	Plasmalogen	0.87 (0.82, 0.94)	1.93E-04	3.42E-02	0.88 (0.82, 0.95)	7.09E-04	8.79E-02
LysoPE (18:1)	Lipid	Lysophospholipid	0.87 (0.81, 0.94)	2.23E-04	3.42E-02	0.88 (0.82, 0.95)	5.30E-04	8.11E-02
2-Hydroxydecanoate	Lipid	Fatty Acid, Monohydroxy	0.87 (0.81, 0.94)	2.37E-04	3.42E-02	0.88 (0.81, 0.95)	5.60E-04	8.11E-02
LysoPC (18:2)	Lipid	Lysophospholipid	0.87 (0.81, 0.94)	2.81E-04	3.49E-02	0.88 (0.82, 0.95)	9.35E-04	9.51E-02
PC (O-16:0/18:1)*	Lipid	Plasmalogen	0.88 (0.82, 0.94)	3.32E-04	3.60E-02	0.88 (0.82, 0.95)	1.33E-03	9.87E-02
LysoPE (18:2)*	Lipid	Lysophospholipid	0.88 (0.82, 0.94)	4.60E-04	3.81E-02	0.88 (0.82, 0.95)	1.14E-03	9.87E-02
3-Methyl catechol sulfate (2)	Xenobiotics	Benzoate Metabolism	1.14 (1.06, 1.23)	4.76E-04	3.81E-02	1.15 (1.07, 1.24)	2.62E-04	5.69E-02
LysoPC (18:3)*	Lipid	Lysophospholipid	0.87 (0.81, 0.94)	4.83E-04	3.81E-02	0.88 (0.81, 0.95)	9.86E-04	9.51E-02
Phenylacetylglutamine	Peptide	Acetylated Peptides	0.88 (0.81, 0.95)	1.11E-03	8.04E-02	0.88 (0.82, 0.95)	1.36E-03	9.87E-02
PE (O-18:0/18:2)*	Lipid	Plasmalogen	0.89 (0.83, 0.96)	1.41E-03	9.40E-02	0.89 (0.83, 0.96)	2.93E-03	1.46E-01
Serine	Amino Acid	Glycine, Serine and Threonine Metabolism	0.89 (0.83, 0.96)	1.74E-03	1.03E-01	0.90 (0.83, 0.97)	4.74E-03	1.51E-01
Androstenediol (3β,17β) disulfate (2)	Lipid	Androgenic Steroids	1.13 (1.05, 1.22)	1.86E-03	1.03E-01	1.12 (1.04, 1.21)	3.37E-03	1.46E-01
2-Hydroxysebacate	Lipid	Fatty Acid, Dicarboxylate	0.89 (0.83, 0.96)	1.89E-03	1.03E-01	0.90 (0.83, 0.97)	3.67E-03	1.51E-01
SM (d18:2/23:0, d18:1/23:1, d17:1/24:1)*	Lipid	Sphingomyelins	0.89 (0.83, 0.96)	2.31E-03	1.13E-01	0.89 (0.83, 0.96)	3.31E-03	1.46E-01
PE (O-18:0/18:1)	Lipid	Plasmalogen	0.90 (0.83, 0.96)	2.41E-03	1.13E-01	0.90 (0.84, 0.97)	4.33E-03	1.51E-01
Andro steroid monosulfate C19H28O6S (1)*	Lipid	Androgenic Steroids	1.13 (1.04, 1.22)	2.58E-03	1.13E-01	1.12 (1.04, 1.21)	4.13E-03	1.51E-01
12,13-DiHOME	Lipid	Fatty Acid, Dihydroxy	0.89 (0.83, 0.96)	2.61E-03	1.13E-01	0.90 (0.83, 0.97)	4.86E-03	1.51E-01
21-Hydroxypregnenolone disulfate	Lipid	Pregnenolone Steroids	1.13 (1.04, 1.22)	2.90E-03	1.14E-01	1.13 (1.04, 1.22)	3.20E-03	1.46E-01
LysoPC (18:1)	Lipid	Lysophospholipid	0.90 (0.83, 0.96)	3.01E-03	1.14E-01	0.90 (0.84, 0.97)	8.17E-03	1.91E-01
SM (d18:1/22:1, d18:2/22:0, d16:1/24:1)*	Lipid	Sphingomyelins	0.90 (0.84, 0.96)	3.03E-03	1.14E-01	0.90 (0.84, 0.97)	4.04E-03	1.51E-01
3-Ethylcatechol sulfate (2)	Xenobiotics	Food Component/Plant	1.11 (1.04, 1.20)	3.34E-03	1.14E-01	1.12 (1.04, 1.21)	2.00E-03	1.24E-01
3-Hydroxypyridine glucuronide	Xenobiotics	Chemical	1.12 (1.04, 1.20)	3.47E-03	1.14E-01	1.12 (1.04, 1.21)	2.25E-03	1.30E-01
Arachidoylcarnitine (C20)*	Lipid	Fatty Acid Metabolism (Acyl Carnitine, Long Chain Saturated)	0.89 (0.82, 0.96)	3.55E-03	1.14E-01	0.90 (0.83, 0.97)	7.80E-03	1.91E-01
S-Methylcysteine sulfoxide	Amino Acid	Methionine, Cysteine, SAM and Taurine Metabolism	0.89 (0.83, 0.96)	3.56E-03	1.14E-01	0.90 (0.83, 0.97)	5.99E-03	1.79E-01
PC (18:0/18:2)*	Lipid	Phosphatidylcholine (PC)	0.90 (0.84, 0.97)	3.75E-03	1.15E-01	0.91 (0.85, 0.97)	7.09E-03	1.91E-01
(2,4 or 2,5)-Dimethylphenol sulfate	Xenobiotics	Food Component/Plant	1.12 (1.04, 1.20)	4.09E-03	1.15E-01	1.12 (1.04, 1.21)	2.85E-03	1.46E-01
N-Formylanthranilic acid	Amino Acid	Tryptophan Metabolism	0.89 (0.83, 0.96)	4.09E-03	1.15E-01	0.90 (0.83, 0.97)	7.44E-03	1.91E-01
Octadecadienedioate (C18:2-DC)*	Lipid	Fatty Acid, Dicarboxylate	0.89 (0.83, 0.96)	4.13E-03	1.15E-01	0.90 (0.83, 0.97)	8.48E-03	1.91E-01
Syringol sulfate	Xenobiotics	Food Component/Plant	1.12 (1.04, 1.21)	4.23E-03	1.15E-01	1.12 (1.04, 1.21)	4.63E-03	1.51E-01
Docosadioate (C22-DC)	Lipid	Fatty Acid, Dicarboxylate	0.90 (0.83, 0.97)	4.77E-03	1.24E-01	0.90 (0.84, 0.97)	7.32E-03	1.91E-01
PC (18:2/20:4n6)*	Lipid	Phosphatidylcholine (PC)	0.90 (0.84, 0.97)	5.02E-03	1.24E-01	0.91 (0.85, 0.98)	9.41E-03	1.91E-01
PE (O-16:0/18:1)*	Lipid	Plasmalogen	0.90 (0.84, 0.97)	5.10E-03	1.24E-01	0.91 (0.85, 0.98)	8.49E-03	1.91E-01
PI (18:0/18:1)*	Lipid	Phosphatidylinositol (PI)	0.90 (0.83, 0.97)	5.25E-03	1.24E-01	0.90 (0.84, 0.98)	9.67E-03	1.91E-01
LysoPC (24:0)	Lipid	Lysophospholipid	0.90 (0.83, 0.97)	5.48E-03	1.24E-01	0.91 (0.83, 0.98)	1.75E-02	2.49E-01
Hippurate	Xenobiotics	Benzoate Metabolism	0.90 (0.83, 0.97)	5.86E-03	1.24E-01	0.90 (0.83, 0.97)	9.15E-03	1.91E-01
SM (d18:2/14:0, d18:1/14:1)*	Lipid	Sphingomyelins	0.90 (0.84, 0.97)	5.89E-03	1.24E-01	0.89 (0.82, 0.96)	1.74E-03	1.17E-01
Androstenediol (3β,17β) disulfate (1)	Lipid	Androgenic Steroids	1.11 (1.03, 1.20)	5.99E-03	1.24E-01	1.10 (1.02, 1.19)	1.06E-02	2.01E-01
PE (18:2/18:2)*	Lipid	Phosphatidylethanolamine (PE)	0.90 (0.84, 0.97)	5.99E-03	1.24E-01	0.90 (0.84, 0.97)	8.69E-03	1.91E-01
Sphinganine-1-phosphate	Lipid	Sphingolipid Synthesis	0.91 (0.85, 0.97)	6.07E-03	1.24E-01	0.92 (0.85, 0.98)	1.30E-02	2.06E-01
PI (18:0/18:2)	Lipid	Phosphatidylinositol (PI)	0.90 (0.84, 0.97)	6.18E-03	1.24E-01	0.91 (0.84, 0.98)	1.01E-02	1.94E-01
PE (O-16:0/18:2)*	Lipid	Plasmalogen	0.90 (0.84, 0.97)	6.30E-03	1.24E-01	0.91 (0.84, 0.98)	9.68E-03	1.91E-01
Glycerophosphoethanolamine	Lipid	Phospholipid Metabolism	0.91 (0.84, 0.97)	7.09E-03	1.34E-01	0.91 (0.85, 0.98)	1.14E-02	2.06E-01
16α-Hydroxy DHEA 3-sulfate	Lipid	Androgenic Steroids	1.11 (1.03, 1.20)	7.15E-03	1.34E-01	1.11 (1.02, 1.20)	1.30E-02	2.06E-01
SM (d18:1/24:0)	Lipid	Sphingomyelins	0.91 (0.84, 0.97)	7.25E-03	1.34E-01	0.91 (0.85, 0.98)	1.21E-02	2.06E-01
Undecanedioate (C11-DC)	Lipid	Fatty Acid, Dicarboxylate	0.91 (0.84, 0.97)	8.01E-03	1.41E-01	0.91 (0.84, 0.98)	9.65E-03	1.91E-01
Asparagine	Amino Acid	Alanine and Aspartate Metabolism	0.91 (0.84, 0.97)	8.05E-03	1.41E-01	0.92 (0.85, 0.99)	2.28E-02	2.76E-01
Androstenediol (3α, 17α) monosulfate (2)	Lipid	Androgenic Steroids	1.11 (1.03, 1.20)	8.15E-03	1.41E-01	1.10 (1.02, 1.19)	1.16E-02	2.06E-01
Catechol glucuronide	Amino Acid	Tyrosine Metabolism	1.10 (1.02, 1.19)	8.85E-03	1.51E-01	1.11 (1.03, 1.20)	4.81E-03	1.51E-01
Pregnenolone sulfate	Lipid	Pregnenolone Steroids	1.11 (1.03, 1.20)	9.72E-03	1.60E-01	1.12 (1.03, 1.21)	6.85E-03	1.91E-01
4-Guanidinobutanoate	Amino Acid	Guanidino and Acetamido Metabolism	0.91 (0.85, 0.98)	9.82E-03	1.60E-01	0.91 (0.85, 0.98)	1.41E-02	2.14E-01
LysoPE (O-18:0)*	Lipid	Lysoplasmalogen	0.91 (0.84, 0.98)	9.94E-03	1.60E-01	0.91 (0.85, 0.99)	1.96E-02	2.55E-01
LysoPE (18:0)	Lipid	Lysophospholipid	0.91 (0.84, 0.98)	1.05E-02	1.66E-01	0.91 (0.85, 0.98)	1.74E-02	2.49E-01
Androstenediol (3β,17β) monosulfate (1)	Lipid	Androgenic Steroids	1.11 (1.02, 1.19)	1.08E-02	1.67E-01	1.10 (1.02, 1.19)	1.82E-02	2.49E-01
Sphinganine	Lipid	Sphingolipid Synthesis	0.91 (0.85, 0.98)	1.10E-02	1.67E-01	0.91 (0.85, 0.98)	8.55E-03	1.91E-01
β-Cryptoxanthin	Cofactors and Vitami	Vitamin A Metabolism	0.91 (0.84, 0.98)	1.33E-02	1.94E-01	0.92 (0.85, 1.00)	4.23E-02	3.37E-01
PE (18:1/18:2)*	Lipid	Phosphatidylethanolamine (PE)	0.91 (0.85, 0.98)	1.34E-02	1.94E-01	0.91 (0.85, 0.98)	1.69E-02	2.49E-01
Dehydroepiandrosterone sulfate (DHEA-S)	Lipid	Androgenic Steroids	1.11 (1.02, 1.20)	1.35E-02	1.94E-01	1.11 (1.02, 1.20)	1.25E-02	2.06E-01
Glutamine	Amino Acid	Glutamate Metabolism	0.91 (0.85, 0.98)	1.37E-02	1.94E-01	0.92 (0.86, 0.99)	2.73E-02	3.17E-01

†Models were adjusted for age, race, education, family history of breast cancer, age at menarche, oral contraceptive use, postmenopausal hormone use, parity and age at first birth.

Supplemental Table S4. Association of top 61 metabolites with premenopausal and postmenopausal breast cancer.

	**Premenopausal Cases N = 737**			**Postmenopausl Cases N = 882**			**Heterogeneity Estimate**
**Metabolite**	**RR (95% CI)†**	**P**	**FDR**	**RR (95% CI)†**	**P**	**FDR**	**P-interaction**
PC (18:2/18:2)	0.87 (0.78, 0.97)	1.33E-02	8.18E-01	0.84 (0.77, 0.93)	4.93E-04	5.35E-02	5.86E-01
PC (18:2/18:3)*	0.87 (0.77, 0.97)	1.14E-02	8.18E-01	0.85 (0.77, 0.95)	2.90E-03	1.01E-01	7.96E-01
Azelate (C9-DC)	0.89 (0.80, 0.99)	2.69E-02	8.18E-01	0.83 (0.75, 0.92)	2.93E-04	5.35E-02	2.09E-01
PC (O-16:0/18:2)*	0.88 (0.79, 0.99)	2.79E-02	8.18E-01	0.87 (0.79, 0.95)	3.68E-03	1.14E-01	7.89E-01
LysoPE (18:1)	0.88 (0.79, 0.99)	3.33E-02	8.18E-01	0.84 (0.76, 0.92)	3.07E-04	5.35E-02	2.90E-01
2-Hydroxydecanoate	0.86 (0.76, 0.96)	7.77E-03	8.18E-01	0.88 (0.79, 0.97)	1.12E-02	1.62E-01	6.56E-01
LysoPC (18:2)	0.85 (0.76, 0.96)	6.19E-03	8.18E-01	0.87 (0.79, 0.96)	5.40E-03	1.27E-01	7.25E-01
PC (O-16:0/18:1)*	0.92 (0.82, 1.03)	1.37E-01	9.24E-01	0.85 (0.77, 0.93)	5.76E-04	5.56E-02	1.23E-01
LysoPE (18:2)*	0.86 (0.76, 0.96)	7.66E-03	8.18E-01	0.87 (0.79, 0.96)	5.59E-03	1.28E-01	7.80E-01
3-Methyl catechol sulfate (2)	1.14 (1.02, 1.28)	2.34E-02	8.18E-01	1.17 (1.06, 1.30)	2.85E-03	1.01E-01	5.63E-01
LysoPC (18:3)*	0.86 (0.77, 0.97)	1.22E-02	8.18E-01	0.88 (0.79, 0.98)	1.93E-02	2.08E-01	6.49E-01
Phenylacetylglutamine	0.91 (0.80, 1.02)	1.14E-01	9.24E-01	0.85 (0.77, 0.94)	2.39E-03	1.01E-01	2.61E-01
PE (O-18:0/18:2)*	0.89 (0.79, 1.00)	5.61E-02	9.04E-01	0.89 (0.81, 0.98)	1.88E-02	2.06E-01	9.90E-01
Serine	0.93 (0.83, 1.04)	2.23E-01	9.24E-01	0.85 (0.77, 0.93)	8.85E-04	6.40E-02	5.42E-02
Androstenediol (3β,17β) disulfate (2)	1.02 (0.91, 1.15)	6.85E-01	9.56E-01	1.22 (1.09, 1.36)	4.44E-04	5.35E-02	**1.28E-03**
2-Hydroxysebacate	0.89 (0.80, 1.00)	4.06E-02	8.48E-01	0.87 (0.79, 0.97)	8.52E-03	1.41E-01	7.19E-01
SM (d18:2/23:0, d18:1/23:1, d17:1/24:1)*	0.89 (0.79, 1.00)	5.93E-02	9.20E-01	0.91 (0.82, 1.00)	4.86E-02	3.10E-01	7.06E-01
PE (O-18:0/18:1)	0.93 (0.82, 1.05)	2.37E-01	9.24E-01	0.87 (0.79, 0.95)	2.13E-03	1.00E-01	1.91E-01
Andro steroid monosulfate C19H28O6S (1)*	1.06 (0.95, 1.18)	3.31E-01	9.24E-01	1.18 (1.05, 1.32)	4.60E-03	1.23E-01	**4.64E-02**
12,13-DiHOME	0.88 (0.78, 0.99)	3.44E-02	8.18E-01	0.88 (0.79, 0.98)	1.49E-02	1.89E-01	9.82E-01
21-Hydroxypregnenolone disulfate	1.05 (0.94, 1.17)	3.76E-01	9.24E-01	1.21 (1.07, 1.37)	2.19E-03	1.00E-01	**1.04E-02**
LysoPC (18:1)	0.94 (0.84, 1.05)	2.66E-01	9.24E-01	0.86 (0.78, 0.95)	1.97E-03	1.00E-01	8.46E-02
SM (d18:1/22:1, d18:2/22:0, d16:1/24:1)*	0.90 (0.80, 1.01)	8.14E-02	9.24E-01	0.91 (0.82, 1.00)	4.22E-02	3.01E-01	8.85E-01
3-Ethylcatechol sulfate (2)	1.11 (0.99, 1.25)	7.39E-02	9.24E-01	1.15 (1.04, 1.27)	5.27E-03	1.27E-01	4.96E-01
3-Hydroxypyridine glucuronide	1.11 (0.99, 1.25)	7.24E-02	9.24E-01	1.12 (1.01, 1.24)	2.50E-02	2.35E-01	8.64E-01
Arachidoylcarnitine (C20)*	0.90 (0.79, 1.03)	1.14E-01	9.24E-01	0.88 (0.79, 0.97)	1.06E-02	1.62E-01	5.99E-01
S-Methylcysteine sulfoxide	0.92 (0.81, 1.04)	1.90E-01	9.24E-01	0.88 (0.80, 0.98)	1.61E-02	1.94E-01	4.69E-01
PC (18:0/18:2)*	0.87 (0.78, 0.98)	1.62E-02	8.18E-01	0.92 (0.84, 1.01)	8.89E-02	4.22E-01	2.50E-01
(2,4 or 2,5)-Dimethylphenol sulfate	1.15 (1.03, 1.29)	1.73E-02	8.18E-01	1.14 (1.03, 1.27)	1.38E-02	1.84E-01	8.83E-01
N-Formylanthranilic acid	0.95 (0.84, 1.07)	3.94E-01	9.38E-01	0.86 (0.78, 0.96)	4.78E-03	1.23E-01	7.67E-02
Octadecadienedioate (C18:2-DC)*	0.97 (0.86, 1.10)	6.60E-01	9.52E-01	0.85 (0.76, 0.95)	4.03E-03	1.21E-01	**1.67E-02**
Syringol sulfate	1.12 (0.99, 1.27)	7.67E-02	9.24E-01	1.15 (1.03, 1.28)	1.16E-02	1.65E-01	6.14E-01
Docosadioate (C22-DC)	0.91 (0.81, 1.01)	8.40E-02	9.24E-01	0.87 (0.79, 0.97)	1.11E-02	1.62E-01	4.87E-01
PC (18:2/20:4n6)*	0.92 (0.82, 1.03)	1.34E-01	9.24E-01	0.90 (0.82, 0.99)	2.76E-02	2.49E-01	7.78E-01
PE (O-16:0/18:1)*	0.94 (0.83, 1.07)	3.39E-01	9.24E-01	0.88 (0.80, 0.96)	4.81E-03	1.23E-01	1.67E-01
PI (18:0/18:1)*	0.89 (0.80, 1.00)	4.59E-02	8.48E-01	0.89 (0.80, 0.99)	2.70E-02	2.49E-01	9.64E-01
LysoPC (24:0)	0.95 (0.84, 1.07)	3.60E-01	9.24E-01	0.87 (0.78, 0.96)	8.28E-03	1.41E-01	1.15E-01
Hippurate	0.90 (0.80, 1.02)	8.95E-02	9.24E-01	0.90 (0.81, 1.00)	4.58E-02	3.06E-01	9.08E-01
SM (d18:2/14:0, d18:1/14:1)*	0.88 (0.78, 0.99)	3.33E-02	8.18E-01	0.94 (0.85, 1.03)	1.91E-01	5.72E-01	2.62E-01
Androstenediol (3β,17β) disulfate (1)	1.02 (0.90, 1.15)	7.82E-01	9.64E-01	1.20 (1.08, 1.33)	4.10E-04	5.35E-02	1.60E-03
PE (18:2/18:2)*	0.88 (0.79, 0.99)	2.86E-02	8.18E-01	0.90 (0.81, 0.99)	3.86E-02	2.97E-01	7.56E-01
Sphinganine-1-phosphate	0.94 (0.84, 1.05)	2.83E-01	9.24E-01	0.87 (0.80, 0.96)	4.58E-03	1.23E-01	1.50E-01
PI (18:0/18:2)	0.88 (0.78, 0.99)	2.79E-02	8.18E-01	0.91 (0.83, 1.01)	8.45E-02	4.10E-01	4.58E-01
PE (O-16:0/18:2)*	0.93 (0.82, 1.04)	1.90E-01	9.24E-01	0.91 (0.82, 1.00)	4.85E-02	3.10E-01	6.91E-01
Glycerophosphoethanolamine	0.93 (0.83, 1.04)	1.86E-01	9.24E-01	0.87 (0.79, 0.96)	6.01E-03	1.30E-01	2.54E-01
16α-Hydroxy DHEA 3-sulfate	1.06 (0.95, 1.19)	2.88E-01	9.24E-01	1.14 (1.02, 1.28)	2.38E-02	2.32E-01	1.76E-01
SM (d18:1/24:0)	0.92 (0.82, 1.05)	2.08E-01	9.24E-01	0.90 (0.82, 0.99)	3.36E-02	2.70E-01	6.66E-01
Undecanedioate (C11-DC)	0.86 (0.78, 0.96)	5.26E-03	8.18E-01	0.91 (0.82, 1.02)	1.14E-01	4.65E-01	2.88E-01
Asparagine	0.92 (0.82, 1.03)	1.44E-01	9.24E-01	0.88 (0.79, 0.97)	1.34E-02	1.84E-01	3.82E-01
Androstenediol (3α, 17α) monosulfate (2)	1.00 (0.89, 1.11)	9.46E-01	9.89E-01	1.20 (1.07, 1.35)	1.81E-03	1.00E-01	**5.51E-04**
Catechol glucuronide	1.09 (0.98, 1.22)	1.22E-01	9.24E-01	1.12 (1.02, 1.24)	2.44E-02	2.35E-01	5.73E-01
Pregnenolone sulfate	1.04 (0.93, 1.16)	5.36E-01	9.52E-01	1.17 (1.03, 1.32)	1.37E-02	1.84E-01	3.06E-02
4-Guanidinobutanoate	0.94 (0.84, 1.05)	2.54E-01	9.24E-01	0.90 (0.81, 0.99)	3.25E-02	2.70E-01	4.26E-01
LysoPE (O-18:0)*	0.90 (0.80, 1.01)	6.73E-02	9.24E-01	0.90 (0.81, 0.99)	3.74E-02	2.91E-01	9.72E-01
LysoPE (18:0)	0.92 (0.82, 1.03)	1.26E-01	9.24E-01	0.88 (0.80, 0.98)	1.41E-02	1.86E-01	4.85E-01
Androstenediol (3β,17β) monosulfate (1)	0.99 (0.88, 1.11)	8.56E-01	9.72E-01	1.22 (1.09, 1.37)	4.19E-04	5.35E-02	**7.09E-05**
Sphinganine	0.98 (0.88, 1.10)	7.66E-01	9.64E-01	0.87 (0.79, 0.96)	3.58E-03	1.14E-01	**1.80E-02**
β-Cryptoxanthin	0.94 (0.83, 1.06)	3.24E-01	9.24E-01	0.90 (0.81, 1.00)	4.68E-02	3.08E-01	4.19E-01
PE (18:1/18:2)*	0.92 (0.82, 1.02)	1.27E-01	9.24E-01	0.88 (0.79, 0.98)	1.73E-02	1.98E-01	4.04E-01
Dehydroepiandrosterone sulfate (DHEA-S)	1.00 (0.89, 1.12)	9.88E-01	9.97E-01	1.21 (1.07, 1.36)	1.94E-03	1.00E-01	**9.79E-04**
Glutamine	0.90 (0.80, 1.01)	6.48E-02	9.24E-01	0.92 (0.83, 1.01)	8.42E-02	4.10E-01	7.23E-01

†Models were adjusted for age, race, education, family history of breast cancer, age at menarche, oral contraceptive use, postmenopausal

Supplemental Table S5. Association of top 61 metabolites with ER+ and ER- breast cancer.

	ER+		Cases	ER-		Cases	Heterogeneity
		N=1,410			N=252		Estimate
**Metabolite**	**RR (95% CI)†**	**P**	**FDR**	**RR (95% CI)†**	**P**	**FDR**	**P-value**
PC (18:2/18:2)	0.85 (0.79, 0.92)	3.13E-05	2.08E-02	0.83 (0.73, 0.95)	6.43E-03	5.58E-01	7.59E-01
PC (18:2/18:3)*	0.85 (0.79, 0.92)	5.79E-05	2.08E-02	0.82 (0.71, 0.94)	4.65E-03	5.58E-01	6.59E-01
Azelate (C9-DC)	0.86 (0.80, 0.93)	9.56E-05	2.08E-02	0.89 (0.77, 1.02)	9.71E-02	8.36E-01	6.73E-01
PC (O-16:0/18:2)*	0.87 (0.81, 0.94)	2.12E-04	2.30E-02	0.88 (0.77, 1.01)	7.53E-02	8.17E-01	8.85E-01
LysoPE (18:1)	0.87 (0.80, 0.94)	2.05E-04	2.30E-02	0.89 (0.77, 1.03)	1.12E-01	8.68E-01	7.89E-01
2-Hydroxydecanoate	0.86 (0.79, 0.93)	7.44E-05	2.08E-02	0.93 (0.80, 1.09)	4.02E-01	9.97E-01	3.80E-01
LysoPC (18:2)	0.87 (0.80, 0.93)	1.84E-04	2.30E-02	0.90 (0.79, 1.04)	1.59E-01	9.89E-01	6.72E-01
PC (O-16:0/18:1)*	0.87 (0.81, 0.94)	3.17E-04	3.06E-02	0.89 (0.77, 1.02)	9.04E-02	8.36E-01	7.79E-01
LysoPE (18:2)*	0.87 (0.81, 0.94)	5.93E-04	3.96E-02	0.88 (0.77, 1.02)	8.11E-02	8.20E-01	8.88E-01
3-Methyl catechol sulfate (2)	1.16 (1.07, 1.25)	1.72E-04	2.30E-02	1.04 (0.91, 1.18)	5.78E-01	9.97E-01	1.57E-01
LysoPC (18:3)*	0.88 (0.81, 0.95)	1.83E-03	7.22E-02	0.82 (0.70, 0.95)	7.16E-03	5.61E-01	4.22E-01
Phenylacetylglutamine	0.88 (0.81, 0.96)	2.25E-03	7.50E-02	0.88 (0.76, 1.02)	8.22E-02	8.20E-01	1.00E+00
PE (O-18:0/18:2)*	0.89 (0.82, 0.96)	2.78E-03	8.33E-02	0.86 (0.75, 0.99)	3.60E-02	6.51E-01	6.74E-01
Serine	0.88 (0.82, 0.95)	1.39E-03	6.34E-02	0.90 (0.79, 1.03)	1.31E-01	9.07E-01	7.72E-01
Androstenediol (3β,17β) disulfate (2)	1.16 (1.07, 1.26)	3.81E-04	3.30E-02	0.96 (0.83, 1.11)	5.89E-01	9.97E-01	**2.61E-02**
2-Hydroxysebacate	0.88 (0.81, 0.95)	6.84E-04	3.96E-02	0.95 (0.83, 1.10)	5.26E-01	9.97E-01	3.54E-01
SM (d18:2/23:0, d18:1/23:1, d17:1/24:1)*	0.88 (0.82, 0.95)	1.46E-03	6.34E-02	0.92 (0.79, 1.07)	2.99E-01	9.97E-01	6.05E-01
PE (O-18:0/18:1)	0.90 (0.84, 0.97)	6.65E-03	1.36E-01	0.86 (0.74, 0.99)	3.12E-02	6.24E-01	5.83E-01
Andro steroid monosulfate C19H28O6S (1)*	1.14 (1.05, 1.23)	1.71E-03	7.09E-02	1.05 (0.89, 1.22)	5.75E-01	9.97E-01	3.61E-01
12,13-DiHOME	0.88 (0.81, 0.95)	2.05E-03	7.42E-02	0.92 (0.80, 1.06)	2.55E-01	9.97E-01	5.90E-01
21-Hydroxypregnenolone disulfate	1.15 (1.06, 1.25)	1.21E-03	5.84E-02	0.99 (0.85, 1.15)	8.73E-01	9.97E-01	8.81E-02
LysoPC (18:1)	0.89 (0.82, 0.96)	2.57E-03	8.00E-02	0.91 (0.79, 1.05)	1.88E-01	9.97E-01	7.89E-01
SM (d18:1/22:1, d18:2/22:0, d16:1/24:1)*	0.88 (0.82, 0.95)	1.06E-03	5.76E-02	0.96 (0.83, 1.12)	5.96E-01	9.97E-01	3.07E-01
3-Ethylcatechol sulfate (2)	1.14 (1.06, 1.23)	6.55E-04	3.96E-02	1.00 (0.88, 1.15)	9.66E-01	9.97E-01	9.34E-02
3-Hydroxypyridine glucuronide	1.14 (1.06, 1.23)	6.70E-04	3.96E-02	0.98 (0.85, 1.14)	8.26E-01	9.97E-01	7.16E-02
Arachidoylcarnitine (C20)*	0.89 (0.82, 0.96)	3.98E-03	1.05E-01	0.88 (0.75, 1.03)	1.08E-01	8.54E-01	9.00E-01
S-Methylcysteine sulfoxide	0.90 (0.83, 0.97)	9.11E-03	1.44E-01	0.80 (0.68, 0.95)	8.69E-03	5.61E-01	2.11E-01
PC (18:0/18:2)*	0.90 (0.84, 0.97)	4.53E-03	1.10E-01	0.89 (0.79, 1.01)	6.30E-02	7.62E-01	8.78E-01
(2,4 or 2,5)-Dimethylphenol sulfate	1.13 (1.05, 1.22)	2.23E-03	7.50E-02	1.05 (0.92, 1.19)	4.70E-01	9.97E-01	3.34E-01
N-Formylanthranilic acid	0.90 (0.83, 0.97)	8.28E-03	1.36E-01	0.86 (0.75, 0.98)	2.87E-02	6.24E-01	5.65E-01
Octadecadienedioate (C18:2-DC)*	0.90 (0.83, 0.98)	1.12E-02	1.50E-01	0.87 (0.74, 1.01)	6.36E-02	7.62E-01	7.06E-01
Syringol sulfate	1.13 (1.04, 1.22)	4.87E-03	1.10E-01	1.08 (0.97, 1.22)	1.60E-01	9.89E-01	5.25E-01
Docosadioate (C22-DC)	0.89 (0.82, 0.96)	3.59E-03	9.74E-02	0.94 (0.82, 1.09)	4.15E-01	9.97E-01	5.10E-01
PC (18:2/20:4n6)*	0.91 (0.84, 0.97)	7.93E-03	1.36E-01	0.89 (0.78, 1.01)	6.41E-02	7.62E-01	7.68E-01
PE (O-16:0/18:1)*	0.91 (0.84, 0.98)	9.62E-03	1.46E-01	0.88 (0.77, 1.01)	7.52E-02	8.17E-01	6.74E-01
PI (18:0/18:1)*	0.92 (0.85, 0.99)	2.72E-02	2.22E-01	0.81 (0.70, 0.93)	3.23E-03	4.68E-01	1.22E-01
LysoPC (24:0)	0.89 (0.82, 0.97)	5.55E-03	1.18E-01	0.89 (0.77, 1.03)	1.16E-01	8.73E-01	1.00E+00
Hippurate	0.89 (0.82, 0.96)	4.15E-03	1.06E-01	0.91 (0.78, 1.07)	2.43E-01	9.97E-01	8.05E-01
SM (d18:2/14:0, d18:1/14:1)*	0.91 (0.84, 0.98)	1.25E-02	1.57E-01	0.88 (0.76, 1.01)	6.18E-02	7.62E-01	6.85E-01
Androstenediol (3β,17β) disulfate (1)	1.15 (1.06, 1.25)	5.00E-04	3.95E-02	0.87 (0.76, 1.01)	6.93E-02	8.13E-01	8.77E-04
PE (18:2/18:2)*	0.89 (0.83, 0.97)	4.83E-03	1.10E-01	0.93 (0.81, 1.07)	2.97E-01	9.97E-01	5.89E-01
Sphinganine-1-phosphate	0.90 (0.84, 0.97)	5.07E-03	1.10E-01	0.92 (0.81, 1.05)	2.12E-01	9.97E-01	7.72E-01
PI (18:0/18:2)	0.91 (0.84, 0.98)	1.58E-02	1.76E-01	0.85 (0.74, 0.98)	2.10E-02	5.90E-01	4.04E-01
PE (O-16:0/18:2)*	0.90 (0.83, 0.97)	7.89E-03	1.36E-01	0.90 (0.78, 1.03)	1.22E-01	8.82E-01	1.00E+00
Glycerophosphoethanolamine	0.91 (0.84, 0.98)	1.30E-02	1.59E-01	0.86 (0.76, 0.99)	3.11E-02	6.24E-01	4.69E-01
16α-Hydroxy DHEA 3-sulfate	1.12 (1.04, 1.22)	5.04E-03	1.10E-01	1.03 (0.88, 1.21)	6.71E-01	9.97E-01	3.57E-01
SM (d18:1/24:0)	0.90 (0.84, 0.97)	6.80E-03	1.36E-01	0.90 (0.79, 1.04)	1.57E-01	9.89E-01	1.00E+00
Undecanedioate (C11-DC)	0.90 (0.83, 0.97)	6.96E-03	1.36E-01	0.93 (0.80, 1.08)	3.55E-01	9.97E-01	7.04E-01
Asparagine	0.90 (0.83, 0.97)	4.87E-03	1.10E-01	0.93 (0.80, 1.07)	3.02E-01	9.97E-01	6.97E-01
Androstenediol (3α, 17α) monosulfate (2)	1.12 (1.03, 1.21)	7.79E-03	1.36E-01	1.05 (0.90, 1.22)	5.41E-01	9.97E-01	4.62E-01
Catechol glucuronide	1.13 (1.05, 1.22)	1.20E-03	5.84E-02	0.95 (0.82, 1.09)	4.55E-01	9.97E-01	**3.45E-02**
Pregnenolone sulfate	1.12 (1.03, 1.22)	7.61E-03	1.36E-01	1.01 (0.87, 1.18)	8.89E-01	9.97E-01	2.45E-01
4-Guanidinobutanoate	0.91 (0.85, 0.98)	1.67E-02	1.76E-01	0.92 (0.80, 1.05)	2.08E-01	9.97E-01	8.89E-01
LysoPE (O-18:0)*	0.91 (0.84, 0.99)	2.25E-02	2.04E-01	0.86 (0.75, 0.98)	2.36E-02	6.02E-01	4.80E-01
LysoPE (18:0)	0.92 (0.85, 0.99)	2.86E-02	2.25E-01	0.86 (0.75, 0.98)	2.82E-02	6.24E-01	3.91E-01
Androstenediol (3β,17β) monosulfate (1)	1.14 (1.05, 1.23)	1.99E-03	7.42E-02	0.93 (0.80, 1.07)	3.24E-01	9.97E-01	1.59E-02
Sphinganine	0.92 (0.86, 0.99)	3.31E-02	2.42E-01	0.82 (0.71, 0.93)	2.97E-03	4.68E-01	1.38E-01
β-Cryptoxanthin	0.89 (0.82, 0.96)	3.59E-03	9.74E-02	0.98 (0.85, 1.12)	7.48E-01	9.97E-01	2.35E-01
PE (18:1/18:2)*	0.90 (0.84, 0.98)	1.09E-02	1.49E-01	0.94 (0.81, 1.08)	3.57E-01	9.97E-01	6.01E-01
Dehydroepiandrosterone sulfate (DHEA-S)	1.12 (1.03, 1.22)	7.57E-03	1.36E-01	1.00 (0.86, 1.17)	9.96E-01	1.00E+00	2.06E-01
Glutamine	0.91 (0.84, 0.98)	1.03E-02	1.49E-01	0.93 (0.81, 1.07)	2.92E-01	9.97E-01	7.89E-01

†Models were adjusted for age, race, education, family history of breast cancer, age at menarche, oral contraceptive use, postmenopausal

Supplemental Table S6. Associations of top metabolites with breast cancer stratified by time between blood draw and diagnosis.

Metabolite	0 - <= 1.5 years Cases N =606			>1.5 years - 3 years Cases N = 534			> 3 years Cases N = 549			Heterogeneity Estimate
	RR (95% CI)†	P	FDR	RR (95% CI)†	P	FDR	RR (95% CI)†	P	FDR	P-value
PC (18:2/18:2)	0.86 (0.78, 0.94)	1.51E-03	7.80E-02	0.87 (0.80, 0.95)	2.97E-03	6.44E-01	0.90 (0.80, 1.02)	9.42E-02	9.10E-01	3.60E-01
PC (18:2/18:3)*	0.92 (0.84, 1.01)	9.15E-02	3.68E-01	0.85 (0.77, 0.94)	1.61E-03	6.44E-01	0.85 (0.75, 0.97)	1.61E-02	7.18E-01	7.08E-01
Azelate (C9-DC)	0.95 (0.87, 1.04)	2.81E-01	5.71E-01	0.92 (0.83, 1.02)	1.12E-01	8.15E-01	0.82 (0.73, 0.92)	1.09E-03	6.21E-01	1.74E-01
PC (O-16:0/18:2)*	0.91 (0.83, 1.00)	5.47E-02	3.03E-01	0.88 (0.81, 0.97)	8.75E-03	8.15E-01	0.88 (0.78, 0.99)	3.77E-02	8.84E-01	7.93E-01
LysoPE (18:1)	0.87 (0.79, 0.95)	3.27E-03	1.01E-01	0.92 (0.84, 1.01)	9.82E-02	8.15E-01	0.89 (0.79, 1.00)	5.68E-02	9.10E-01	8.20E-01
2-Hydroxydecanoate	0.89 (0.81, 0.99)	2.35E-02	2.34E-01	0.95 (0.86, 1.05)	3.12E-01	9.08E-01	0.85 (0.75, 0.96)	1.15E-02	7.18E-01	2.95E-01
LysoPC (18:2)	0.88 (0.80, 0.97)	8.39E-03	1.63E-01	0.90 (0.82, 0.99)	3.01E-02	8.15E-01	0.90 (0.79, 1.02)	8.82E-02	9.10E-01	6.80E-01
PC (O-16:0/18:1)*	0.92 (0.84, 1.01)	7.65E-02	3.44E-01	0.88 (0.80, 0.96)	4.43E-03	7.68E-01	0.88 (0.78, 0.99)	3.44E-02	8.77E-01	9.81E-01
LysoPE (18:2)*	0.90 (0.82, 0.99)	3.44E-02	2.73E-01	0.91 (0.83, 1.00)	5.46E-02	8.15E-01	0.87 (0.77, 0.99)	2.94E-02	8.51E-01	6.79E-01
3-Methyl catechol sulfate (2)	1.08 (0.99, 1.18)	7.60E-02	3.44E-01	1.05 (0.95, 1.16)	3.25E-01	9.08E-01	1.21 (1.07, 1.37)	2.15E-03	6.21E-01	6.83E-01
LysoPC (18:3)*	0.88 (0.79, 0.97)	1.17E-02	1.97E-01	0.91 (0.83, 1.01)	6.64E-02	8.15E-01	0.88 (0.77, 1.00)	4.98E-02	9.10E-01	4.14E-01
Phenylacetylglutamine	0.88 (0.80, 0.97)	1.33E-02	1.97E-01	0.90 (0.82, 0.99)	3.86E-02	8.15E-01	0.89 (0.78, 1.02)	9.22E-02	9.10E-01	8.02E-01
PE (O-18:0/18:2)*	0.91 (0.83, 1.00)	4.12E-02	2.79E-01	0.91 (0.83, 1.00)	4.46E-02	8.15E-01	0.91 (0.80, 1.02)	1.07E-01	9.10E-01	8.41E-01
Serine	0.91 (0.83, 1.00)	3.87E-02	2.76E-01	0.91 (0.83, 1.00)	4.91E-02	8.15E-01	0.91 (0.81, 1.03)	1.41E-01	9.11E-01	9.24E-01
Androstenediol (3β,17β) disulfate (2)	1.04 (0.94, 1.15)	4.09E-01	6.67E-01	1.09 (0.98, 1.20)	9.99E-02	8.15E-01	1.18 (1.04, 1.35)	1.09E-02	7.18E-01	1.69E-01
2-Hydroxysebacate	0.96 (0.87, 1.05)	3.58E-01	6.34E-01	0.94 (0.85, 1.03)	1.97E-01	8.57E-01	0.86 (0.76, 0.97)	1.35E-02	7.18E-01	7.64E-02
SM (d18:2/23:0, d18:1/23:1, d17:1/24:1)*	0.88 (0.81, 0.97)	7.18E-03	1.58E-01	0.96 (0.87, 1.05)	3.56E-01	9.08E-01	0.88 (0.77, 1.00)	4.31E-02	9.10E-01	9.08E-01
PE (O-18:0/18:1)	0.90 (0.82, 0.99)	3.37E-02	2.73E-01	0.94 (0.86, 1.03)	2.05E-01	8.62E-01	0.89 (0.79, 1.00)	4.53E-02	9.10E-01	9.93E-01
Andro steroid monosulfate C19H28O6S (1)*	1.07 (0.97, 1.17)	1.62E-01	4.71E-01	1.08 (0.98, 1.20)	1.21E-01	8.15E-01	1.17 (1.03, 1.34)	1.76E-02	7.26E-01	3.85E-01
12,13-DiHOME	0.87 (0.79, 0.96)	5.44E-03	1.35E-01	0.91 (0.82, 1.01)	8.17E-02	8.15E-01	0.93 (0.82, 1.06)	2.59E-01	9.18E-01	3.06E-01
21-Hydroxypregnenolone disulfate	1.07 (0.96, 1.19)	2.08E-01	5.18E-01	1.03 (0.93, 1.14)	5.86E-01	9.57E-01	1.24 (1.08, 1.41)	1.45E-03	6.21E-01	1.56E-01
LysoPC (18:1)	0.86 (0.78, 0.94)	8.87E-04	7.80E-02	0.94 (0.86, 1.03)	1.89E-01	8.48E-01	0.94 (0.82, 1.06)	3.08E-01	9.18E-01	1.74E-01
SM (d18:1/22:1, d18:2/22:0, d16:1/24:1)*	0.87 (0.80, 0.95)	1.38E-03	7.80E-02	0.94 (0.86, 1.03)	2.13E-01	8.62E-01	0.91 (0.81, 1.04)	1.63E-01	9.11E-01	4.75E-01
3-Ethylcatechol sulfate (2)	1.08 (0.99, 1.17)	8.17E-02	3.51E-01	1.06 (0.97, 1.16)	2.24E-01	8.72E-01	1.15 (1.02, 1.31)	2.39E-02	7.84E-01	8.42E-01
3-Hydroxypyridine glucuronide	1.07 (0.98, 1.17)	1.19E-01	4.06E-01	1.08 (0.99, 1.18)	9.64E-02	8.15E-01	1.15 (1.01, 1.31)	2.85E-02	8.51E-01	7.94E-01
Arachidoylcarnitine (C20)*	0.91 (0.83, 1.01)	6.41E-02	3.18E-01	0.96 (0.87, 1.06)	4.47E-01	9.48E-01	0.92 (0.81, 1.04)	1.74E-01	9.11E-01	9.67E-01
S-Methylcysteine sulfoxide	0.93 (0.84, 1.02)	1.21E-01	4.09E-01	0.89 (0.80, 0.99)	3.40E-02	8.15E-01	0.91 (0.79, 1.03)	1.44E-01	9.11E-01	6.33E-01
PC (18:0/18:2)*	0.87 (0.81, 0.95)	9.62E-04	7.80E-02	0.95 (0.87, 1.03)	2.28E-01	8.72E-01	0.94 (0.83, 1.06)	3.29E-01	9.18E-01	2.87E-01
(2,4 or 2,5)-Dimethylphenol sulfate	1.03 (0.94, 1.13)	5.59E-01	7.57E-01	1.03 (0.94, 1.14)	5.19E-01	9.57E-01	1.15 (1.02, 1.30)	2.16E-02	7.84E-01	4.67E-01
N-Formylanthranilic acid	0.94 (0.85, 1.04)	2.34E-01	5.44E-01	0.87 (0.79, 0.97)	8.95E-03	8.15E-01	0.92 (0.82, 1.04)	1.76E-01	9.11E-01	8.74E-01
Octadecadienedioate (C18:2-DC)*	1.01 (0.92, 1.11)	7.70E-01	8.90E-01	0.93 (0.83, 1.03)	1.75E-01	8.38E-01	0.83 (0.73, 0.94)	4.49E-03	7.18E-01	**3.99E-02**
Syringol sulfate	1.05 (0.96, 1.15)	2.78E-01	5.70E-01	1.00 (0.90, 1.10)	9.46E-01	9.94E-01	1.15 (1.02, 1.31)	2.28E-02	7.84E-01	3.80E-01
Docosadioate (C22-DC)	0.97 (0.88, 1.07)	4.97E-01	7.24E-01	0.90 (0.82, 0.99)	3.35E-02	8.15E-01	0.88 (0.76, 1.00)	5.45E-02	9.10E-01	3.29E-01
PC (18:2/20:4n6)*	0.87 (0.80, 0.95)	1.63E-03	7.80E-02	0.98 (0.90, 1.08)	7.19E-01	9.72E-01	0.89 (0.78, 1.01)	6.12E-02	9.10E-01	5.35E-01
PE (O-16:0/18:1)*	0.90 (0.82, 0.99)	2.84E-02	2.48E-01	0.96 (0.87, 1.05)	3.38E-01	9.08E-01	0.90 (0.80, 1.01)	7.39E-02	9.10E-01	7.83E-01
PI (18:0/18:1)*	0.90 (0.81, 0.99)	2.48E-02	2.40E-01	0.94 (0.85, 1.04)	2.05E-01	8.62E-01	0.91 (0.81, 1.03)	1.51E-01	9.11E-01	8.22E-01
LysoPC (24:0)	0.87 (0.79, 0.95)	2.87E-03	9.97E-02	0.91 (0.82, 1.01)	7.13E-02	8.15E-01	0.95 (0.84, 1.08)	4.15E-01	9.18E-01	1.06E-01
Hippurate	0.95 (0.86, 1.04)	2.80E-01	5.70E-01	0.87 (0.79, 0.97)	1.02E-02	8.15E-01	0.92 (0.80, 1.05)	2.05E-01	9.18E-01	7.65E-01
SM (d18:2/14:0, d18:1/14:1)*	0.86 (0.79, 0.95)	1.77E-03	7.80E-02	0.99 (0.90, 1.09)	8.40E-01	9.86E-01	0.88 (0.77, 1.00)	4.94E-02	9.10E-01	5.37E-01
Androstenediol (3β,17β) disulfate (1)	1.04 (0.94, 1.15)	4.12E-01	6.69E-01	1.07 (0.97, 1.17)	1.71E-01	8.38E-01	1.11 (0.98, 1.24)	9.61E-02	9.10E-01	7.80E-01
PE (18:2/18:2)*	0.91 (0.82, 1.00)	6.04E-02	3.14E-01	0.92 (0.83, 1.01)	9.01E-02	8.15E-01	0.92 (0.81, 1.04)	1.64E-01	9.11E-01	9.07E-01
Sphinganine-1-phosphate	0.87 (0.79, 0.95)	1.89E-03	7.80E-02	0.93 (0.85, 1.02)	1.25E-01	8.15E-01	0.95 (0.85, 1.06)	3.56E-01	9.18E-01	**3.85E-02**
PI (18:0/18:2)	0.92 (0.84, 1.02)	1.05E-01	3.88E-01	0.92 (0.83, 1.02)	9.56E-02	8.15E-01	0.92 (0.81, 1.04)	1.78E-01	9.11E-01	8.60E-01
PE (O-16:0/18:2)*	0.92 (0.84, 1.01)	8.88E-02	3.64E-01	0.93 (0.85, 1.02)	1.14E-01	8.15E-01	0.92 (0.81, 1.04)	1.90E-01	9.13E-01	9.60E-01
Glycerophosphoethanolamine	0.92 (0.84, 1.01)	7.34E-02	3.41E-01	0.95 (0.87, 1.03)	2.24E-01	8.72E-01	0.90 (0.80, 1.02)	9.89E-02	9.10E-01	8.08E-01
16α-Hydroxy DHEA 3-sulfate	1.06 (0.96, 1.17)	2.52E-01	5.51E-01	1.11 (1.00, 1.22)	5.41E-02	8.15E-01	1.13 (0.99, 1.29)	6.74E-02	9.10E-01	5.43E-01
SM (d18:1/24:0)	0.86 (0.79, 0.93)	4.79E-04	7.80E-02	0.93 (0.85, 1.03)	1.64E-01	8.36E-01	0.97 (0.86, 1.09)	6.17E-01	9.64E-01	7.91E-02
Undecanedioate (C11-DC)	0.94 (0.85, 1.04)	2.06E-01	5.18E-01	0.92 (0.83, 1.01)	8.20E-02	8.15E-01	0.90 (0.80, 1.01)	6.92E-02	9.10E-01	7.97E-01
Asparagine	0.93 (0.84, 1.02)	1.10E-01	3.91E-01	0.94 (0.85, 1.03)	1.97E-01	8.57E-01	0.91 (0.80, 1.03)	1.19E-01	9.11E-01	8.29E-01
Androstenediol (3α, 17α) monosulfate (2)	1.00 (0.91, 1.10)	9.81E-01	9.90E-01	1.08 (0.97, 1.19)	1.69E-01	8.38E-01	1.21 (1.07, 1.37)	3.12E-03	6.77E-01	1.54E-02
Catechol glucuronide	1.06 (0.97, 1.15)	2.24E-01	5.32E-01	1.12 (1.03, 1.23)	1.20E-02	8.15E-01	1.12 (0.98, 1.27)	9.27E-02	9.10E-01	9.21E-01
Pregnenolone sulfate	1.07 (0.97, 1.19)	1.83E-01	5.01E-01	1.02 (0.91, 1.13)	7.76E-01	9.79E-01	1.19 (1.05, 1.36)	8.05E-03	7.18E-01	4.88E-01
4-Guanidinobutanoate	0.90 (0.82, 0.99)	3.08E-02	2.62E-01	0.92 (0.83, 1.02)	1.18E-01	8.15E-01	0.97 (0.87, 1.08)	5.85E-01	9.64E-01	1.80E-01
LysoPE (O-18:0)*	0.90 (0.82, 1.00)	4.51E-02	2.85E-01	0.93 (0.85, 1.02)	1.49E-01	8.20E-01	0.93 (0.82, 1.06)	2.70E-01	9.18E-01	5.18E-01
LysoPE (18:0)	0.88 (0.80, 0.97)	8.12E-03	1.63E-01	1.00 (0.91, 1.10)	9.97E-01	9.98E-01	0.89 (0.79, 1.01)	7.70E-02	9.10E-01	8.59E-01
Androstenediol (3β,17β) monosulfate (1)	1.02 (0.93, 1.13)	6.62E-01	8.31E-01	1.09 (0.99, 1.21)	8.25E-02	8.15E-01	1.12 (0.99, 1.27)	7.74E-02	9.10E-01	5.04E-01
Sphinganine	0.88 (0.80, 0.96)	5.43E-03	1.35E-01	0.91 (0.83, 1.01)	6.87E-02	8.15E-01	0.97 (0.87, 1.09)	6.45E-01	9.64E-01	8.52E-02
β-Cryptoxanthin	0.95 (0.87, 1.05)	3.21E-01	6.05E-01	0.90 (0.82, 0.99)	3.76E-02	8.15E-01	0.93 (0.82, 1.05)	2.49E-01	9.18E-01	4.82E-01
PE (18:1/18:2)*	0.91 (0.82, 1.00)	5.25E-02	3.03E-01	0.95 (0.86, 1.06)	3.66E-01	9.16E-01	0.92 (0.82, 1.04)	2.10E-01	9.18E-01	9.45E-01
Dehydroepiandrosterone sulfate (DHEA-S)	1.02 (0.92, 1.13)	7.48E-01	8.72E-01	1.07 (0.96, 1.19)	2.14E-01	8.62E-01	1.18 (1.03, 1.35)	1.50E-02	7.18E-01	1.03E-01
Glutamine	0.91 (0.83, 0.99)	3.64E-02	2.75E-01	0.94 (0.86, 1.04)	2.27E-01	8.72E-01	0.93 (0.82, 1.05)	2.37E-01	9.18E-01	5.97E-01

†Models were adjusted for age, race, education, family history of breast cancer, age at menarche, oral contraceptive use, postmenopausal hormone use, parity and age at first birth.

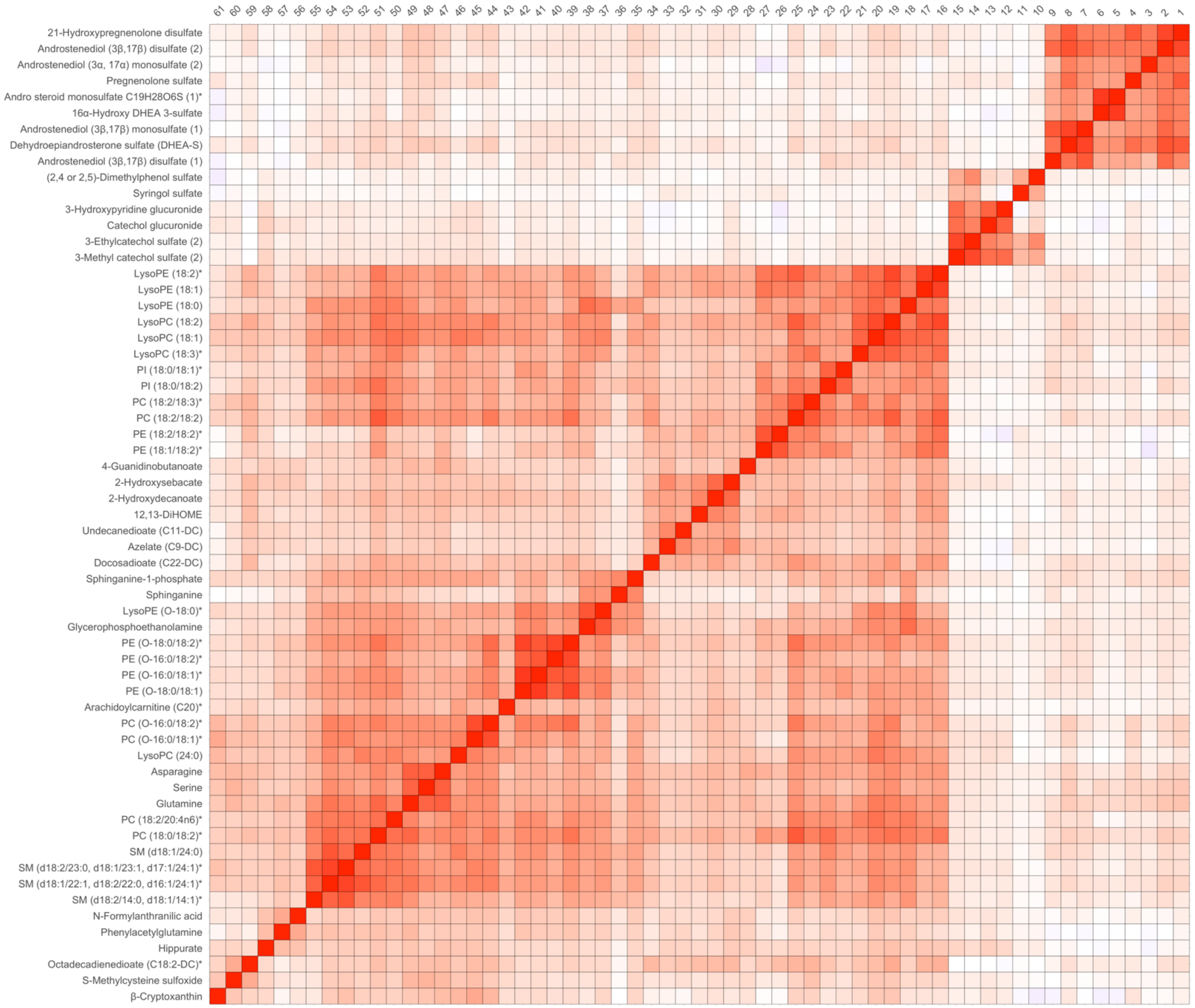

Supplemental Figure 1. Hierarchical heat map based on Pearson correlation coefficients of metabolites associated with breast cancer at FDR<0.20 in analyses adjusted for age, race, education, family history of breast cancer, age at menarche, OC use, and parity.
